# HIFU‐Driven Targeted Pyroptosis Therapy in Basal‐Like Breast Cancer

**DOI:** 10.1002/advs.202503830

**Published:** 2025-09-14

**Authors:** Xiaomin Su, Yang Wang, Xifeng Qin, Yaqiong Xiao, Boshu Ouyang, Lina Hu, Lin Kang, Ruizhe Xu, Ce Xu, Zanya Sun, Chenyu Sun, Huishu Guo, Zhiqing Pang, Shun Shen

**Affiliations:** ^1^ Central Laboratory First Affiliated Hospital Institute (College) of Integrative Medicine Dalian Medical University Dalian 116021 China; ^2^ Pharmacy Department Shanghai Pudong Hospital Fudan University Pudong Medical Center Shanghai 201399 China; ^3^ School of Pharmacy & Key Laboratory of Smart Drug Delivery Fudan University Shanghai 201203 China; ^4^ ShuGuang Hospital Affiliated to Shanghai University of Traditional Chinese Medicine Shanghai 201203 P. R. China; ^5^ Department of Oncology Fudan University Pudong Medical Center Shanghai 201399 P. R. China

**Keywords:** bioinformatics, BLBC, cathepsin L, HIFU, pyroptosis

## Abstract

The high heterogeneity of basal‐like breast cancer (BLBC) and the absence of effective therapeutic targets pose ongoing treatment challenges. Pyroptosis, a type of cell death characterized by cell swelling and membrane perforation, offers a promising therapeutic weathervane for BLBC, particularly when induced by physical therapies. In this study, a high‐intensity focused ultrasound (HIFU)‐driven targeted pyroptosis strategy is developed for BLBC therapy. Through integration of HIFU‐driven gene regulation analysis and bioinformatics analysis of pyroptosis‐related genes from the TCGA dataset, 20 potential pyroptosis inducers are identified to work synergistically with HIFU. Mitoxantrone, a promising inducer, is encapsulated in platelet membrane‐hybridized liposomes to enhance targeted delivery and therapeutic efficacy. Importantly, the combination of HIFU and Plp enhanced tumor delivery of liposomes by 2.46 fold and dramatically inhibited tumor growth, with 50% of female BALB/c mice remaining tumor‐free compared to liposome‐only treatment. Mechanistically, HIFU significantly downregulated the expression of histone deacetylases 4 and 9, while promoting cathepsin‐L (CTSL) gene transcription. Simultaneously, Plp and HIFU synergistically suppressed BCL‐2 via CTSL, increasing ROS production. This activated Caspase8 and the NLRP3 inflammasome, leading to GSDMC cleavage and initiating pyroptosis. Collectively, this study provides an innovative pyroptosis therapy strategy combining physical treatment and chemotherapy for BLBC and other refractory diseases.

## Graphical abstract

This study presents a HIFU‐driven pyroptosis strategy for BLBC treatment. Combining HIFU with platelet‐membrane hybridized liposomes encapsulating mitoxantrone enhances tumor targeting and efficacy. The synergistic treatment modulates key epigenetic regulators and activates the pyroptotic pathway through ROS generation and inflammasome signaling, achieving significant tumor suppression. This approach offers a promising physical‐chemical combination therapy for refractory cancers.

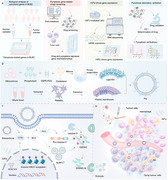

## 1 Introduction

Breast cancer is currently the most prevalent cancer worldwide, accounting for 12% of all new cancer diagnoses annually, and remains the leading cause of cancer‐related morbidity and mortality.^[^ ^1^, ^2^ ^]^ BLBC, which represents an estimated 15–20% of all breast cancers, is defined by its distinct transcriptional profile and is characterized by high proliferative activity and dysregulated cell cycle checkpoints.^[^ ^3^ ^]^ ≈75% of BLBC cases are classified as triple‐negative breast cancers (TNBC), which are characterized by the absence of estrogen receptor, progesterone receptor, and human epidermal growth factor receptor 2 expression.^[^ ^4^, ^5^ ^]^ In comparison with other breast cancer subtypes, BLBC displays pronounced heterogeneity and aggressiveness, marked by poor differentiation, high invasiveness, and an elevated tendency for early recurrence and metastasis.^[^ ^6^ ^]^ The absence of specific receptor targets renders BLBC resistant to conventional endocrine and HER2‐targeted therapies, posing significant treatment challenges and contributing to a generally poorer patient prognosis. In contemporary clinical practice, chemotherapy and surgery remain the cornerstone of BLBC treatment, while newer therapies, such as poly(ADP‐ribose) polymerase (PARP) inhibitors, immune checkpoint inhibitors, and antibody‐drug conjugates (ADCs), have recently been approved for BLBC treatment and have significantly improved patient outcomes.^[^ ^7^, ^8^, ^9^ ^]^ Nevertheless, many BLBC patients may develop resistance to PARP inhibitors over time, complicating long‐term tumor control, which may result from compensatory activation of DNA repair mechanisms or the escape of cancer cells through alternative repair pathways.^[^ ^10^ ^]^ Notably, the clinical efficacy of checkpoint inhibitors in combination with chemotherapy depends on the response rate in BLBC patients. Furthermore, the tumor microenvironment (TME) of metastatic BLBC is highly heterogeneous compared to primary tumors, which weakens optimism about cancer immunotherapy.^[^ ^11^ ^]^ Consequently, developing innovative and effective strategies to overcome these challenges and enhance treatment outcomes for BLBC is critically important.

Pyroptosis is a form of programmed cell death triggered by the activation of inflammasomes, typically characterized by cell membrane perforation and the release of inflammatory cytokines.^[^ ^12^ ^]^ Studies indicated that activating pyroptosis altered the TME by releasing inflammatory factors, which in turn inhibited tumor cell proliferation, migration, and metastatic potential.^[^ ^13^ ^]^ Pyroptosis holds significant promise in cancer therapy, particularly for treating cancers like BLBC that lack effective therapeutic targets.^[^ ^14^ ^]^ For instance, the researchers created cationic liposomes derived from DOTAP and DOPE for the simultaneous delivery of a GSDME expression plasmid and manganese carbonyl. Surface modification with polyethylene glycol chains and RGDfK (cRGD)‐targeting peptides enhanced the accumulation of these nanocarriers in tumors, ultimately inducing pyroptosis in TNBC cells.^[^ ^15^ ^]^ Furthermore, the research team developed intermetallic compounds, Pd2Sn@GOx‐SP, modified with glucose oxidase and soybean phosphatidylinositol, to act as pyroptosis inducers. These compounds promoted pyroptosis through cascade biocatalysis, reshaped the TME enhanced tumor cell immunogenicity, and triggered pyroptosis via cystine accumulation.^[^ ^16^ ^]^ The pursuit of novel inducers that can specifically target and stimulate pyroptosis in BLBC is thus of high relevance and value in advancing precision therapies for BLBC. Meanwhile, recent studies underscore the increasing importance of smart healthcare systems based on omics in personalized medicine. By integrating and analyzing multi‐omics data—such as genomics, proteomics, and metabolomics—these technologies facilitate the delivery of precision healthcare tailored to individual patients.^[^ ^17^, ^18^ ^]^ Genomics technology, in particular, allows for the processing of large‐scale biological data to identify molecular targets associated with disease susceptibility and predict individual drug responses, thereby assisting in disease diagnosis, prognosis, and treatment selection.^[^ ^19^ ^]^ For instance, genome sequencing can identify critical mutations in tumor cells that promote tumor progression, directly informing the choice of targeted therapies. In BLBC treatment, where effective targets are scarce, omics‐based screening for pyroptosis‐related genes could open new pathways for developing precise and effective therapeutic strategies.

High‐intensity focused ultrasound (HIFU) is a novel, non‐invasive ablation technique that employs focused ultrasound to target lesions through a combination of thermal and mechanical effects, and has emerged as a powerful method for cancer treatment.^[^ ^20^, ^21^ ^]^ The principle of HIFU leverages the exceptional tissue focusability, directionality, and energy penetration of ultrasound. Concentrating ultrasound energy at a targeted site induces rapid coagulation and deformation of tissue cells within the focal area. The resulting necrotic tissue gradually undergoes fibrosis, leading to a reduction in volume and the alleviation, or even resolution, of clinical symptoms, thereby achieving the desired therapeutic effect.^[^ ^22^, ^23^ ^]^ After HIFU destroys the diseased tissue, substances such as tumor‐associated antigens are released, which activate the host immune system. This process induces immunogenic cell death in tumors and promotes dendritic cell maturation as well as the activation of CD8^+^ T cells.^[^ ^24^, ^25^ ^]^ For instance, a study demonstrated that HIFU can mechanically fragment tumors, converting immunologically “cold” tumors into reactive “hot” tumors. When combined with αCTLA‐4 and αPD‐L1 antibodies, this approach significantly boosts anti‐tumor responses and markedly prolongs survival in mice.^[^ ^26^ ^]^ Another study also described a novel synergistic regimen involving HIFU and immunomodulatory biomimetic perfluorocarbon nanoparticles (M@P‐SOP), which help stimulate immunogenic cell death in tumor cells while mitigating the immune‐suppressive tumor microenvironment. Specifically, guided by photoacoustic/magnetic resonance imaging/ultrasound multimodal imaging, M@P‐SOP accumulates significantly in tumors, greatly enhancing the tumor‐killing effects of HIFU and inducing robust immunogenic cell death. Simultaneously, M@P‐SOP releases oxygen to mitigate tumor hypoxia, converting M2‐type macrophages into anti‐tumor M1‐type macrophages. When combined with anti‐PD‐L1 therapy, this approach further expands the anti‐tumor immune response systemically, effectively inhibiting tumor growth.^[^ ^27^ ^]^ Moreover, HIFU‐induced shear stress and cavitation effects have been shown to enhance vascular permeability, improving drug penetration. Clinically, MR‐guided HIFU combined with intravenous microbubble drug delivery could temporarily open the blood‐brain barrier (BBB) in patients with neurodegenerative diseases or brain tumors. This provides clues for BBB‐open drug delivery in the treatment of Parkinson's disease and other neurodegenerative disorders.^[^ ^28^ ^]^ Consequently, as an adjunctive therapy, HIFU has the potential to enhance the efficacy of drug delivery systems, facilitating the uptake of released drugs by tumor cells, promoting efficient drug accumulation in tumors, and enhancing the effects of chemotherapy and immunotherapy. Nevertheless, whether HIFU can regulate the expression of pyroptosis‐related genes, thereby enhancing the efficacy of pyroptosis‐based tumor therapy and the underlying mechanisms involved, remains unclear and requires further investigation.

Within heterogeneous tumors, achieving targeted and specific modulation of liposome‐encapsulated drugs for select cell populations is crucial for enhancing antitumor therapeutic efficacy. Recent studies have demonstrated that the cell membrane coating retains the intrinsic properties of its source cells, leading to extensive research on constructing cell membrane‐hybridized liposomes using various cellular origins, including erythrocytes, platelets, and macrophages.^[^ ^29^ ^]^ These hybrid liposomes can effectively evade immune surveillance, particularly those derived from erythrocytes, immune cells, and platelets, which enable liposomes to “camouflage” as natural cells in the body. This prevents their recognition and clearance by immune cells such as macrophages and T cells, thereby extending the drug's half‐life in circulation.^[^ ^30^ ^]^ Biomimetic liposomes generated through this strategy inherit the specific biological functions of their source cells and integrate the advantages of various membrane‐associated proteins and molecules, conferring enhanced biocompatibility and reduced immunogenicity. This allows them to evade immune clearance, prolong circulation time, and leverage membrane protein ligand‐receptor recognition mechanisms to achieve targeted drug delivery, thereby addressing some of the limitations faced by conventional tumor therapies.^[^ ^31^ ^]^ Among these approaches, compared to erythrocyte and leukocyte membranes, biomimetic platelet membrane‐hybridized liposomes offer distinct advantages, including a rapid response to vascular injury and the ability to recognize and interact with circulating tumor cells, making them widely utilized in tumor‐targeted therapy. Furthermore, platelet membranes express a variety of protein molecules (e.g., selectins and integrins) that can bind to tumor cell surface receptors, thereby enhancing the delivery of pyroptosis‐inducing drugs to tumor sites through their immune evasion and tumor‐targeting capabilities.^[^ ^32^ ^]^

**Scheme 1:**
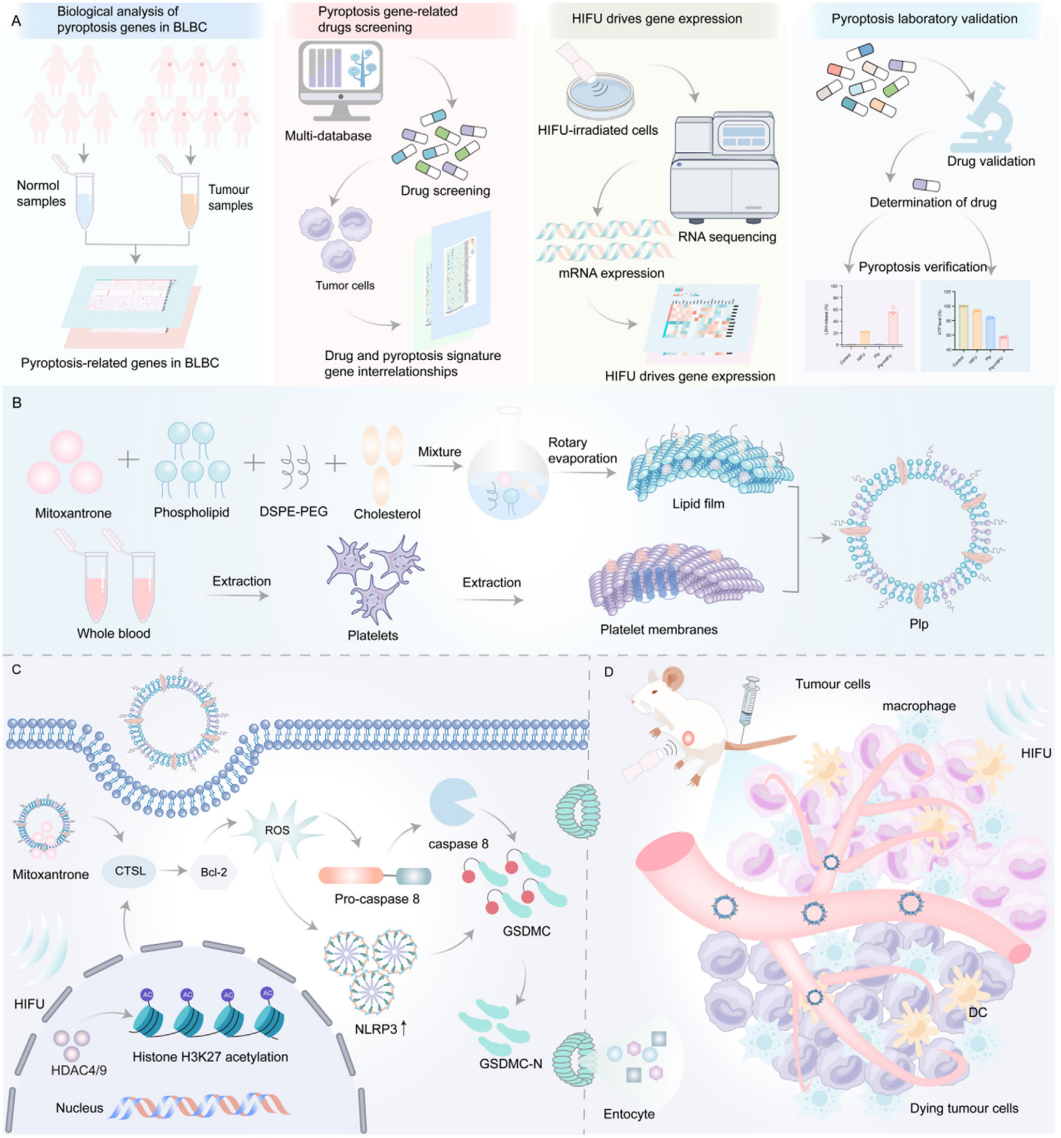
Illustrates the screening of pyroptosis inducers and the mechanistic study of combined HIFU treatment for BLBC. A) The process of screening and identifying MIT, a novel pyroptosis inducer for the treatment of BLBC. B) The preparation process of platelet membrane‐hybridized liposomes loaded with MIT. C) Mechanism of HIFU amplification of Plp‐induced pyroptosis of BLBC cells. D) The schematic representation of tumor cell pyroptosis induced by Plp and HIFU.

In this study, we developed an HIFU‐driven targeted pyroptosis strategy for BLBC therapy. This strategy integrates HIFU‐mediated gene regulation, bioinformatics, multi‐database drug screening, and experimental validation to facilitate the discovery and optimization of pyroptosis drugs synergizing with HIFU (**Scheme** **1**). Specifically, bioinformatics analysis of transcriptomics was initially employed to identify key pyroptosis‐related genes associated with BLBC and the corresponding drugs by comparing BLBC tumors with adjacent normal tissue samples from TCGA. Subsequently, mRNA sequencing was conducted to analyze the expression changes of key pyroptosis‐associated genes following HIFU treatment, leading to the identification of 20 potential pyroptosis inducers to work synergistically with HIFU based on the HIFU‐gene‐drug interaction network. Mitoxantrone (MIT) was validated as the most promising inducer of pyroptosis and was encapsulated in platelet membrane‐hybridized liposomes (Plp) to enhance its targeted delivery and therapeutic efficacy. Surprisingly, the combination of HIFU and Plp led to a significant enhancement in the tumor delivery of liposomes by 2.78 fold and significantly inhibited tumor growth, with 50% of mice being tumor‐free compared to those treated with liposomes alone. Then, the underlying mechanisms of HIFU enhancing pyroptosis therapy of Plp were discovered. Mechanistically, HIFU treatment significantly reduced the expression of histone deacetylases 4 and 9 (HDAC4 and HDAC9), leading to increased acetylation of histone H3K27 (H3K27Ac) and promoting the transcription and activation of the cathepsin L (CTSL) gene. Simultaneously, Plp and HIFU acted synergistically to suppress BCL‐2 expression via CTSL, leading to increased ROS production. This, in turn, activated caspase‐8 and the NLRP3 inflammasome, triggering the N‐terminal cleavage of Gasdermin C (GSDMC) and initiating pyroptosis. Taken together, this study offers an innovative methodological approach for drug development targeting BLBC and other refractory diseases.

## 2 Results and Discussion

### 2.1 Identification of Key Pyroptosis‐Associated Genes and Drugs in a BLBC Cohort

**Figure 1:**
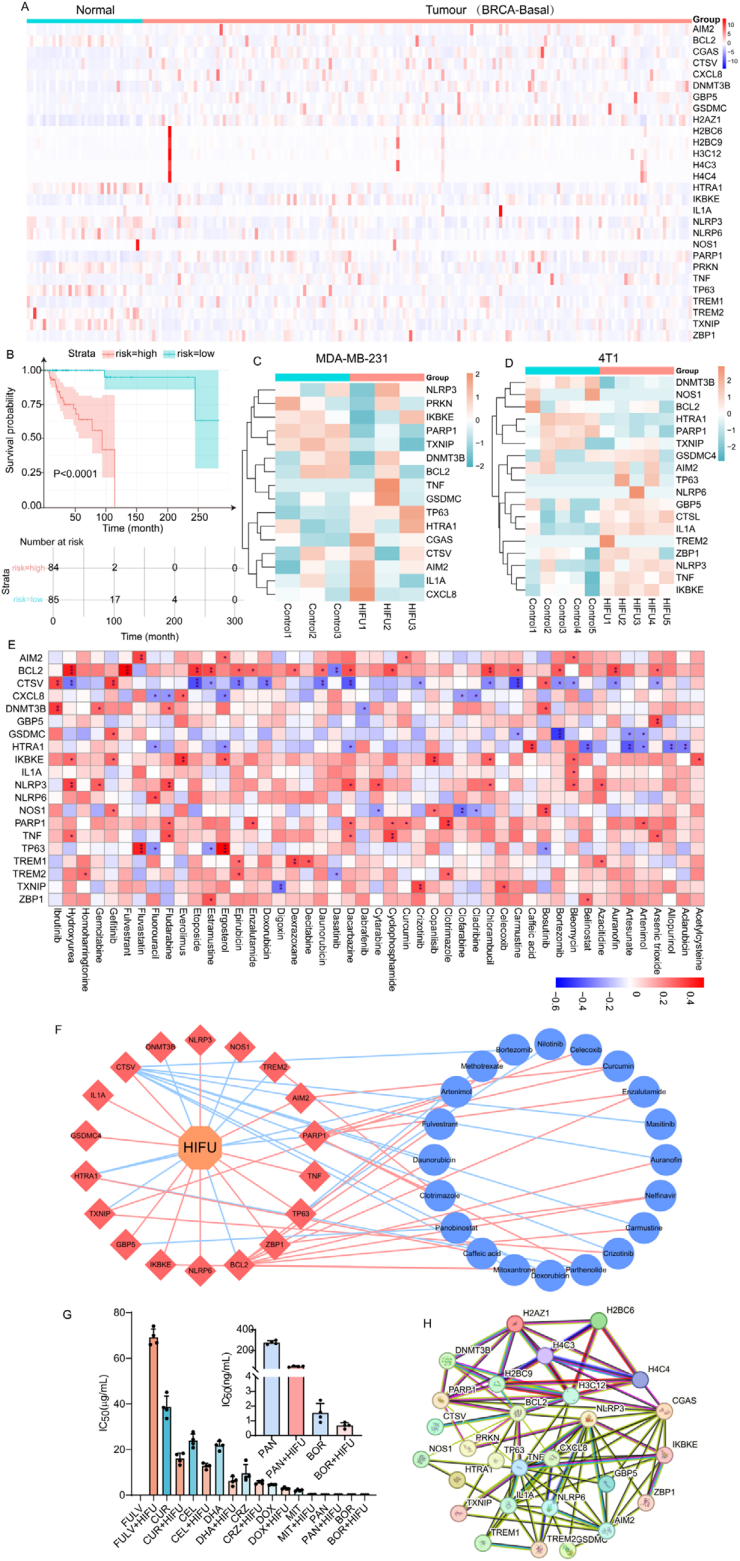
Identification of pyroptosis‐related genes and potential drug candidates in the BLBC Cohort. A) Heatmap analysis of pyroptosis‐related gene expression in normal and tumor tissues of BLBC from the TCGA database. B) Kaplan‐Meier overall survival curves of BLBC patients stratified by pyroptosis risk (high versus low risk; P < 0.0001). Heat maps analysis of expression of genes related to pyroptosis in MDA‐MB‐231 cells (C) and 4T1 cells (D) after HIFU irradiation. E) Correlation between IC50 values of 94 predicted pyroptosis‐inducing agents and the expression levels of pyroptosis‐regulated genes (^*^ *p* < 0.05, ^**^ *p* < 0.01, ^***^ *p* < 0.001). F) Network diagram of HIFU‐gene‐drug interactions in 4T1 cells (red line:101678e006226e0037171016 positive correlation, blue line: negative correlation). G) IC50 values of different drugs in combination with HIFU. H) Protein‐protein interaction (PPI) network of pyroptosis‐regulated genes.

To assess the characteristics of pyroptosis in BLBC and explore potential opportunities for pyroptosis therapy, we utilized bioinformatics techniques to analyze transcriptomic data from 169 BLBC patients in the TCGA database. Gene Set Enrichment Analysis (GSEA) was employed to summarize pyroptosis‐related genes, and their expression levels in BLBC were compared to those in normal breast tissues. The analysis revealed that 28 genes (AIM2, BCL2, CGAS, CTSV, CXCL8, DNMT3B, GBP5, GSDMC, H2AZ1, H2BC6, H2BC9, H3C12, H4C3, H4C4, HTRA1, IKBKE, IL1A, NLRP3, NLRP6, NOS1, PARP1, PRKN, TNF, TP63, TREM1, TREM2, TXNIP, ZBP1) were significantly differentially expressed across 169 BLBC and 36 normal breast tissue samples (**Figure** **1**A; Figure S1, Supporting Information). To better understand the functional roles of these genes, we performed Gene Ontology (GO) enrichment analysis, revealing key biological processes and pathways linked to pyroptosis, such as the pyroptosis signaling pathway, positive regulation of cytokine‐mediated signaling, defense response activation, inflammatory vesicle complex assembly, and regulation of oxidative stress‐induced neuronal apoptosis. However, the enrichment score of 3.0 in the pyroptosis signaling pathway was significantly higher than the other signaling pathways, suggesting that pyroptosis mechanisms may serve as important targets for BLBC treatment (Figure S2, Supporting Information). Furthermore, to elucidate the interactions among these 28 pyroptosis regulators in BLBC, we constructed correlation maps and protein‐protein interaction (PPI) networks, offering a comprehensive view of their interrelationships (Figure 1H). Additionally, the forest plot illustrates the hazard ratios (HR) and 95% confidence intervals for 28 pyroptosis‐related genes in relation to recurrence‐free survival (RFS) in BLBC patients. The results showed that genes with confidence intervals not crossing the baseline were considered statistically significant and were found to be correlated with BLBC recurrence. The Akaike Information Criterion (AIC) value and consistency index (C‐index) for the model were 143.75 and 0.96, respectively, demonstrating strong model fit and predictive precision. Notably, ZBP1 and NLRP6 exhibited HR values less than 1, indicating that elevated expression of these genes might be linked to a reduced risk of recurrence, while higher expression of PRKN was correlated with a significantly increased risk (Figure S3, Supporting Information). Survival analysis further validated the significance of these 28 genes in predicting patient outcomes. The survival curves revealed that the low‐risk group exhibited significantly better RFS compared to the high‐risk group, with statistical analysis confirming a p‐value of less than 0.001 (Figure 1B). These results underscore the potential of pyroptosis‐related genes as reliable biomarkers for predicting BLBC recurrence and prognosis, as evidenced by the superior survival outcomes observed in low‐risk patients. Collectively, these 28 pyroptosis‐related genes are crucial in the progression and prognosis of BLBC, laying the groundwork for the development of precision‐based therapeutic approaches. Subsequently, we employed various drug databases and filtering techniques to identify potential drug candidates linked to the 28 key pyroptosis regulators. The Comparative Toxicogenomics Database (CTD) was employed to identify drugs associated with at least one of the regulators, and an average z‐score for drug activity (DTP NCI‐60) was calculated based on RNA expression of the pyroptosis regulators. This approach yielded 94 potential drug candidates from a total of 25147 compounds (Figure 1E; Figure S4, Supporting Information). Conspicuously, the expression data for 8 genes (CGAS, H2AZ1, H2BC6, H2BC9, H3C12, H4C3, H4C4, PRKN) were unavailable in the NCI‐60 dataset, and our analysis focused on the remaining 20 genes to examine their interactions with therapeutic agents.

We initially assessed the proliferation and toxicity of cells after exposure to varying powers and durations of HIFU. The results demonstrated a significant decrease in cell viability with increasing power and exposure time. Specifically, at 9.2 W, cell viability was 85.06% after 20 s but dropped to 20.28% after 40 s (Figure S5A, Supporting Information). Analysis of frequency effects revealed that high‐frequency ultrasound (10 MHz) exhibited a stronger cytotoxic effect compared to lower frequencies (e.g., 8.5 MHz). After 60 s of irradiation, the cell survival rate in the 10 MHz group was significantly reduced to 20.46%, markedly lower than that in the 8.5 MHz group. Furthermore, analysis of pulse parameters showed that increasing the pulse repetition frequency to 1000 Hz enhanced the time‐dependent cytotoxic effect, as indicated by a steeper decline in cell viability with prolonged exposure (Figure S5B,C, Supporting Information). To maintain reasonable cell viability, we selected an irradiation condition of 8.4 W for 30 seconds for subsequent experiments. To investigate the transcriptomic response of 4T1 and MDA‐MB‐231 cells from BLBC to HIFU treatment, we analyzed mRNA expression profiles of these cells after HIFU treatment. As shown in Figure 1C,D, HIFU treatment induced significant alterations in the expression of 20 key pyroptosis‐related genes, suggesting that HIFU combined with pyroptosis drugs may offer a promising therapeutic strategy for BLBC. Subsequently, potential pyroptosis drugs were identified by analyzing the HIFU‐gene‐drug interaction network. As shown in Figure 1F and Figure S6 (Supporting Information), the red line in the HIFU‐gene‐drug interaction network represents a positive correlation, while the blue line indicates a negative correlation. A negative gene‐drug correlation combined with a positive HIFU‐gene correlation suggests that HIFU may enhance drug efficacy. For instance, Panobinostat (PAN) was found to be negatively correlated with the expression of GBP5, meaning that higher expression of GBP5 led to a lower IC50 value for the drug. Given that HIFU significantly elevated GBP5 expression, we hypothesized a synergistic effect between PAN and HIFU (Figure 1E). This interaction facilitated the identification of 20 potential pyroptosis drugs suitable for combination with HIFU in the treatment of BLBC (Figure S7, Supporting Information). To further validate this approach, we selected 9 candidate drugs for cell proliferation and toxicity assays, based on their individual properties (Figures S8 and S9, Supporting Information). The results showed that a more pronounced reduction in cell viability was produced when the drugs were combined with HIFU, with Panobinostat (PAN), Bortzeomib (BOR), and mitoxantrone (MIT) exhibiting particularly strong inhibitory effects (Figure S8, Supporting Information). To assess the efficacy of drug‐HIFU combinations, we determined the half‐maximal inhibitory concentration (IC50) in 4T1 cells. The combination of these drugs with HIFU markedly reduced the IC50 values compared to either treatment alone (Figure 1G). Specifically, the IC50 for Fulvestrant and Artenimol alone were >200 and 38.66 µM, respectively, while the IC50 for HIFU alone was 38.38 W with 30 S of irradiation. In contrast, the IC50 values decreased to 69.22 and 16.06 µM when the drugs were combined with HIFU, demonstrating enhanced therapeutic efficacy and increased cellular sensitivity (Figure 1G). Remarkably, HIFU combined with PAN, BOR, and MIT demonstrated excellent antitumor effects, with IC50 values reduced by 6.21, 2.29, and 12.91 fold, respectively. In addition, MDA‐MB‐231 cells were employed to further investigate whether HIFU could enhance the antitumor effects of these drugs (Figure S9, Supporting Information). The results showed that HIFU significantly improved the efficacy of the drugs to varying degrees by reducing their IC50 values. Specifically, the combination of HIFU with PAN, BOR, and MIT resulted in a 5.16, 2.08, and 12.57 fold decrease in IC50 values, respectively, emphasizing the potential of HIFU in combination with chemotherapeutic drugs to enhance therapeutic outcomes (Figure S10, Supporting Information). Considering the above results and the clinical applications of these drugs—PAN for multiple myeloma, BOR for multiple myeloma, and MIT as a potent anthracycline chemotherapy agent effective against various cancers—MIT was prioritized for further investigation in this study. MIT functions as a topoisomerase II inhibitor, inducing DNA damage through dual mechanisms: intercalation into DNA and inhibition of topoisomerase II activity, and is widely used in both scientific research and clinical treatment of breast cancer.^[^ ^33^ ^]^

### 2.2 Characterization of Plp and Antitumor Effects of HIFU Combined with Plp

**Figure 2:**
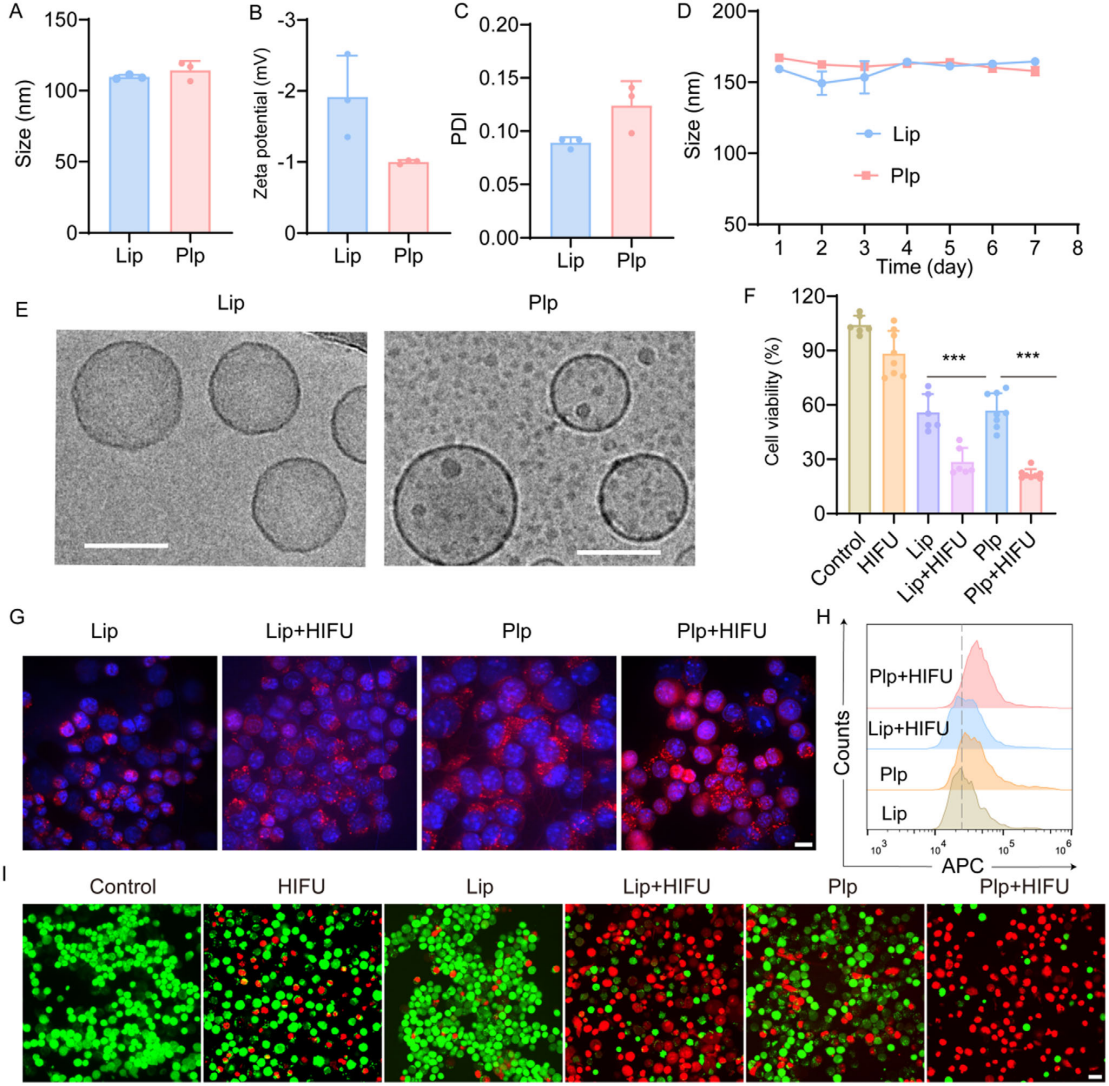
Characterization of Plp and in vitro killing validation of Plp. A) Dynamic light scattering analysis of Lip and Plp particle size (n = 3), (B) surface zeta potential and PDI (C) (n = 3). D) 7‐day stability of Lip and Plp in PBS (n = 3). E) Representative cryo‐TEM images of Lip and Plp. (Scale bar = 100 nm). F) Cell viability after treatment of 4T1 cells in different groups (n = 6). G) Fluorescence images of cellular uptake after 4 h treatment with Lip and Plp, scale bar = 50 µm. H) Flow analysis of cellular uptake after Lip and Plp treatment for 4 h. I) Confocal images of different groups of treated 4T1 cells after co‐staining with Calcein ‐AM (green) and PI (red) (scale bar = 50 µm). Data are presented as mean ± SD. Statistical significance was defined as ^***^ *p* < 0.001.

To enhance tumor drug delivery and prolong its circulation time in vivo, MIT was encapsulated within platelet membrane‐hybrid liposomes and subjected to comprehensive characterization. (Platelet membrane heterogeneous liposome‐loaded MIT is called Plp, and liposome‐loaded MIT is referred to as Lip.) As illustrated in **Figure** **2**A, the diameters of Lip and Plp were 109.70 ± 0.90 nm and 114.3 ± 3.84 nm, respectively, with corresponding zeta potentials of −1.91 ± 0.34 mV and −1.00 ± 0.02 mV (Figure 2B). The polydispersity index (PDI) analysis revealed that Lip exhibited a slightly lower PDI than Plp, indicating a more uniform particle size distribution for Lip, whereas Plp displayed a marginally broader distribution, potentially attributable to the non‐uniform incorporation of platelet membranes into the liposomes (Figure 2C). To assess whether this heterogeneity affected the stability of Plp, particle sizes of both Lip and Plp were monitored over a 7 day period, revealing no significant aggregation and confirming their stability (Figure 2D). Additionally, cryo‐transmission electron microscopy (cryo‐TEM) confirmed that both Lip and Plp presented as vesicle‐like structures with smooth surfaces (Figure 2E). The drug loading and encapsulation efficiency of MIT within platelet membrane‐hybrid liposomes were 8.07 ± 0.01% and 94.78 ± 0.19%, respectively.

Following the successful construction of bionic liposomes, we assessed whether platelet membrane‐coated hybrid liposomes could enhance MIT internalization. As shown in Figure 2H, after 4 h, 4T1 cells exhibited significantly higher uptake of Plp compared to Lip. This enhanced cellular uptake is likely attributed to the interactions between p‐selectin present on the platelet membranes and CD44 receptors on tumor cells.^[^ ^32^, ^34^ ^]^ Flow cytometry corroborated these findings, revealing that cellular uptake in the Plp group was 1.34 fold higher than in the Lip group, underscoring the efficacy of platelet membrane‐hybrid liposomes in promoting internalization (Figure 2H; Figure S11, Supporting Information). Notably, the fluorescence intensity in the Plp+HIFU group exceeded that of the Plp group, suggesting that HIFU enhances Plp uptake (Figure 2G). Given the superior cellular uptake of Plp, we further investigated the cytotoxic effects of Plp combined with HIFU on 4T1 cells. Confocal fluorescence images of 4T1 cells stained with Calcein‐AM and PI after various treatments provided visual evidence of the enhanced anticancer effects of Plp and HIFU. As shown in Figure 2I, the HIFU group exhibited minimal red fluorescence, indicating a weak cytotoxic effect. In contrast, the Plp group displayed more pronounced red fluorescence compared to the Lip group, reflecting increased Plp uptake and enhanced drug‐induced cell death. Crucially, the Plp+HIFU group displayed significantly greater red fluorescence than the Plp group, indicating that HIFU further amplified the anti‐tumor effect of Plp. Additionally, CCK‐8 assay results revealed that cell viability in the Plp+HIFU group was reduced to 21.73%, significantly lower than the 56.90% observed in the Plp group, highlighting that HIFU irradiation enhanced the tumor cell‐killing efficacy of Plp (Figure 2F).

### 2.3 Underlying Mechanisms of HIFU Enhancing the Anti‐Tumor Activity of Plp

**Figure 3:**
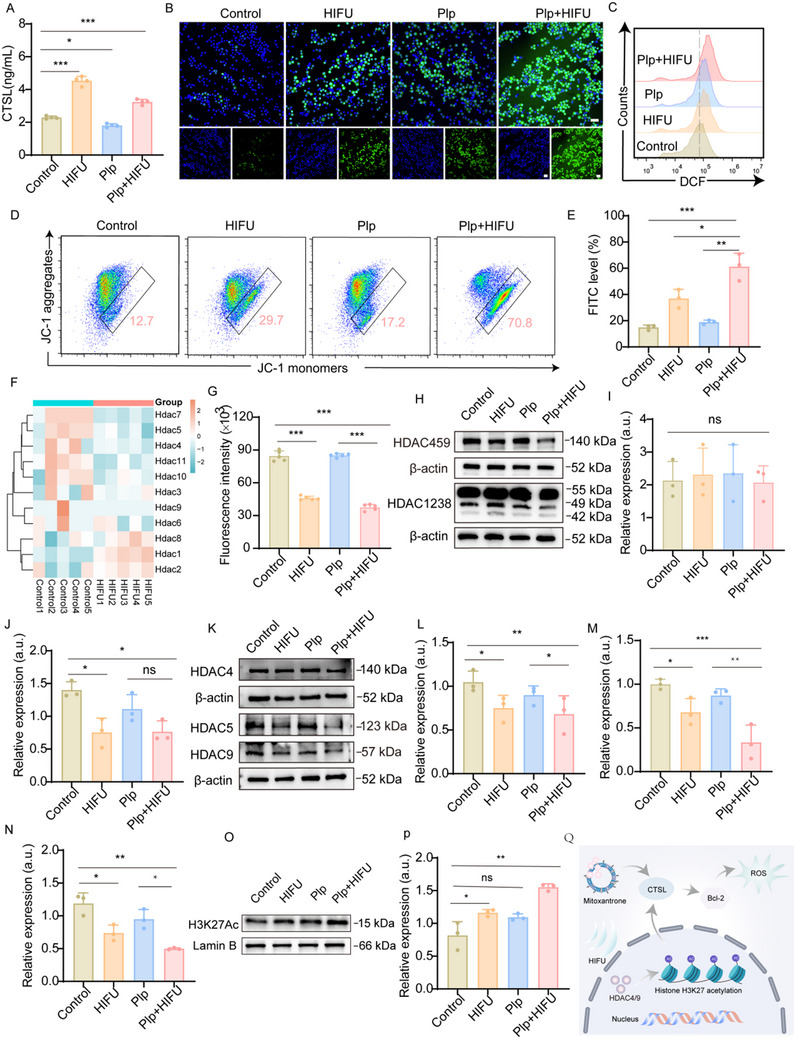
Underlying mechanisms of HIFU‐enhanced anti‐tumor effects of Plp. A) The expression level of CSTL after different treatments was detected by ELISA (n = 4). B) Confocal pictures of DCFH‐DA as a probe to detect ROS generation in different preparations of treated 4T1 cells (scale bar = 50 µm). C) Flow analysis to detect ROS generation in 4T1 cells treated with different agents. D) JC‐1 detection of changes in mitochondrial membrane potential after different treatment formulations and the corresponding quantitative analysis E) (n = 3). F) Heatmap showing transcriptomics analysis of HDACs‐related expression (n = 5). G) Total fluorescence intensity of HDAC after different treatments (n = 5). H) Western blot showed expression of HDAC‐459 and HDAC‐1238 in 4T1 cells and semi‐quantitative analysis of HDAC‐1238 (I) and HDAC‐459 (J) (n = 3). K) Western blot demonstrated expression of HDAC4, HDAC5, and HDAC9 in 4T1 cells and semi‐quantitative analysis of HDAC4 (L), HDAC5 (M), and HDAC9 (N) (n = 3). O) The acetylation level of histone H3K27 and semiquantitative analysis of H3K27Ac after treatment (P) (n = 3). Data are presented as mean ± SD. Statistical significance was defined as ^*^ *p* < 0.05, ^**^ *p* < 0.01, ^***^ *p* < 0.001; “ns” indicates not significant.

Building on the cytotoxic effects of HIFU‐enhanced Plp treatment on 4T1 cells, we further investigated the underlying mechanisms. Initially, the HIFU‐gene‐drug interaction analyses suggested that cathepsin V (CTSV) is a gene co‐regulated by HIFU and MIT. The homologous gene in mice is cathepsin L (CTSL). Consequently, we examined the expression of CTSL under various treatments, and the results showed that HIFU significantly upregulated CTSL expression (**Figure** **3**A), which was consistent with the results of transcriptional analysis (Figure 1D). PPI analysis revealed that CTSV forms complex interactions with multiple key pyroptosis genes, among which Bcl‐2 was identified as a potential downstream regulatory target. This interaction suggests that CTSV may play a role in regulating the Bcl‐2‐related pyroptosis pathway or other biological processes (Figure 1F). To further validate this hypothesis, western blotting was performed to analyze Bcl‐2 expression under different treatments. The results showed that both HIFU and Plp significantly reduced Bcl‐2 expression, with an even more pronounced reduction observed when HIFU was combined with Plp (Figure S12, Supporting Information). Previous studies had shown that Bcl‐2 played a crucial role in creating an optimal environment for cell survival by maintaining mitochondrial membrane stability and regulating mitochondrial respiration to meet energy demands. Bcl‐2 prevented mitochondrial membrane perforation and limited electron leakage into the cytoplasm, thereby reducing ROS production.^[^ ^35^ ^]^ However, when Bcl‐2 levels were decreased, the formation of respiratory chain supercomplexes was inhibited, leading to increased ROS production.^[^ ^36^ ^]^ Based on these findings, we hypothesize that the combination of Plp and HIFU destabilizes the mitochondrial membrane by reducing Bcl‐2 expression, which induces excessive ROS production and ultimately drives tumor cell death through oxidative stress. To validate this hypothesis, intracellular ROS levels were monitored using the fluorescent probe DCFH‐DA. This probe is cell membrane permeable and non‐fluorescent until hydrolyzed by cytosolic esterases into DCFH, which is subsequently rapidly oxidized into the highly fluorescent compound DCF. The intensity of green fluorescence emitted by DCF is proportional to the amount of ROS produced. As shown in Figure 3C and Figure S13 (Supporting Information), the fluorescence intensity in the HIFU group was 1.32 times that of the control. Notably, the Plp+HIFU group exhibited fluorescence intensities that were 1.36 and 1.53 fold greater than those of the Plp and HIFU groups, respectively. These results underscore the significant enhancement of ROS production through the synergistic interaction of Plp and HIFU. To further visualize ROS generation, confocal microscopy was employed to analyze 4T1 cells subjected to various treatments. As shown in Figure 3B, HIFU exhibited weak green fluorescence, Plp displayed moderate green fluorescence, and Plp+HIFU resulted in strong green fluorescence, aligning with the flow cytometry result. Given that mitochondria are a primary source of ROS, excessive ROS production can impair mitochondrial function, leading to a reduction in mitochondrial membrane potential (ΔΨm). To investigate changes in ΔΨm, we used JC‐1, a probe that aggregates in mitochondria and emits red fluorescence under high ΔΨm conditions. In contrast, when ΔΨm decreases, JC‐1 remains in its monomeric form, emitting green fluorescence. As shown in Figure 3D,E, green fluorescence levels were 36.9% and 18.87% following HIFU or Plp treatment, respectively. Strikingly, treatment with Plp+HIFU increased green fluorescence to 61.23%, indicating significant mitochondrial damage mediated by ROS generated through the synergistic action of Plp and HIFU.

Previous studies have demonstrated that histone acetylation primarily occurs on lysine residues. Lysine side chains contain amino groups that are positively charged under physiological conditions, allowing them to bind tightly to DNA's negatively charged phosphate groups. Acetylation neutralizes this positive charge, thereby reducing the interaction between histones and DNA, loosening the chromatin structure, and facilitating gene expression. Histone deacetylases (HDACs) play a crucial role in reversing this process.^[^ ^37^, ^38^ ^]^ To further analyze whether the increase in CTSL gene expression after HIFU treatment is related to HDAC, transcriptomic analysis was conducted to examine HDAC expression levels after HIFU. The results revealed that most HDAC levels were reduced following HIFU treatment, suggesting that the upregulation of the CTSL gene may be associated with HIFU (Figure 3F). Subsequently, we employed a fluorescent biotin assay to measure the total HDAC expression under different treatment conditions. Surprisingly, the fluorescence intensity of HDAC was significantly reduced in the HIFU‐treated group compared to the control group, while no significant difference was observed between the Plp‐treated group and the control group. This finding indicates that HIFU effectively reduces HDAC expression (Figure 3G). However, which specific HDAC isoform plays a pivotal role in regulating CTSL gene expression after HIFU treatment is unclear and requires further verification. Therefore, western blot analysis was performed to examine the changes in the activity of Class I and Class II HDACs at the protein level, as shown in Figure 3H,I,J. Notably, the expression of Class II HDAC459 was significantly reduced after HIFU treatment, while Class I HDAC1238 showed no obvious changes. These results suggest that different HDAC types may exhibit distinct responses to HIFU, which could be attributed to their intracellular functions and regulatory mechanisms. Research has shown that Class I HDACs are primarily localized in the nucleus, where they directly participate in the acetylation modification of DNA and histones, thereby influencing transcriptional regulation. In contrast, Class II HDACs have more dynamic localization, shuttling between the nucleus and cytoplasm, enabling them to respond to various cellular signals and stressors while regulating diverse biological processes.^[^ ^39^, ^40^ ^]^ As an external physical stimulus, HIFU may affect intracellular signaling pathways and trigger cellular stress responses. Given that Class II HDACs are closely associated with cellular stress responses, cell migration, and morphological changes, it is plausible that HIFU stimulation preferentially regulates the expression of Class II HDACs. We then further investigated which specific Class II HDAC subtype plays a critical role in gene regulation following HIFU treatment. Using Western blot analysis, we examined the expression of HDAC4, HDAC5, and HDAC9 under different treatment conditions (Figure 3K–N). The results demonstrated that the expression of HDAC4, HDAC5, and HDAC9 was reduced to different degrees in the HIFU‐treated group, suggesting that HIFU inhibited these HDACs, which may increase histone acetylation and regulate the transcriptional activity of related genes.

Furthermore, genome browser analysis revealed significant histone H3K27 acetylation (H3K27Ac) signals in the upstream region of the CTSV gene, which are typically associated with active regulatory elements such as promoters and enhancers. Specifically, prominent H3K27Ac peaks were observed near the transcription start site of the CTSV gene, indicating that this chromatin region is in an open state and may have regulatory functions in transcriptional activity. Annotation of candidate cis‐regulatory elements (cCREs) from the ENCODE database revealed that this region is marked as both an active enhancer (yellow) and a promoter (red), further supporting the hypothesis that enhancer‐promoter interactions in this region may regulate CTSV gene expression (Figure S14, Supporting Information). Based on these observations, we further assessed H3K27Ac levels after HIFU treatment, as shown in Figure 3O,P. Compared to the control group, H3K27Ac levels were significantly elevated in the HIFU‐treated and Plp+HIFU‐treated groups, while no significant changes were observed in the Plp group. These results indicate that the reduced expression of HDAC4 and HDAC9, which are key members of Class IIa HDACs, significantly contributes to the increase in H3K27Ac levels. H3K27Ac is an epigenetic modification strongly associated with chromatin relaxation and active gene transcription. Thus, the downregulation of HDAC4 and HDAC9 may promote chromatin decondensation and activation of target genes by enhancing H3K27Ac levels.^[^ ^41^ ^]^ Schematic diagram of the mechanism by which HIFU enhances the anti‐tumor effect of Plp (Figure 3Q). Taken together, these experimental results suggest that HIFU treatment decreases the expression of HDAC4 and HDAC9, thereby reducing deacetylation activity and promoting the accumulation of H3K27Ac. This, in turn, may have enhanced chromatin openness and facilitated the transcriptional activity of the CTSL gene. Comprehensive analysis of these findings suggests that HIFU may enhance Plp‐induced cell death by downregulating HDAC expression and promoting the transcription of CTSL. Furthermore, this process is negatively correlated with MIT treatment, as MIT increases CTSL gene expression and decreases the IC50 value. However, the specific role of HDAC4 or HDAC9 in regulating the expression of CTSL genes will be further investigated in detail in a separate study.

### 2.4 Augmentation of Plp‐Induced Pyroptosis by HIFU

**Figure 4:**
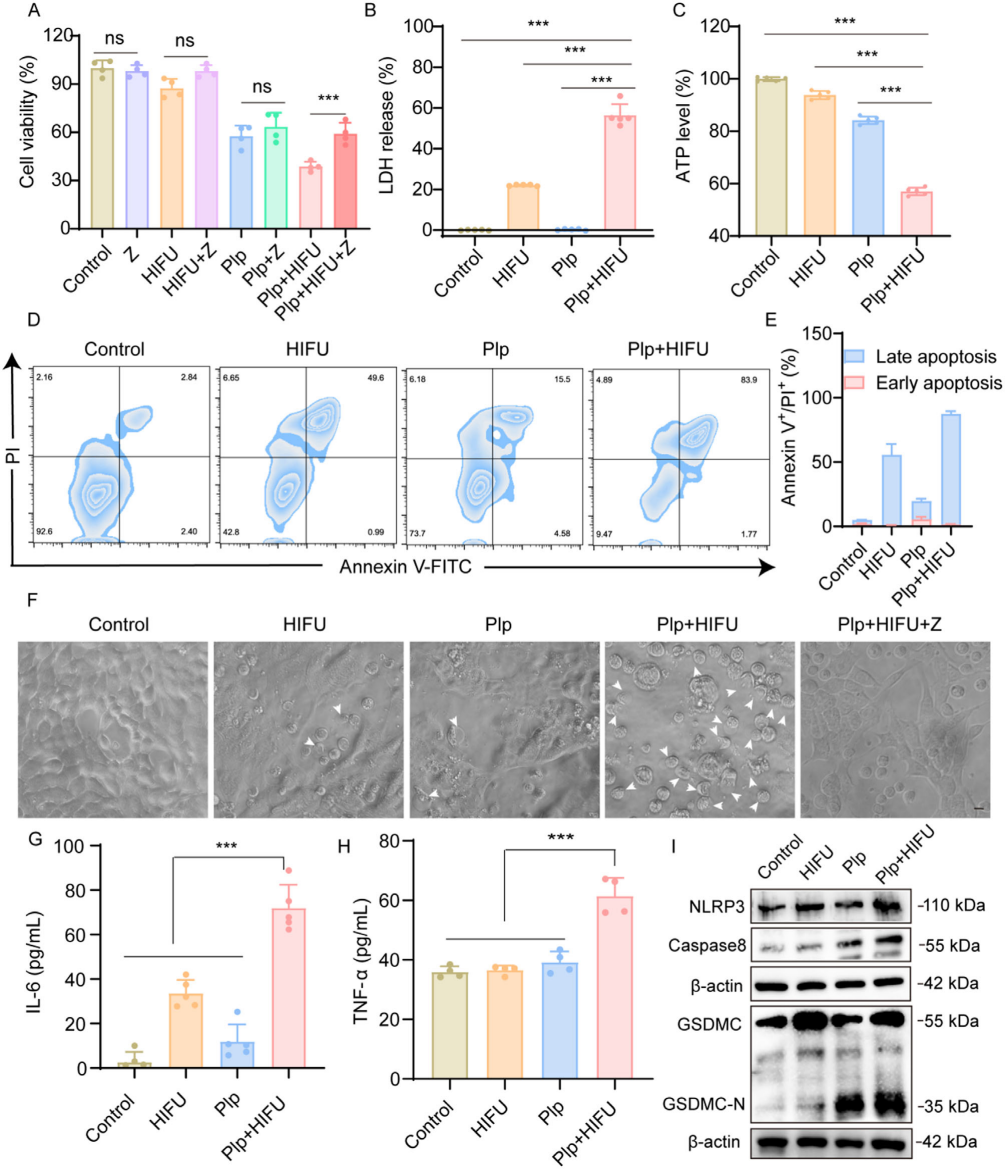
Augmentation of Plp‐induced pyroptosis by HIFU in vitro. A) Cell viability after treatment of 4T1 with different formulations (n = 4). B) LDH release from 4T1 cells after different treatments (n = 5). C) Measurement of intracellular ATP content after treatment with different formulations (n = 5). D) Flow cytometry and corresponding semi‐quantitative analyses (E) of propidium iodide and Annexin V‐FITC‐stained 4T1 cells under different treatments (n = 3). F) The Phase contrast microscopy images of 4T1 cell morphology after different formulation treatments. White arrows point to scorched cells (scale bar = 20 µm). G) Release of inflammatory factors IL‐6 from 4T1 cells after treatment with different formulations (n = 5). H) Secretion of TNF‐α inflammatory factor after co‐culture of 4T1 cells and BMDC (n = 4). I) Western blot analysis of pyroptosis‐related proteins (NLRP3, Caspase8, GSDMC‐N) after treatment in different groups. Data are presented as mean ± SD. Statistical significance was defined as ^***^ *p* < 0.001; “ns” indicates not significant.

Cell pyroptosis is a regulated form of cell death mediated by gasdermin proteins, which create membrane pores. Research has established that pyroptosis, a pro‐inflammatory form of programmed cell death, is mediated by the activation of various caspase family members, including caspase‐1, caspase‐3, caspase‐4, caspase‐5, caspase‐8, and caspase‐11.^[^ ^13^, ^42^ ^]^ Considering the pivotal role of caspases in pyroptosis, we investigated whether the anti‐tumor effects of Plp in combination with HIFU were associated with caspase‐dependent pyroptosis. To explore this, we utilized Z‐VAD‐FMK (Z), a cell‐permeable pan‐caspase inhibitor that prevents caspase‐mediated cell death. As illustrated in **Figure** **4**A, the addition of Z significantly reversed the cell death induced by Plp+HIFU combination, confirming the involvement of caspase‐dependent pathways. Furthermore, we examined the morphological changes in 4T1 cells following Plp or Plp+HIFU treatment (Figure 4F). Cells exposed to Plp+HIFU exhibited pronounced swelling and the formation of numerous spherical bubbles, hallmarks of pyroptosis. In contrast, only minor swelling and limited bubble formation were observed in cells treated with Plp or HIFU alone. Flow cytometry analysis (Figure 4D,E) confirmed that Plp+HIFU treatment significantly increased the proportion of propidium iodide (PI)‐positive cells, indicative of compromised membrane integrity resulting from pyroptosis. Additionally, we assessed the release of lactate dehydrogenase (LDH) and the intracellular concentration of ATP, both of which are indicators of pyroptosis activity. The Plp+HIFU group exhibited a substantial increase in LDH release and a significant reduction in intracellular ATP levels compared to the Plp and HIFU groups (Figure 4B,C). To evaluate the inflammatory response associated with pyroptosis, we measured the levels of IL‐6 in the cell supernatant. Notably, cells treated with Plp+HIFU showed significantly increased secretion of IL‐6 compared to other treatment groups (Figure 4G,H). Furthermore, we quantified TNF‐α secretion in co‐cultures of 4T1 cells with BMDCs. The Plp+HIFU combination treatment significantly enhanced TNF‐α production by bone marrow‐derived dendritic cells (BMDCs) compared to control groups, with increases of 1.71 fold relative to PBS control, 1.57 fold relative to Plp alone, and 1.69 fold relative to HIFU alone (Figure 4H). A similar synergistic enhancement was observed for IFN‐γ secretion, demonstrating that combined HIFU and Plp treatment robustly activates dendritic cell‐mediated immune responses (Figure S15, Supporting Information). Finally, western blot experiments were conducted to assess the impact of HIFU and Plp on pyroptosis‐related proteins. The results indicated that the combination of HIFU and Plp significantly increased the expression of NLRP3, Caspase‐8, and GSDMC in 4T1 cells compared to HIFU or Plp alone, suggesting a strong pyroptosis effect from the Plp+HIFU treatment (Figures 4I; Figure S16, Supporting Information). These findings underscore that the combination of Plp and HIFU induces caspase‐dependent pyroptosis, resulting in membrane disruption and the release of inflammatory mediators.

To further validate the critical role of the CTSL gene in HIFU‐induced Plp‐triggered pyroptosis, we transfected 4T1 cells with CTSL‐targeting siRNA to achieve gene knockdown. The results are presented in Figure S17A,B (Supporting Information), Western blot analysis confirmed effective CTSL knockdown, showing a significant reduction in CTSL protein levels 48 h post‐transfection with CTSL‐specific siRNA. Notably, GSDMC cleavage analysis revealed no detectable upregulation of the GSDMC‐N terminal fragment upon CTSL knockdown, suggesting that CTSL is essential for GSDMC processing during pyroptosis (Figure S17C, Supporting Information). Furthermore, morphological assessment via phase‐contrast microscopy demonstrated that HIFU+Plp treatment induced classical pyroptotic ballooning in 4T1 cells, whereas CTSL‐depleted cells failed to exhibit this phenotype (Figure S17D, Supporting Information). These results collectively provide molecular and morphological evidence that CTSL is indispensable for HIFU‐Plp‐induced pyroptosis, reinforcing the mechanistic insights proposed in our study.

### 2.5 Transcriptomic Analysis of 4T1 Cells Treated with HIFU Combined with Plp

**Figure 5:**
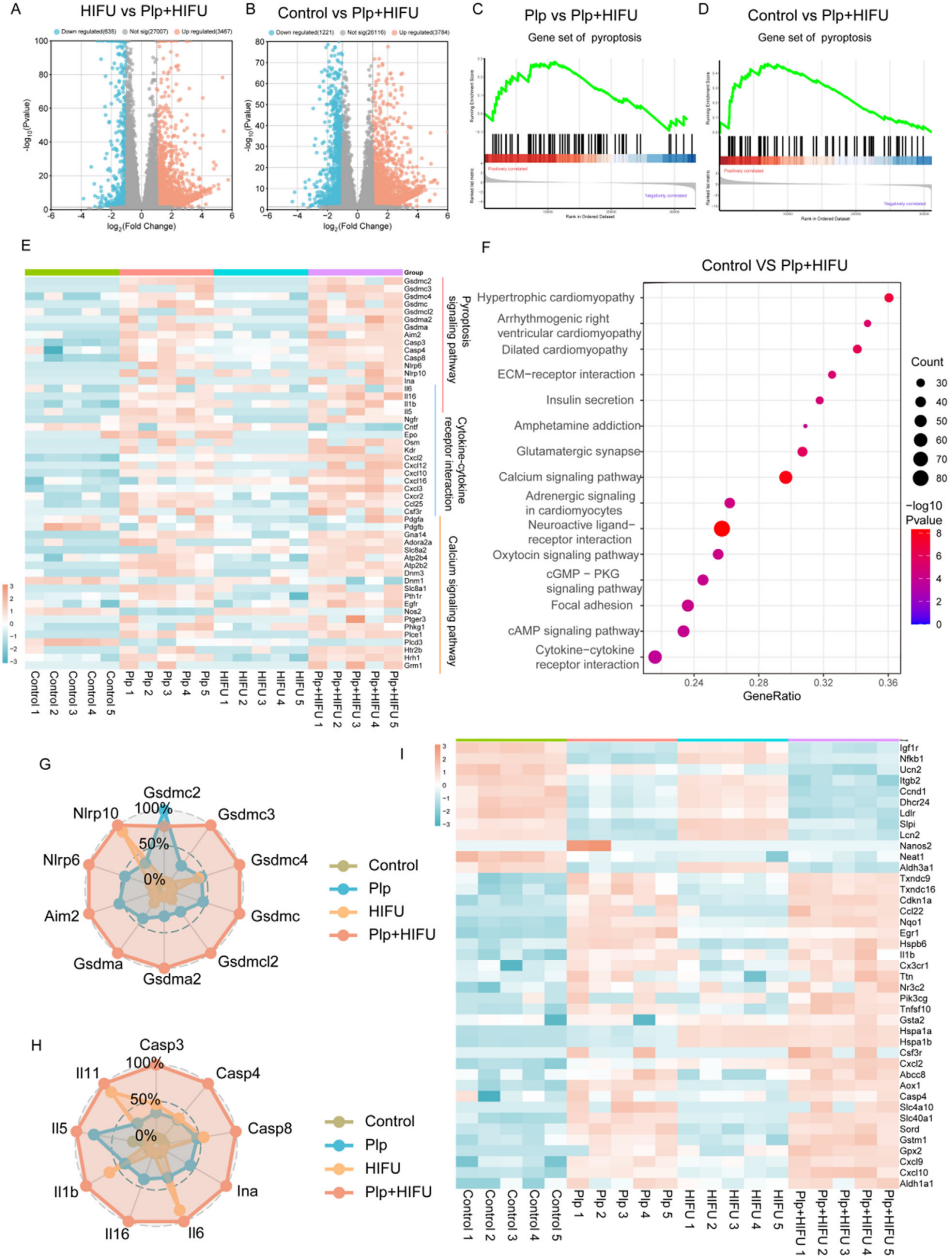
Transcriptomic analysis of 4T1 cells treated with HIFU combined with Plp. A, B) Volcano plots illustrate up‐ and down‐regulated genes in 4T1 cells across different treatment conditions (n = 5). Screening criteria were p < 0.05 and |log2 fold change| = > 1. C, D) Gene Set Enrichment Analysis (GSEA) of pyroptosis‐related genes (n = 5). E) Heatmap of differentially transcribed RNAs in 4T1 cells under various treatments (n = 5). F) KEGG pathway enrichment analysis of differentially expressed genes in 4T1 cells (n = 5). G, H) Radar plots displaying normalized expression of pyroptosis‐associated genes (n = 5). I) Heatmap of RNA transcripts for genes linked to ROS production in 4T1 cells (n = 5).

Based on the excellent anti‐tumor effects observed in vitro, we further explored the mechanism of pyroptosis induced by the combination of HIFU and Plp using transcriptomic analysis. Specifically, principal component analysis (PCA) revealed that PC1 accounted for 30.29% of the variance, capturing approximately one‐third of the total variation and effectively summarizing the primary characteristics of the data (Figure S18, Supporting Information). The Venn diagram offers a clear visualization of the shared and unique elements among groups, revealing 540, 4408, and 4876 distinct genes identified in the comparisons of Control versus HIFU, Control versus Plp, and Control versus Plp+HIFU, respectively (Figure S19, Supporting Information). This indicates that different treatment strategies significantly affect gene expression. Volcano plots visually summarize differential gene expression, highlighting up‐regulated genes in red, down‐regulated genes in blue, and genes with no significant change in gray (**Figure** **5**A,B; Figure S20, Supporting Information). The results showed that compared with the HIFU group, the Plp+HIFU group had 635 down regulated and 3467 up regulated genes, while compared with the control, the Plp+HIFU group had 1221 down regulated and 3784 up regulated genes, confirming substantial gene expression modifications in response to treatment (Figure 5A,B). Gene Ontology (GO) analysis further elucidated the biological processes (BP), cellular components (CC), and molecular functions (MF) affected by the Plp+HIFU treatment. The enrichment scores (ES) highlighted how different genes predominantly affect membrane potential regulation, receptor complex formation, and ion channel activity (Figure S21, Supporting Information). Additionally, the cent plot of MF enrichment analysis further illustrated the functions or pathways enriched with specific genes, showing a large proportion clustered in channel activation‐related pathways (Figure S22, Supporting Information).

Gene Set Enrichment Analysis (GSEA) was performed to assess the enrichment of pyroptosis‐related genes in each treatment group (Figure 5C,D; Figure S23, Supporting Information). The results showed an enrichment score of 0.28 for Plp compared to Plp+HIFU, suggesting that HIFU may amplify the pyroptosis‐inducing effect of Plp (Figure 5C). Furthermore, compared to the Control, Plp+HIFU exhibited an enrichment score of 0.46, with 36% of genes identified as core contributors forming the essential gene set (Figure 5D). Furthermore, the bubble plot from KEGG enrichment analysis demonstrated that Plp+HIFU treatment activated several relevant signaling pathways, including cytokine‐cytokine receptor interactions and calcium signaling, indicating their potential role in mediating pyroptosis. Particularly, cytokine‐cytokine receptor interactions might amplify immune and inflammatory responses, enhancing pyroptosis in tumor cells (Figure 5F). For example, cytokines, like TNF‐α, activate the NF‐κB signaling pathway through their receptors, facilitating inflammasome assembly and activation.^[^ ^43^ ^]^ The calcium signaling may influence cellular processes by modulating intracellular Ca^2^⁺ levels, offering multi‐targeted opportunities for BLBC therapy. Research suggests that intracellular Ca^2^⁺ dynamics are critical for mitochondrial function, with disruptions leading to cytochrome C release, caspase‐3 activation, and pyroptosis.^[^ ^44^, ^45^ ^]^ Additionally, IP3 receptor type 2 (IP3R2) promotes NLRP3‐mediated pyroptosis by regulating Ca^2^⁺ release from the endoplasmic reticulum. The interplay between IP3R2 and ER stress further enhances lipopolysaccharide (LPS)‐induced pyroptosis in cardiomyocytes.^[^ ^46^ ^]^ Moreover, a team has developed a Ca^2^⁺ nanomodulator (CaNM) that could induce pyroptosis through mitochondrial Ca^2^⁺ overload, serving as a pyroptosis inducer in cancer immunotherapy. Constructed from calcium carbonate (CaCO_3_) and curcumin (CUR), CaNMs induce pyroptosis by releasing intracellular Ca^2^⁺, leading to mitochondrial Ca^2^⁺ accumulation, increased ROS, cytochrome C release, caspase‐3 activation, and gasdermin E (GSDME) cleavage.^[^ ^47^ ^]^ Research indicates that mitochondrial Ca^2^⁺ accumulation activates the TCA cycle, enhancing NADH and FADH_2_ generation, which serve as key donors for the electron transport chain.^[^ ^48^, ^49^, ^50^, ^51^ ^]^ This elevated electron transport activity promotes ROS production, particularly due to electron leakage. Elevated mitochondrial Ca^2^⁺ levels impose a metabolic burden on the respiratory chain, resulting in excessive ROS generation.^[^ ^52^, ^53^ ^]^ This, in turn, compromises mitochondrial integrity, creating a feedback loop that exacerbates ROS production. The heatmap in Figure 5E visually displays expression differences in pyroptosis pathway genes and related pathways across Control, Plp, HIFU, and HIFU+Plp groups. Specifically, 18 pyroptosis‐related genes and additional genes in cytokine‐cytokine receptor interactions and calcium signaling pathways (up‐regulated by 16 and 18 genes, respectively) were highlighted. Further analysis of the interaction networks of these genes revealed distinct regulatory patterns and complex interconnections, offering valuable insights for identifying potential therapeutic targets (Figure S24, Supporting Information). It was noteworthy that the bubble chart derived from GO biological process enrichment analysis demonstrated that these genes had the highest enrichment score (3.75) in the pyroptosis signaling pathway, while the inflammatory response pathway involved the greatest number of genes (Figure S25, Supporting Information). These findings suggested that Plp combined with HIFU therapy primarily exerted its therapeutic effects by inducing the pyroptosis signaling pathway, which likely represented a key mechanism underlying its efficacy. Finally, a radar chart was used to illustrate that Plp+HIFU treatment significantly upregulated the expression of genes associated with pyroptosis and inflammation pathways. This upregulation may be attributed to the fact that combined treatment with HIFU and Plp enhances oxidative stress and inflammatory responses, thereby amplifying the pyroptosis and inflammatory signaling pathways. Notably, compared to the control, HIFU+Plp treatment resulted in a 2.34, 2.08, and 3.46 fold increase in the expression of IL‐6, GSDMC, and AIM2, respectively (Figure 5G,H).

Based on the results of these analyses, we propose that HIFU combined with Plp disrupts the mitochondrial respiratory chain through a ROS‐mediated mechanism, thereby triggering the onset of pyroptosis. Further analysis of ROS‐related gene expression revealed that 12 genes were downregulated and 29 were upregulated, highlighting substantial alterations in ROS‐associated gene profiles (Figure 5I). These findings corroborate the observed ROS production presented in Figure 3B, emphasizing the pivotal role of ROS in mediating pyroptosis. Our results not only enhance our understanding of the therapeutic mechanism of HIFU+Plp but also position ROS as a key mediator of pyroptosis, providing valuable insights for future research into the molecular mechanisms underlying this process.

### 2.6 Pharmacokinetic and Tissue Distribution Investigations

To highlight the distinct benefits of Plp, red blood cell (Rlp) and white blood cell (Wlp) membrane‐hybridized liposomes were selected as control groups for a systematic comparison. In vivo biodistribution studies (Figure S26A,B, Supporting Information) revealed that Plp exhibited a time‐dependent increase in fluorescence accumulation at the tumor site, peaking at 24 h post‐injection before gradually declining. At the 24‐h mark, the tumor accumulation of Plp was 2.40 and 2.41 fold higher than that of the Rlp and Wlp groups, respectively, indicating superior tumor‐targeting efficiency. Furthermore, Plp demonstrated a notable synergistic effect when combined with HIFU treatment. Specifically, the fluorescence intensity in the combination group was 1.59 times greater than that observed with Plp monotherapy (Figure S26C, Supporting Information). This synergistic enhancement is likely attributable to two mechanisms: the cavitation effect and mechanical shear forces induced by HIFU may disrupt tumor vascular endothelial tight junctions, thereby increasing vascular permeability;^[^ ^54^, ^55^ ^]^ and the inflammatory microenvironment triggered by HIFU may further augment the platelet membrane's specific targeting capability.^[^ ^56^ ^]^

**Figure 6:**
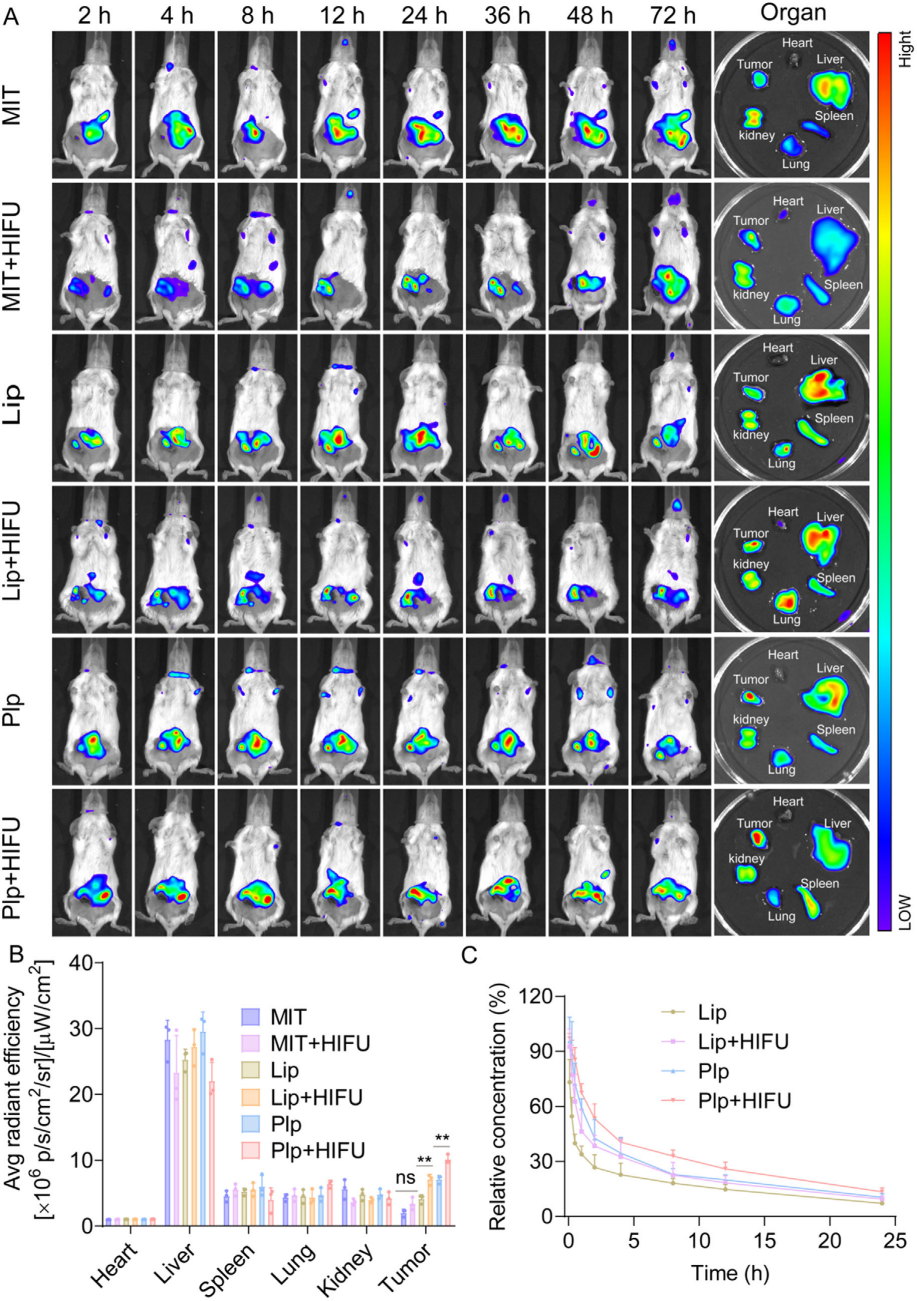
Pharmacokinetic and tissue distribution of Plp combined with HIFU. A) Fluorescence imaging of 4T1 tumor‐bearing mice at various time points post‐administration (left) and ex vivo fluorescence imaging of major organs 24 h post‐administration (right). B) Semi‐quantitative analysis of fluorescence intensity in major organs (n = 3). C) The pharmacokinetics of Plp and Lip injection at different time points in vivo (n = 4). Data are presented as mean ± SD. Statistical significance was defined as ^**^ *p* < 0.01; “ns” indicates not significant.

Building on the excellent in vitro anti‐tumor properties of Plp+HIFU, we further investigated their behavior in vivo using 4T1 tumor‐bearing mice. Spectral analysis revealed that MIT has maximal excitation and emission wavelengths of 610 and 675 nm, respectively.^[^ ^57^ ^]^ Accordingly, we used MIT's intrinsic fluorescence to monitor the tumor accumulation of Plp, Lip, and free MIT by fluorescence imaging. As shown in **Figure** **6**A, the fluorescence intensity at the tumor site was significantly higher in the Plp group compared to the Lip group after 24 h intravenous administration, suggesting that platelet targeting facilitated drug accumulation at the tumor site over time. After 24 h, fluorescence imaging of isolated vital organs revealed that the fluorescence intensity in the Plp group was 1.55 times higher than in the Lip group, likely due to platelet membrane fusion targeting tumors via CD62P‐CD44 interactions.^[^ ^32^, ^34^ ^]^ Notably, tumor fluorescence in the Plp + HIFU group was 1.59 fold higher than in the Plp group, suggesting that the thermal and shear effects of HIFU increase vascular and tissue permeability and thereby enhance drug penetration (Figure 6B).^[^ ^58^, ^59^ ^]^ By contrast, fluorescence intensities did not differ significantly between the HIFU + MIT and MIT groups, presumably because free MIT rapidly diffuses through vascular gaps and is quickly cleared, so the transient HIFU‐induced permeability does not appreciably extend its retention. After HIFU treatment, the fluorescence intensities of Plp and Lip were 3.53 and 2.06 fold greater, respectively, than that of free MIT, confirming that lipid encapsulation combined with HIFU synergistically enhances MIT accumulation at tumor sites. We subsequently homogenised tissues and quantitatively measured fluorescence in major organs and tumors. Tumor fluorescence in the Plp group was 2.03 fold higher than in the Lip group, and that in the Plp + HIFU group was 1.56 fold higher than in the Lip + HIFU group, further demonstrating superior Plp‐mediated tumor targeting in vivo (Figure S27, Supporting Information). Furthermore, we analyzed the pharmacokinetics of the Plp, revealing that the blood concentration of Plp was consistently higher than that of Lip at all time points. At 8 h post‐injection, the blood concentration in the Plp group was 33%, compared to 18.10% in the Lip group, a difference of ≈1.82 fold, indicating that Plp has a longer circulation time than Lip. Remarkably, the blood drug concentration in the Plp+HIFU group was 1.44 times higher than that in the Plp group, while the concentration in the Lip+HIFU group was 1.24 times higher than that in the Lip group at 8 h (Figure 6C). This suggests that HIFU prolongs the drug's circulation time, potentially by enhancing targeting to the tumor site and reducing drug distribution to non‐target tissues, thereby decreasing systemic metabolism or excretion.^[^ ^23^, ^60^ ^]^ This effect may indirectly increase the local concentration of the drug, enhancing its efficacy in the circulatory system for an extended period.

### 2.7 HIFU Improves the In Vivo Anti‐Tumor Efficacy of Plp

**Figure 7:**
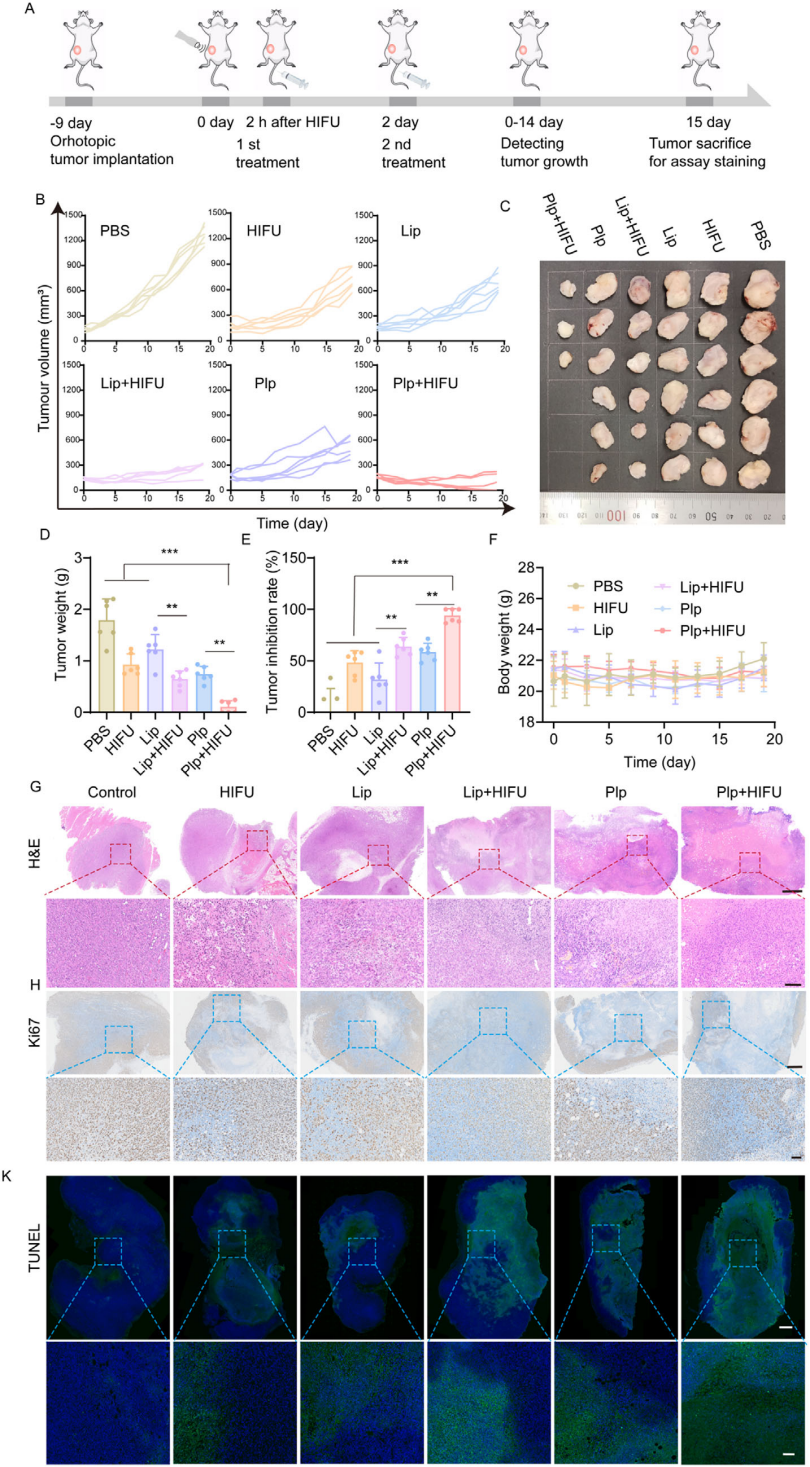
Anti‐tumor efficacy of HIFU combined with Plp in vivo. A) Schematic diagram of the treatment protocol for the 4T1 tumor model. B) Tumor volume change curves after different treatments (n = 6). C) Photographs of tumor isolates from mice after 15 days (n = 6). D) Weights of mice tumor dissociations after 15 days (n = 6). E) Tumor inhibition rate after treatment in each group (n = 6). F) Body weight changes of tumor‐bearing mice during treatment (n = 6). G) H&E staining and Ki67 staining (H) after treatment in different groups (Scale bar for the upper panel = 1 mm, and scale bar for the lower panel = 100 µm). Data are presented as mean ± SD. Statistical significance was defined as ^**^ *p* < 0.01, ^***^ *p* < 0.001; “ns” indicates not significant.

Encouraged by the results of the aforesaid experiments, we proceeded to evaluate the therapeutic efficacy of Plp in the in situ 4T1 tumor model. When the tumor volume reached ≈100 mm^3^, the mice were randomly divided into six groups and subjected to different treatments: PBS, HIFU, Lip, Lip+HIFU, Plp, and Plp+HIFU. As outlined in the treatment schedule, tumors were exposed to HIFU on day 0, followed by intravenous administration of the respective formulations at 2 h post‐HIFU and again the following day (**Figure** **7**A). Mice's body weight and tumor volume were measured every other day. After 15 days, the tumors were excised, photographed, and weighed. As shown in Figure 7B and Figure S28 (Supporting Information), HIFU treatment alone led to tumor recurrence by day 5, achieving a tumor inhibition rate of 48.18% (Figure 7E). The temporary thermal ablation effect of HIFU partially destroyed the tumor tissue but was insufficient for complete eradication. In comparison, the Lip+HIFU group exhibited a tumor inhibition rate of 63.93%, outperforming the 31.78% inhibition observed with Lip alone. This suggests that HIFU improves the antitumor effect of Lip, though the combination remained inadequate for complete tumor elimination. Remarkably, the Plp+HIFU group demonstrated a significantly higher tumor inhibition rate of 94.04%, with 50% of the mice achieving complete tumor suppression without recurrence (Figure 7E). This potent effect could be attributed to the thermal and mechanical stress induced by HIFU, which disrupts tumor density, enhances local inflammation, and facilitates the more effective delivery of platelet‐membrane‐coated liposomes to the tumor site, resulting in superior anti‐tumor activity. Tumor size was visually represented through tumor weights and photographs in Figure 7C,D. Survival analysis demonstrated a significantly prolonged median survival time in the Plp+HIFU combination group (54.5 days) compared to either Plp monotherapy (34.5 days) or HIFU monotherapy (35 days). These results suggest that the combined treatment regimen enhances therapeutic efficacy and significantly improves survival outcomes in mice (Figure S29, Supporting Information).

To further validate the therapeutic effects, the excised tumors were analyzed using hematoxylin and eosin (H&E) staining, terminal deoxynucleotidyl transferase dUTP nick end labeling (TUNEL), and Ki67 staining to assess histopathological damage and tumor cell proliferation. As shown in Figure 7G and Figure S30 (Supporting Information), H&E and TUNEL results revealed nuclear disruption revealed nuclear disruption in areas treated with HIFU, whereas treatment with Plp or Lip alone resulted in only mild tumor damage. Notably, Plp combined with HIFU caused the most extensive damage to the tumor cross‐sections, characterized by evident nuclear condensation and sparse cellular arrangement. Ki67, a marker of tumor cell proliferation, is inversely correlated with tumor inhibition, with higher expression indicating greater proliferative capacity. Immunohistochemical analysis of tumor tissues demonstrated a marked reduction in Ki67 expression in the Plp+HIFU group, indicating a significant reduction in tumor cell proliferation post‐treatment (Figure 7H). Importantly, no significant weight loss was observed in the mice during the treatment period, suggesting that Plp+HIFU did not induce noticeable systemic toxicity (Figure 7D). Post‐treatment levels of alanine aminotransferase (ALT), aspartate aminotransferase (AST), blood urea nitrogen (BUN), and creatinine (CR) were within the normal range, with no signs of hepatic or renal toxicity, confirming the safety of the Plp formulation (Figure S31, Supporting Information). Furthermore, histopathological analysis of major organs after 15 days of treatment revealed no evidence of tissue damage or inflammatory responses (Figure S32, Supporting Information). These findings underscore the remarkable therapeutic efficacy of the Plp+HIFU treatment. This efficacy is partly attributed to the fact that HIFU amplifies the effect of Plp‐induced tumor cell pyroptosis and improves the therapeutic efficacy of Plp. Additionally, the platelet‐membrane‐coated liposomes significantly enhanced drug delivery and immune evasion, contributing to the overall effectiveness of the treatment.

Considering the critical role of caspase‐8 in inducing pyroptosis in 4T1 tumor cells following treatment with Plp in combination with HIFU, immunohistochemical analysis was performed on 4T1 tumor‐bearing mice on day 7 post‐treatment to evaluate caspase‐8 expression across different groups. As shown in Figure S33 (Supporting Information), caspase8 expression was strongest in the group treated with the combination of Plp and HIFU, indicating a stronger potential therapeutic effect of the combined treatment in inducing pyroptosis. Additionally, the expression levels of HDAC4 and H3K27Ac were further investigated. Consistent with the in vitro findings, HIFU treatment significantly reduced HDAC4 expression, while Plp treatment alone did not result in any notable changes (Figure S34, Supporting Information). Previous studies have shown that hifu‐induced downregulation of HDAC4 resulted in elevated histone H3K27 acetylation.^[^ ^61^ ^]^ Immunohistochemical analysis confirmed that H3K27Ac expression was significantly elevated in the combination of Plp and HIFU treatment compared with the HIFU group, further supporting the potential mechanism of HIFU‐enhanced Plp treatment (Figure S35, Supporting Information). Collectively, these experimental results highlight the significant therapeutic potential of combining HIFU with Plp in inducing pyroptosis and enhancing treatment efficacy in 4T1 tumor cells.

To further evaluate the pyroptotic effects of HIFU combined with Plp in vivo, we performed immunoblotting analysis on tumor tissues following treatment across different experimental groups. The experimental results (Figure S36A, Supporting Information) demonstrated that compared to the HIFU or Plp monotherapy groups, the HIFU+Plp combination group exhibited significantly elevated expression levels of GSDMC‐N, a key executor of pyroptosis, confirming the critical role of GSDMC protein activation in treatment‐induced pyroptosis. Semi‐quantitative analysis revealed that GSDMC‐N expression levels in the combination group were 1.52 and 2.42 fold higher than those in the Plp and HIFU groups, respectively, indicating that the combined therapy more effectively induces pyroptosis (Figure S36B, Supporting Information). Concurrently, pyroptosis‐related molecules such as Caspase8 and NLRP3 showed similar expression trends, consistent with the in vitro experimental data. Further analysis of inflammatory factors in the tumor microenvironment (Figure S36C,D, Supporting Information) revealed that compared to the control group, the HIFU monotherapy group displayed significantly upregulated expression of pro‐inflammatory cytokines such as IL‐6 and IFN‐γ, suggesting that HIFU effectively induces local inflammatory responses in tumors. This may create a favorable microenvironment for the tumor‐targeting capability of Plp. Notably, the HIFU+Plp combination group exhibited a more pronounced increase in these inflammatory factors compared to the monotherapy groups, correlating with the tumor growth inhibition results. Collectively, these data demonstrate that activating the “pyroptosis‐inflammation” cascade to remodel the tumor microenvironment is a key mechanism underlying the synergistic antitumor effect of this combination therapy.

### 2.8 HIFU Enhances the Anti‐Tumor Immune Response of Plp

**Figure 8:**
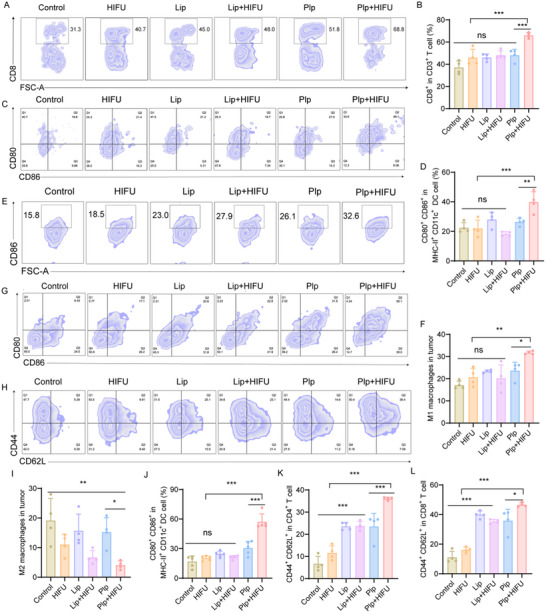
HIFU enhances the anti‐tumor immune response of Plp. (A–D) Representative flow cytometry plots and corresponding quantitative analyses of CD8⁺ T cells (gated on CD3⁺ cells) and CD80⁺CD86⁺ dendritic cells (gated on MHC II⁺CD11c⁺ cells) within tumor tissues (n = 4). (E–F) Flow cytometric analysis and semi‐quantitative assessment of M1 macrophages in tumors (n = 4). (G–J) Representative flow cytometry plots and quantification of CD80⁺and CD86⁺ DCs gated on MHC II⁺CD11c⁺ cells in draining lymph nodes (n = 4). (H–K) Representative plots and quantitative analysis of CD44⁺CD62L⁺ memory T cells gated on CD4⁺ T cells in the spleen (n = 4). (I) Representative flow cytometry plot and quantification of M2 macrophages in tumors (n = 4). (L) Quantitative analysis of CD44⁺CD62L⁺ memory T cells gated on CD8⁺ T cells in the spleen (n = 4). Data are presented as mean ± SD. Statistical significance was defined as ^*^ *p* < 0.05, ^**^ *p* < 0.01, ^***^ *p* < 0.001; “ns” indicates not significant.

Previous studies have demonstrated that during pyroptosis, Gasdermin‐mediated pore formation leads to membrane rupture and the release of large quantities of intracellular contents. These danger‐associated molecular patterns (DAMPs) can be recognized by dendritic cells (DCs), macrophages, and other immune cells, promoting their maturation and enhancing antigen presentation.^[^ ^62^ ^]^ These findings prompted us to further explore the in vivo antitumor immune potential of this combination therapy. When tumor volumes reached ≈200 mm^3^, mice were randomly assigned to six groups and received various treatments. On day 5 post‐treatment, tumors, tumor‐draining lymph nodes (TDLNs), and spleens were harvested for immune cell profiling. The results showed that in the Plp+HIFU group, ≈39.88% of tumor‐infiltrating DCs expressed high levels of co‐stimulatory molecules CD80^+^ and CD86^+^—representing a 1.78 fold increase compared to the untreated control group (**Figure** **8**C,D). Furthermore, as shown in Figure 8G,J, DCs from lymph nodes also exhibited significantly higher maturation levels following Plp+HIFU treatment, with increases of 1.87 and 2.82 fold compared to the Plp‐only and HIFU‐only groups, respectively. These findings suggest that pyroptosis induced by Plp+HIFU not only enhances DC maturation within the TME but also exerts immunostimulatory effects on secondary lymphoid organs, potentially facilitating systemic antitumor immunity. The upregulation of CD80^+^ and CD86^+^ indicates enhanced antigen‐presenting capacity and T cell priming, which are critical steps in the initiation and amplification of adaptive immune responses.^[^ ^63^ ^]^ We further examined the intratumoral immune landscape, focusing on CD8⁺ T cells as key effectors of adaptive antitumor immunity. Flow cytometric analysis revealed a significant increase in CD8⁺ T cell populations within CD3⁺ T cells (Figure 8A,B), with a 1.78 fold increase compared to the control group. These results suggest that Plp+HIFU‐induced pyroptosis promotes DC maturation, facilitates effective antigen presentation, and subsequently activates T cells, leading to their clonal expansion and functional differentiation. Moreover, analysis of tumor‐associated macrophages revealed a shift toward a pro‐inflammatory phenotype: the proportion of M1 macrophages increased from 17.13% (PBS group) to 31.7% (Figure 8E,F), while the proportion of immunosuppressive M2 macrophages decreased from 19.13% to 4.06% (Figure 8J; Figure S37A, Supporting Information). This phenotypic shift indicates that Plp+HIFU not only activates innate immune responses but also reshapes the TME into an immunologically active state conducive to tumor eradication. Importantly, systemic immune activation was also evident in the spleen. The proportions of CD44⁺CD62L⁺ central memory T cells within CD4⁺ and CD8⁺ T cell subsets increased significantly to 36.05% and 46.63%, respectively, following Plp+HIFU treatment—markedly higher than those in the PBS and Plp‐alone groups (Figure 8H,K,L; Figure S38B, Supporting Information). These findings suggest that the combination therapy enhances the formation of central memory T cells, potentially contributing to long‐term immune surveillance and durable antitumor responses. In summary, Plp+HIFU therapy promotes immune cell activation, enhances both local and systemic antitumor immunity, and reprograms the TME toward a pro‐inflammatory, immune‐activated state. These effects collectively lay the foundation for more effective and durable antitumor therapeutic strategies.

## 3 Conclusion

In summary, we have developed a HIFU‐driven targeted pyroptosis strategy for the treatment of BLBC. This strategy integrates HIFU‐mediated gene regulation, bioinformatics, multi‐database drug screening, and experimental validation to facilitate the discovery and optimization of chemotherapeutic drugs. Initially, a bioinformatics approach was employed to identify pyroptosis‐related genes by comparing normal and BLBC samples in the TCGA dataset, and their relevance to BLBC was assessed through survival analysis. Subsequently, mRNA sequencing was used to analyze changes in the expression of pyroptosis‐related genes following HIFU treatment, which led to the identification of 20 potential pyroptosis inducers based on gene‐drug interaction data. Based on the characteristic effects of the drugs themselves, we selected 9 drugs for initial validation and identified one of the most promising drugs, MIT, encapsulated in platelet membrane heterozygous liposomes. Finally, we explored the potential mechanisms underlying the combined action of HIFU and Plp in inducing pyroptosis. On one hand, HIFU irradiation induced a significant inflammatory response at the tumor site through mechanical stress, which enhanced the accumulation of Plp in the tumor. On the other hand, HIFU treatment reduced the expression of HDAC4 and HDAC9, increased the acetylation of histone H3K27, and promoted the transcription of the CTSL gene. Simultaneously, Plp and HIFU synergistically inhibited BCL‐2 expression through CTSL, leading to increased ROS production. This, in turn, activated caspase‐8 and the NLRP3 inflammasome, resulting in the N‐terminal cleavage of GSDMC and the initiation of pyroptosis. In conclusion, this study demonstrates the potential of HIFU‐driven discovery of pyroptosis‐associated genes and drugs for BLBC treatment, providing a valuable theoretical foundation for novel therapeutic strategies. Moreover, this approach offers an innovative framework for drug development targeting other refractory diseases.

## 4 Experimental Section

### Materials

Egg yolk lecithin, cholesterol, and 1,2‐distearoyl‐sn‐glycero‐3‐phosphoethanolamine‐N‐[methoxy(polyethylene glycol)‐2000] (DSPE‐PEG2000) were purchased from AVT (Shanghai) Pharmaceutical Tech Co., Ltd. Mitoxantrone was purchased from Dalian Meilun. Panobinostat, Bortezomib, and Curcumin were purchased from Solarbio. Crizotinib, Doxorubicin hydrochloride, Celecoxib, Fulvestrant, and Artenimol were provided by Aladdin. ATP and BCA protein detection kits were purchased from Beyotime. Calcein‐AM/PI, Annexin‐V‐FITC/PI, and CCK‐8 were products of Keygen BioTECH. Caspase‐8 and NLRP3 were provided by Proteintech. HDAC4, HDAC5, and HDAC9 antibodies were provided by HUABIO. GSDMC, HDAC‐1238, and HDAC459 antibodies were provided by abcam. ELISA kits for IL‐6 (JL20268‐96T), IFN‐γ (JL10967‐96T), and CISL (JL33944‐96T) were purchased from Jianglai Bio, Shanghai. The serum was provided by Suzhou Shuangru Biotechnology Co., Ltd (LONSERA).

Animals: Female BALB/c mice, aged 4–6 weeks, were obtained from Shanghai Bikai Laboratory Animal Co., China. All animal procedures were carried out in compliance with the guidelines approved by the Ethics Committee of Fudan University (2024‐07‐YJ‐PZQ‐89).

Cells: The mouse breast cancer cell lines MDA‐MB‐231 and 4T1 were purchased from the American Type Culture Collection (ATCC, USA). Both cell lines were cultured in DMEM and RPMI‐1640 media, respectively, supplemented with 10% fetal bovine serum and 1% penicillin/streptomycin.

The detailed HIFU parameter settings in this study were specified below: a central frequency of 10 MHz, pulse repetition frequency (PRF) of 1000 Hz, duty cycle of 70%, and a focal area of 0.06 cm × 0.08 cm. The average acoustic intensity was 34 W cm^−^^2^.

### Recognizing Key Pyroptosis‐Related Genes and Probing Pyroptosis Drug Candidates

A list of 238 pyroptosis‐related genes was curated from the Gene Set Enrichment Analysis (GSEA) database (https://www.gsea‐msigdb.org/gsea/msigdb/index.jsp). These genes were then analyzed for their expression in BLBC and normal breast tissues using data obtained from The Cancer Genome Atlas (TCGA) (https://www.cancer.gov/ccg/research/genome‐sequencing/tcga). Following this, 28 key pyroptosis‐related genes were selected as potential targets for BLBC. Following this, 28 key pyroptosis‐related genes were selected as potential targets for BLBC. Survival analysis of these 28 genes was conducted based on the Akaike Information Criterion (AIC), which provides the optimal balance between model fit and simplicity. These genes were identified based on their high Akaike Information Criterion (AIC) scores, which indicate the best balance between model fit and simplicity.^[^ ^64^ ^]^ A protein‐protein interaction (PPI) network about these 28 genes was constructed based on the relationship data obtained from the STRING database (https://cn.string‐db.org/). Using these 28 key pyroptosis genes, a total 25147 potential drug candidates were identified through chemical‐gene interactions sourced from the Comparative Toxicogenomics Database (http://ctdbase.org/downloads/). To further screen these candidates, RNA expression data of pyroptosis regulators and corresponding compound activity data (DTP NCI‐60) were obtained from the CellMiner database (https://discover.nci.nih.gov/CellMiner/home.do). A correlation analysis was performed between the mean z‐scores of compound activity and the RNA expression of pyroptosis regulators. As a result, 94 drug candidates were identified that showed a significant association with at least one key gene involved in pyroptosis.

### Extraction of Platelet Membranes

Platelets were isolated through gradient centrifugation using the following procedure: anticoagulated whole blood was mixed with 1 × PBS (containing 7.5 mM EDTA) at a 1:1.5 volume ratio and centrifuged at 200 g for 15 min. The pellet was discarded, and the supernatant was transferred to a new 15 mL tube. An equal volume of 1 × PBS (containing 1 mM EDTA and 2 µM PGE1) was added, followed by gentle inversion 6–8 times to mix. The sample was then centrifuged at 100 g for 20 min at room temperature. The supernatant was transferred to a new centrifuge tube, and a second centrifugation was performed at 800 g for 25 min. The resulting pellet, containing the platelets, was gently resuspended in 1×PBS (containing 1 mM EDTA and a protease inhibitor) and stored at −80 °C.

For platelet membrane extraction, the platelets were thawed in a 37 °C water bath, then rapidly frozen in liquid nitrogen, and this freeze‐thaw cycle was repeated 8–11 times. Following the final cycle, the platelets were centrifuged at 12 000 g for 10 min at 4 °C. The supernatant, containing protease inhibitors, was discarded, and the pellet was resuspended in 1 mL of purified water. After a single wash by gentle pipetting, the supernatant was discarded, and the pellet was resuspended in PBS for storage at 4 °C.

### Synthesis and Characterization of Plp

Bionic liposomes were prepared using the film hydration‐extrusion method. The preparation procedure was as follows: 4 mg of egg yolk lecithin, 0.4 mg of DSPE‐PEG2000, 1 mg of cholesterol, and 0.5 mg of MIT were weighed. Each of the first three components was dissolved in 2 mL of dichloromethane and homogenized by ultrasonication, then transferred to a cigar‐shaped flask. MIT was dissolved in 2 mL of methanol and added to the flask, and the mixture was gently shaken to ensure thorough mixing. The dichloromethane and methanol were evaporated under reduced pressure using a rotary evaporator to form a lipid film on the flask wall. The platelet membrane, prepared from 2 mL of whole blood, was dispersed in water and added to the flask. The mixture was hydrated for 1 h at room temperature with stirring, followed by 5 min of ultrasonication in an ice‐water bath. The liposomes were then passed through 400, 200, and 100 nm polycarbonate membranes sequentially using an extruder. Liposomes were extruded 10 times through the polycarbonate membranes to obtain Plp, which was stored at 4 °C. Liposomes without platelet membranes (Lip) were prepared using the same procedure.

### Characterization of Plp, a Platelet‐Mimicking Liposome

Appropriate volumes of Lip and Plp were diluted with pure water to the required concentration. The average particle size and polydispersity coefficient (PDI) of the two preparations were assessed using dynamic light scattering (DLS), with each sample being measured three times. Zeta potential was determined using electrophoretic light scattering (ELS), also with three repetitions for each sample.

To prepare DiD‐labeled liposomes, 0.2% (v/v) DiD was added to the organic phase before evaporation. The subsequent steps were performed as previously described to obtain DiD‐MIT‐Plp and DiD‐MIT‐Lip.

Lip and Plp were dissolved in PBS and stored at 4 °C. The changes in mean particle size and PDI were observed over 7 days to assess the stability of Plp placement.

For the measurement of drug loading capacity (DLC) and encapsulation efficiency (EE), MIT‐Plp and blank Plp were subjected to ultrafiltration to separate the free MIT not loaded onto the Plp. The absorbance of the free MIT was measured at 610 nm using a UV spectrophotometer (Agilent, USA). The calculations were performed using the following formulas:

DLC (%) = (Amount of drug encapsulated in liposomes / Total weight of liposomes) × 100%

EE (%) = (Amount of drug encapsulated in liposomes / Total amount of drug) × 100%

Using these formulas, the DLC and EE of MIT in biomimetic liposomes were determined.

Cryo‐TEM was employed to observe the morphology of Plp and Lip. Initially, the carbon support film was cleaned and rendered hydrophilic using a plasma surface treatment instrument to enhance the hydrophilicity of the copper grid. Plp and Lip samples, with a lipid concentration of 2 mg mL^−1^, were prepared using a rapid cryostat. The copper grid was then rapidly frozen in liquid ethane and imaged using a 200 kV transmission electron microscope.

### HIFU Enhances the Uptake and Cytotoxicity of Plp In Vitro

Cellular uptake assay: 4T1 cells were seeded into 12‐well plates and cultured overnight. After 24 h, the cells were trypsinized, centrifuged, and resuspended in 1 mL of culture medium. The cells were then transferred to 12‐well plates and subjected to 8.4 W polymerization ultrasound for 30 seconds. Subsequently, 8 µg mL^−1^ of DiD‐MIT‐Plp and DiD‐MIT‐Lip were added to each well, respectively. The cells were incubated for 2 h, and the fluorescence intensity was measured using a flow cytometer (Ex/Em = 640/670 nm). Additionally, cell nuclei were stained with DAPI and observed under a confocal laser scanning microscope (CLSM) (FV31S; Olympus, Japan).

Cell proliferation assay: To investigate the enhancing effect of high‐intensity focused ultrasound (HIFU) on liposome treatment, 4T1 cells were seeded into 12‐well plates and cultured overnight. Following trypsin digestion and cell collection, the cells were treated with 8.4 W HIFU for 30 seconds. Plp and Lip were then added to the cell suspension to achieve a final MIT concentration of 0.1 µg mL^−1^. After 24 h of incubation, cell viability was assessed using the CCK‐8 assay. After the same procedure mentioned earlier, Calcein‐AM/PI was utilized to stain the viable and non‐viable cells, followed by visualization of these cells using CLSM.

### Flow Cytometry of Pyroptosis Markers in 4T1 Cells Treated with HIFU Combined with Plp

Quantitative analysis of pyroptosis: For the quantitative analysis of pyroptosis, 4T1 cells treated with various agents (Control, HIFU, Plp, Plp+HIFU) were gathered and washed thrice with PBS. Subsequently, they underwent staining using Annexin V‐FITC/PI Apoptosis Detection Kit per the provided guidelines and were analyzed using flow cytometry (CytoFLEX S, Beckman, USA).

ROS detection assay: To measure ROS production in tumor cells, 4T1 cells were exposed to various formulations for 6 h, incubated with DCFH‐DA (10 µm) for 30 min, washed thrice with PBS, and subsequently analyzed using flow cytometry. Furthermore, post the same cell treatment procedure, nuclei were stained with DAPI and visualized under CLSM.

Mitochondrial membrane potential assay experiment: JC‐1 was utilized as a probe to assess alterations in mitochondrial membrane potential. Following treatment of cells in varied experimental groups, cells were stained with 200 µL of working solution and incubated at 37 °C for 25 min. Subsequently, flow cytometry was employed for cell detection.

### Pyroptosis Observation in 4T1 Cells Treated with HIFU Combined with Plp

Observation of cell morphology: 4T1 cells were seeded into 12‐well plates and incubated overnight, and the cells were digested with trypsin, centrifuged, and resuspended in 1 mL of culture medium. The cells were then transferred to 12‐well plates and exposed to 8.4 W polymerization ultrasound for 30 s. Subsequently, different formulations were added to each well separately, and the cells were incubated with these formulations for 24 h. Cell morphology was observed using a phase contrast microscope (Olympus, Japan).

LDH and ATP emission tests: 4T1 cells were planted into 12‐well plates and incubated for 24 h, and exposed to various treatments. The levels of cellular LDH and ATP were assessed following the protocols of the Firefly Luciferase ATP Assay Kit (Beyotime Biotechnology) and LDH Cytotoxicity Assay Kit (Beyotime Biotechnology). Luminescence values and absorbance readings for each well were recorded using a microplate reader (Multiskan MK3, Thermos, USA).

Inflammatory factor release assay: 4T1 cells were seeded in 12‐well plates, cultured for 24 h, and treated with different preparations. Levels of cellular IL‐6 were evaluated according to the respective instructions. Absorbance of each well was measured using a microplate reader (Multiskan MK3, Thermos, USA).

Western blot Experiments: Cells were lysed with RIPA lysate after different treatments, and protein concentration was quantified using BCA. A Western blot was performed to detect the presence of specific protein markers. The resulting gels were transferred to PVDF membranes, and the antibodies were incubated overnight at 4 °C. The resulting bands were finally quantified using ImageJ software.

To investigate the critical role of the CTSL gene in HIFU‐induced pyroptosis in combination with Plp, siRNA‐mediated CTSL knockdown was employed. 48 h post‐transfection, CTSL knockdown efficiency was verified by immunoblotting. 24 h after transfection, cells were subjected to HIFU treatment followed by Plp administration. Subsequently, the expression of the key pyroptosis executioner protein GSDMC was analyzed by immunoblotting and monitored cellular morphological changes using phase‐contrast microscopy.

### Pharmacokinetic and Tissue Distribution Investigations

To evaluate the pharmacokinetics and tissue distribution of Plp, an in situ breast cancer model was established in BALB/c mice. Once the tumor volume reached ≈300 mm^3^, the mice were divided randomly into four groups (Lip, Lip+HIFU, Plp, Plp+HIFU) with four mice in each group. Two of these groups were irradiated with 8.4 W HIFU at the tumor site for 30 seconds, and 2 h later, 150 µL of DiD‐Plp or DiD Lip was injected intravenously. Orbital blood samples (50 µL) were collected into heparinized test tubes at various time points post‐injection. Blood samples were diluted with PBS and analyzed for DiD fluorescence intensity using a microplate reader (Ex/Em = 640/670 nm).

To study Plp accumulation in tumors over time, 16 additional tumor‐bearing mice underwent the same procedure. Drug accumulation at the tumor site was visually monitored at specific intervals using IVIS Spectrum in vivo fluorescence imaging. After 24 h post‐injection, mice were euthanized, major organs (including tumors) were harvested, and imaged ex vivo using the IVIS Spectrum in vivo fluorescence imaging system. Subsequently, organs and tumors were homogenized in PBS using a tissue grinder (BioBio, Shanghai, China). The fluorescence intensity of these homogenates was measured using a microplate reader (Ex/Em = 610/675 nm).

### Transcriptomic Analysis of 4T1 Cells Treated with HIFU Combined with Plp

4T1 cells were inoculated into 6‐well plates and incubated overnight. After that, they were exposed to different treatment groups (Control, HIFU, Plp, Plp+HIFU) for 12 h. Subsequently, cells were harvested, and the following procedures were carried out. Initially, total RNA was isolated using the Universal RNA Extraction CZ kit (RNC643, ONREW) following the manufacturer's guidelines. The RNA quantity was assessed using Quantum Bits 4.0 (Invitrogen), while RNA quality was confirmed through denaturing agarose gel electrophoresis. RNA libraries were constructed utilizing the VVAHTSUniversal V8 RNA‐seq Library Prep Kit for Illumina (NR605‐0, Vazyme) and then sequenced on the Illumina NovaSeq 6000 platform with a 150‐pair sequencing method. mRNA enrichment, library preparation, sequencing, and data analysis were all conducted by Shanghai Xuran Biotechnology Co Ltd (http://www.xurangene.com).

### Anti‐Tumor Efficacy of HIFU Combined with Plp In Vivo

To evaluate the therapeutic efficacy of the drugs in vivo, BALB/c mice were inoculated with 4T1 cells in situ. Once the tumor volume reached ≈100 mm^3^, the mice were randomly assigned to six groups, each consisting of six mice. The mice received 100 µL of Plp, Lip, or PBS following 30 seconds of 8.4 W HIFU irradiation, with treatments spaced 2 h apart. The second treatment was administered the following day. During the treatment period, body weight and tumor volume were measured bi‐daily, with tumor volume calculated using the formula: length × width^2^ / 2. After 15 days, five mice from each group were randomly selected for standard blood and biochemical tests. Additionally, vital organs were subjected to histopathological examination, and tissue sections were analyzed using TUNEL, Ki67, and H&E staining, observed under an inverted fluorescence microscope (Nikon, Japan).

On day 2 post‐treatment, tumor tissues were harvested from each group, cryopreserved, and subsequently centrifuged. The levels of inflammatory factors were then quantified using a commercial ELISA kit according to the manufacturer's protocol.

### HIFU Enhances the Anti‐Tumor Immune Response of Plp

On the third day post‐treatment, tumor tissues were harvested, and tumor‐draining lymph nodes (TDLNs) were dissected. Spleens were homogenized into single‐cell suspensions, which were then filtered through a 0.22 µm strainer. For dendritic cell (DC) maturation analysis, lymph node cells were combined with 100 µL of the single‐cell suspension and stained with the following antibodies: anti‐CD45‐BV510, anti‐CD11c‐APC, anti‐MHC‐II‐FITC, anti‐CD86‐PE, and anti‐CD80‐PE/CY7. After 30 min of incubation at room temperature in the dark, cells were washed with PBS and resuspended in 200 µL PBS. Mature DCs, defined as CD11c^+^CD86^+^ and CD11c^+^CD80^+^ populations, were quantified via flow cytometry.

For memory T cell assessment, 100 µL of splenic single‐cell suspension was stained with anti‐CD45‐BV510, anti‐CD3‐BV605, anti‐CD4‐FITC, anti‐CD8‐PE/CY7, anti‐CD44‐PE, and anti‐CD62L‐APC. Following a 30 min incubation, cells were washed and resuspended in 200 µL PBS before flow cytometric analysis. Additionally, tumor cells were processed as described above. For macrophage phenotyping, an aliquot (100 µL) was stained with anti‐CD45‐BV510, anti‐F4/80‐BV650, anti‐CD86‐PE, and anti‐CD206‐Percp/cyanine5.5 for 30 min and analyzed by flow cytometry.

### Statistical Analysis

Statistical analyses were performed using two‐way ANOVA with GraphPad Prism 8.0. All data were expressed as mean ± standard deviation (SD). ^*^ *p* < 0.05, ^**^ *p* < 0.01, and ^***^ *p* < 0.001 were statistically significant, and ns indicates no statistical significance.

## Conflict of Interest

The authors declare no conflict of interest.

## Supporting information



Supporting Information

## Data Availability

The data that support the findings of this study are available from the corresponding author upon reasonable request.
