# Supplementary material for: HIFU‐Driven Targeted Pyroptosis Therapy in Basal‐Like Breast Cancer
Source: Adv Sci (Weinh). 2025 Sep 14;12(44):e03830. doi: 10.1002/advs.202503830 (PMC12667447; doi:10.1002/advs.202503830)
Supplement: Supplementary file 1 — Supporting Information [file ADVS-12-e03830-s001.docx]

**HIFU-Driven Targeted Pyroptosis Therapy in Basal-like Breast Cancer**

***Xiaomin Su ^a,b 1^, Yang Wang ^a,b 1^, Xifeng Qin ^c^, Yaqiong Xiao ^a^, Boshu Ouyang ^d^, Lina Hu ^e^, Lin Kang ^b^, Ruizhe Xu ^c^, Ce Xu ^a^, Zanya Sun ^a^, Chenyu Sun ^a^, Huishu Guo ^a,^*^∗^, *Zhiqing Pang ^c,^*^∗^ *Shun Shen ^b,^*^∗^**

a *Central Laboratory, First Affiliated Hospital, Institute (College) of Integrative Medicine, Dalian Medical University, Dalian 116021, China*

b *Pharmacy Department, Shanghai Pudong Hospital, Fudan University Pudong Medical Center, Shanghai, 201399, China*

*c School of Pharmacy & Key Laboratory of Smart Drug Delivery, Fudan University, Shanghai 201203, China*

*d ShuGuang Hospital Affiliated to Shanghai University of Traditional Chinese Medicine, Shanghai, 201203, P. R. China.*

*e Department of Oncology, Fudan University Pudong Medical Center, Shanghai, 201339, China.*

** Corresponding author*

*1 Equal contribution to the work*


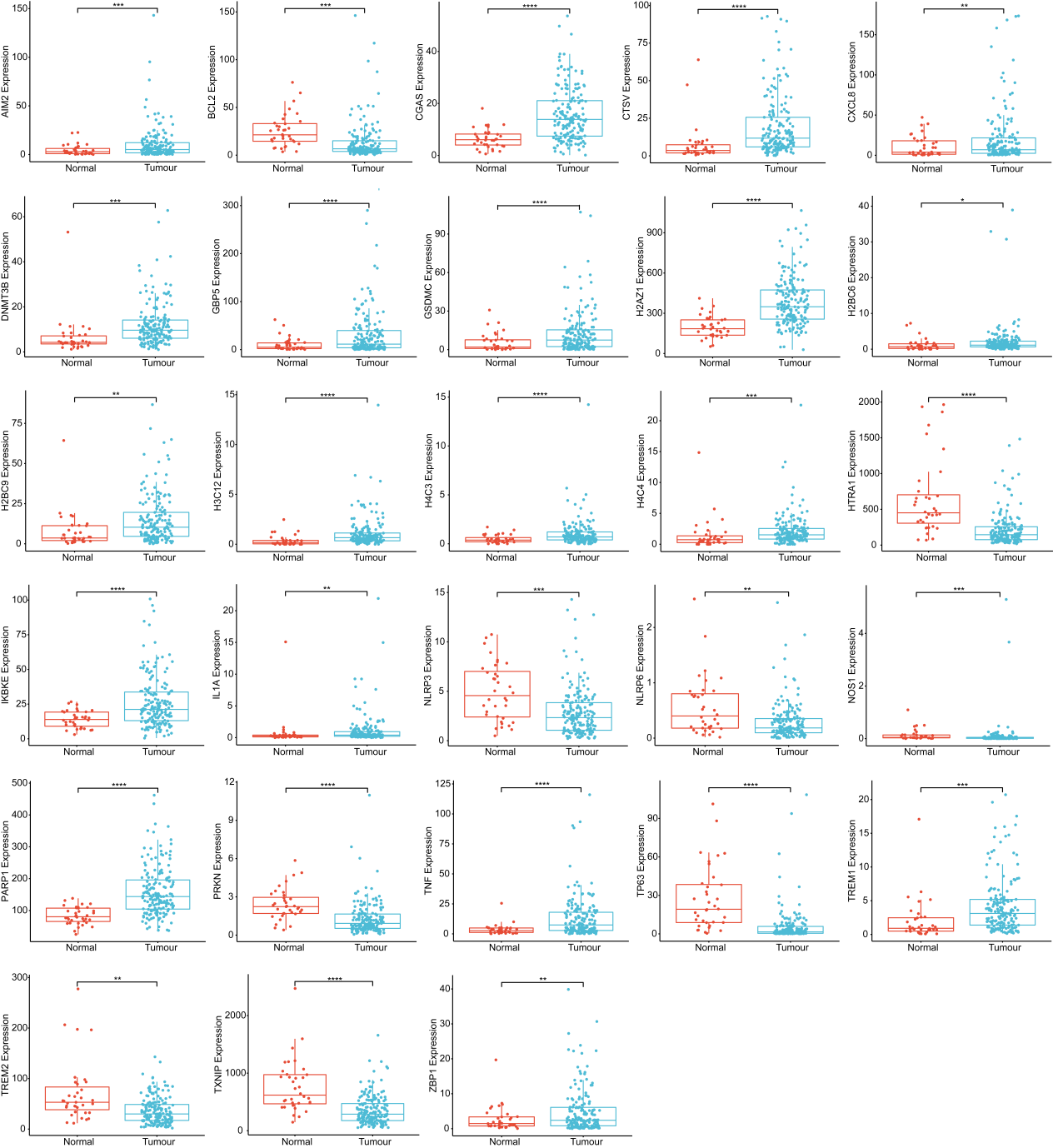


Figure S1. Box plots of the expression levels of 28 pyroptosis-related genes in the TCGA cohort in BLBC and normal breast tissue. Data are presented as mean ± SD. Statistical significance was defined as ^*^*p* < 0.05, ^**^*p* < 0.01, ^***^*p* < 0.001.


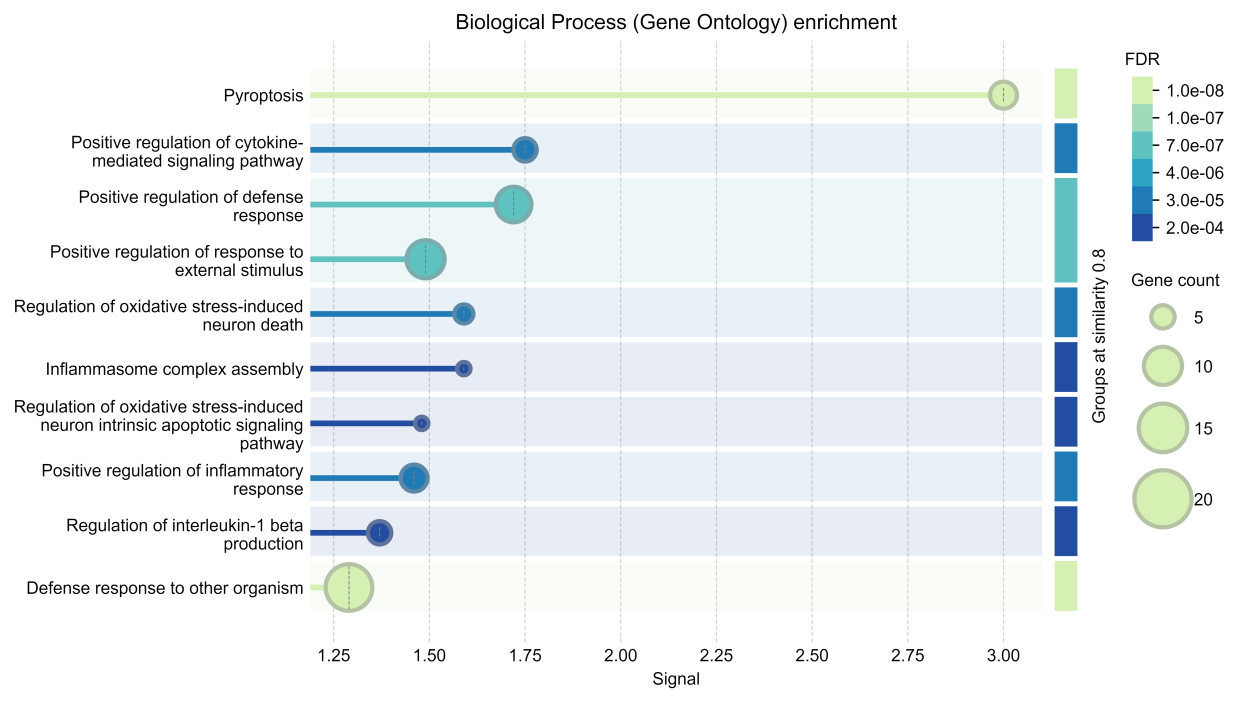


Figure S2. GO analysis of enrichment scores for 28 gene-related pathways.


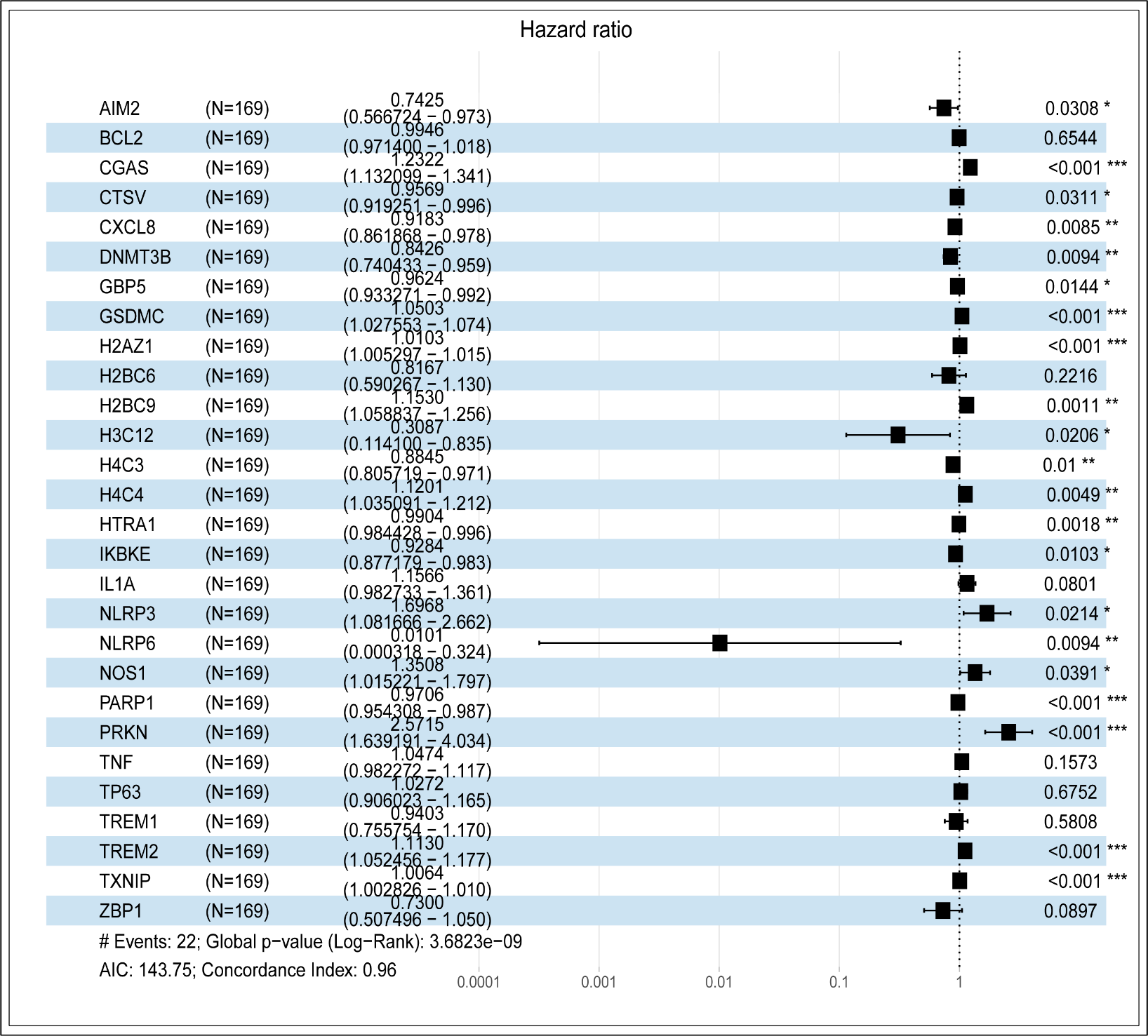


Figure S3. Forest Plot of Hazard Ratios for Gene Expression in a Survival Analysis Study (n = 169 samples).


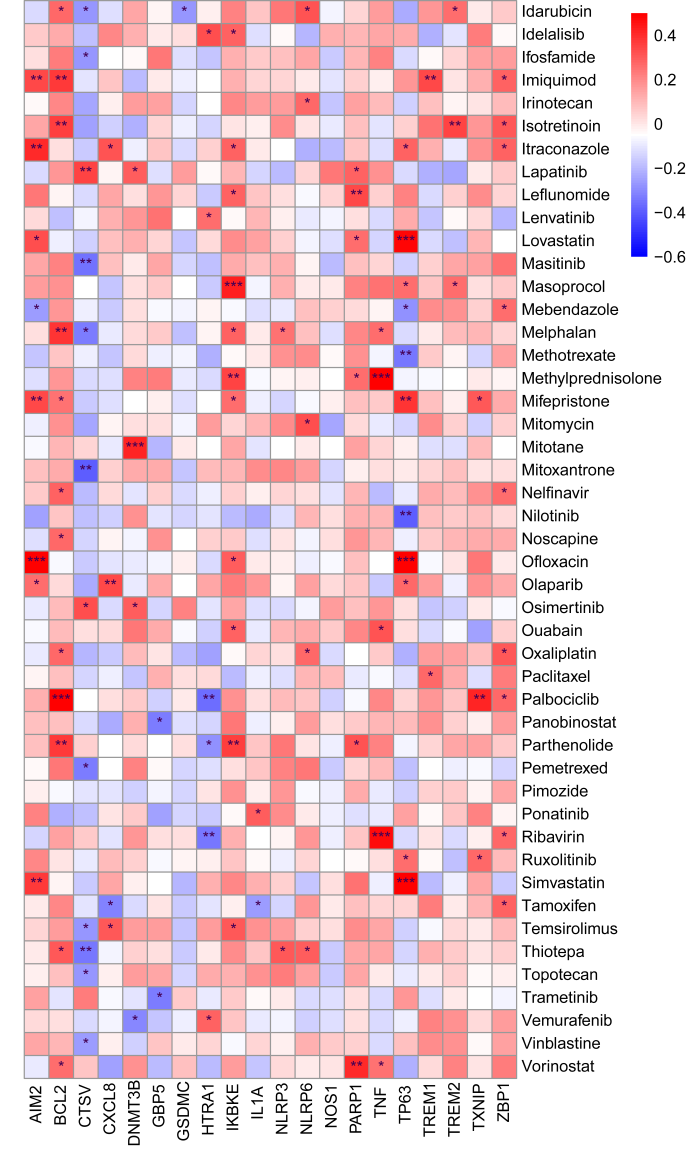


Figure S4. Correlation between IC_50_ values of 47 predicted pyroptosis-inducing agents and the expression levels of pyroptosis-regulated genes. Statistical significance was defined as ^*^*p* < 0.05, ^**^*p* < 0.01, ^***^*p* < 0.001.


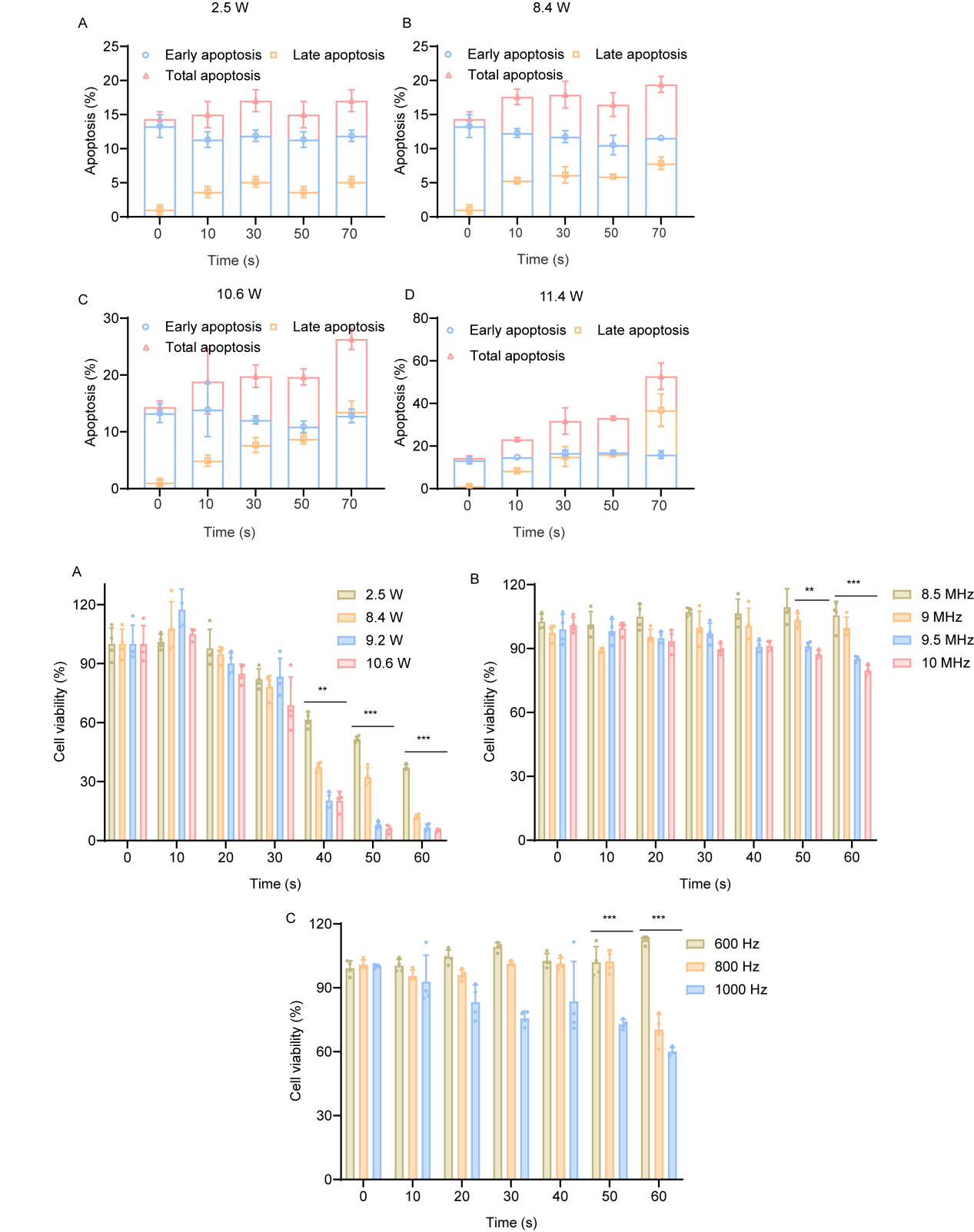


Figure S5. Cell viability of 4T1 cells under different power (A), different frequencies (B), and different pulse conditions (n=4). Data are presented as mean ± SD. Statistical significance was defined as ^**^*p* < 0.01, ^***^*p* < 0.001.


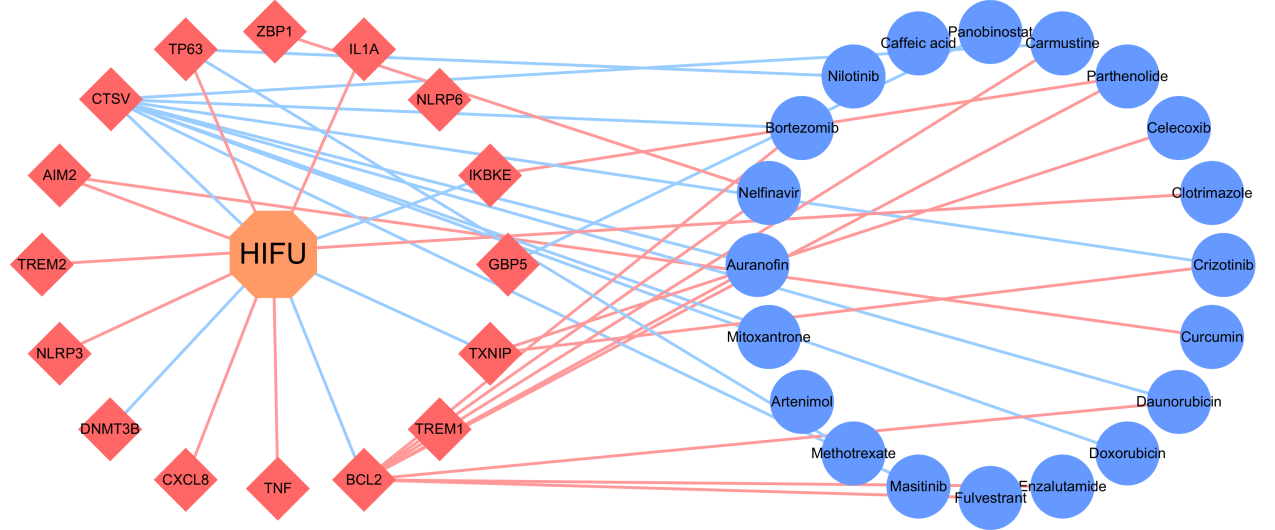


Figure S6. A network diagram illustrating the interactions between HIFU, genes, and drugs in MDA-MB-231 cells (red line: positive correlation, blue line: negative correlation).

| Doxorubicin | Bortezomib | Celecoxib |
| --- | --- | --- |
| Crizotinib | Fulvestrant | Mitoxantrone |
| Caffeic acid | Carmustine | Clotrimazole |
| Auranofin | Daunorubicin | Masitinib |
| Methotrexate | Nelfinavir | Parthenolide |
| Nilotinib | Curcumin | Enzalutamide |
| Artenimol | Panobinostat |  |

Figure S7. Identification of 20 potential drugs with synergistic effects on HIFU therapy through analysis of drug interactions and pyroptosis-related gene networks.


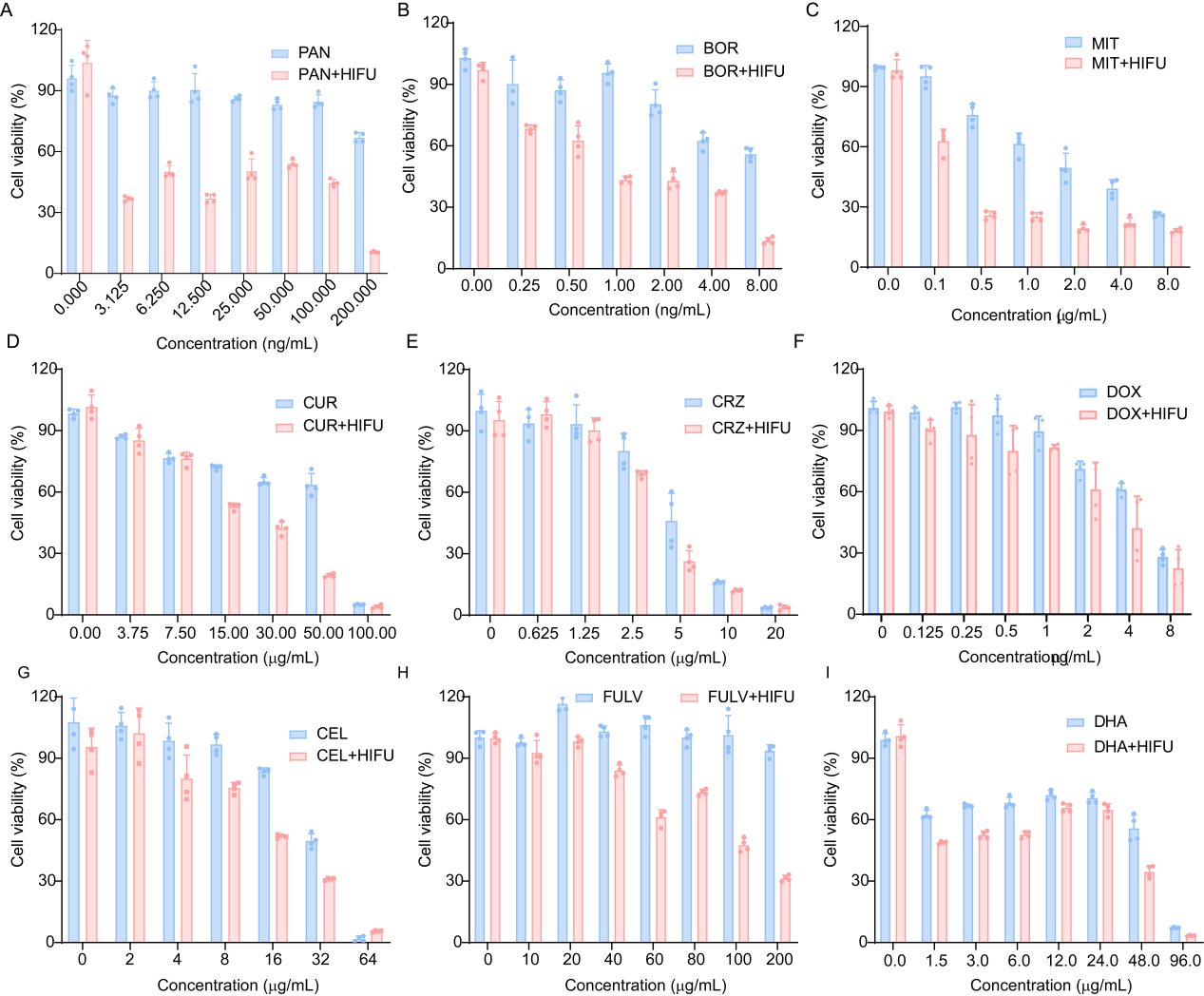


Figure S8. Cell viability of 4T1 cells by different drugs (Panobinostat (PAN), Bortezomib (BOR), Mitoxantrone (MIT), Curcumin (CUR), Crizotinib (CRZ), Doxorubicin hydrochloride (DOX), Celecoxib (CEL), Fulvestrant (FULV), Artenimol (DHA) ) combined with HIFU (n = 4).


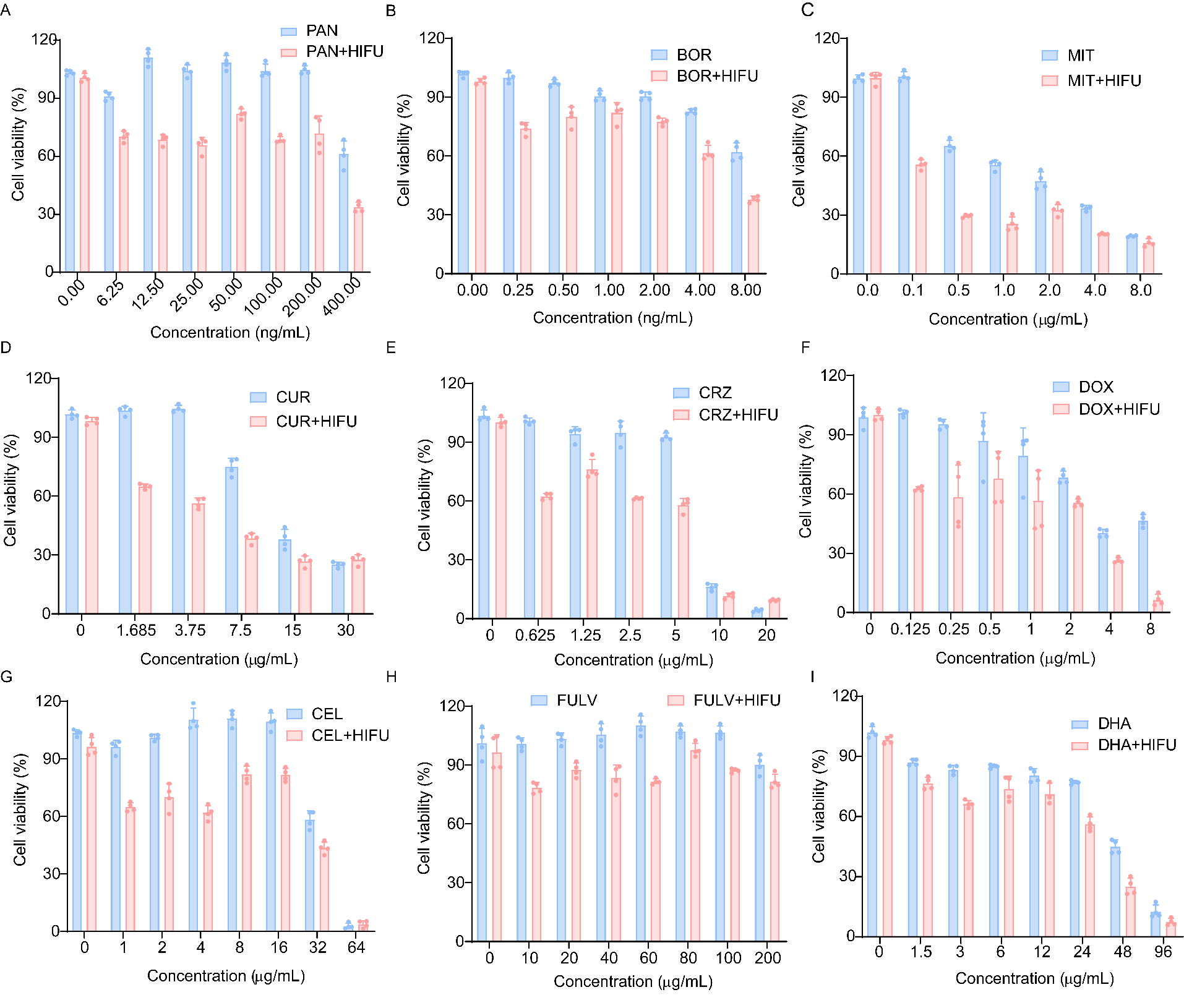


Figure S9. Cell viability of MDA-MB-231 cells by different drugs (Panobinostat (PAN), Bortezomib (BOR), Mitoxantrone (MIT), Curcumin (CUR), Crizotinib (CRZ), Doxorubicin hydrochloride (DOX), Celecoxib (CEL), Fulvestrant (FULV), Artenimol (DHA) ) combined with HIFU (n = 4).


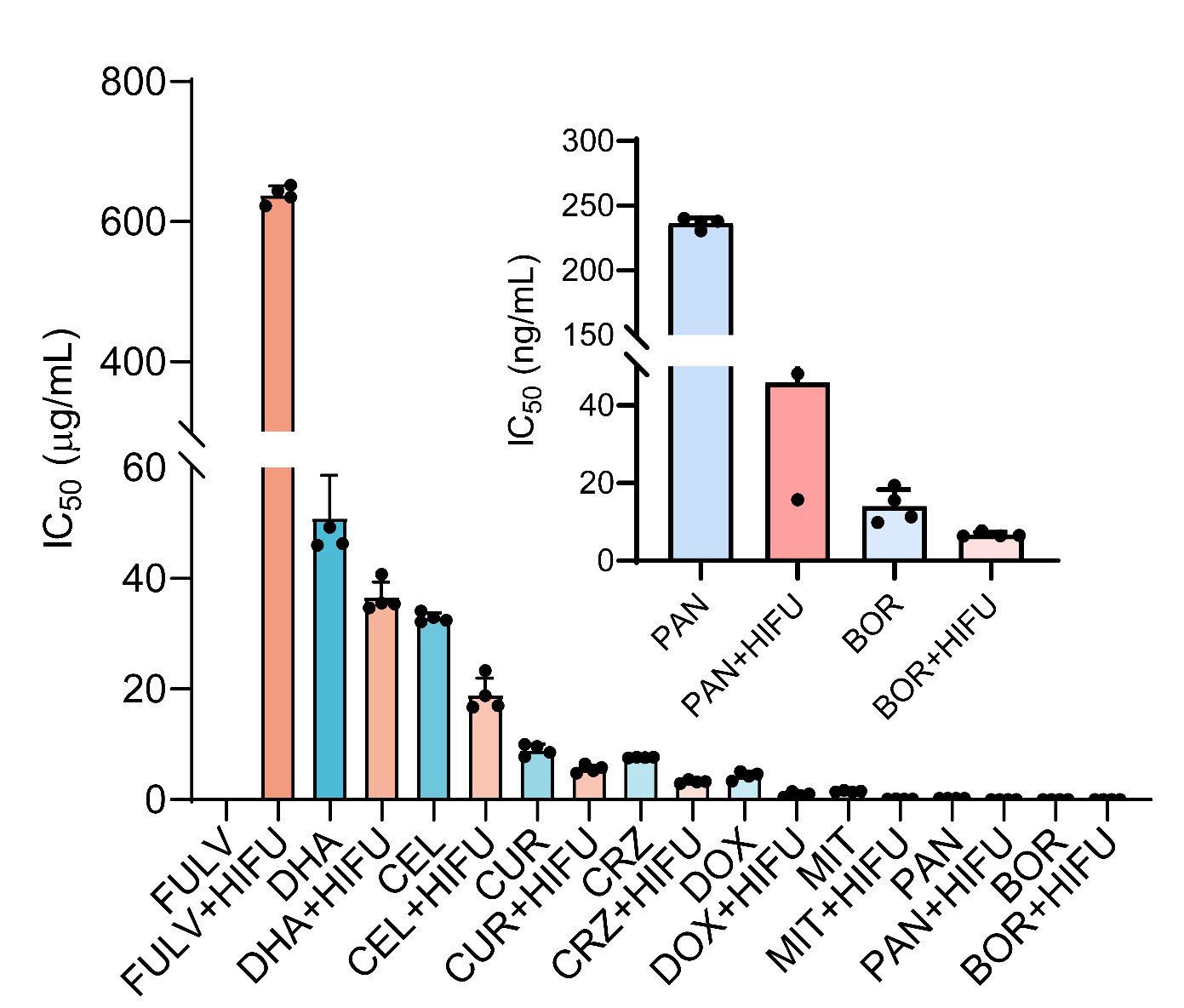


Figure S10. IC50 values after treatment of MDA-MB-231 cells with different drugs (n = 4).


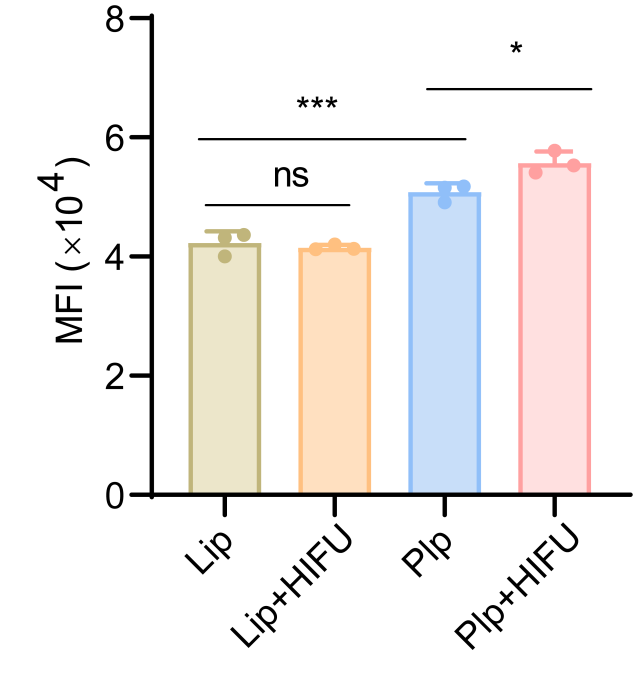


Figure S11. Fluorescence intensity of cellular uptake after 4 h of Lip and Plp treatment (n = 3). Data are presented as mean ± SD. Statistical significance was defined as ^*^*p* < 0.05, ^***^*p* < 0.001; “ns” indicates not significant.


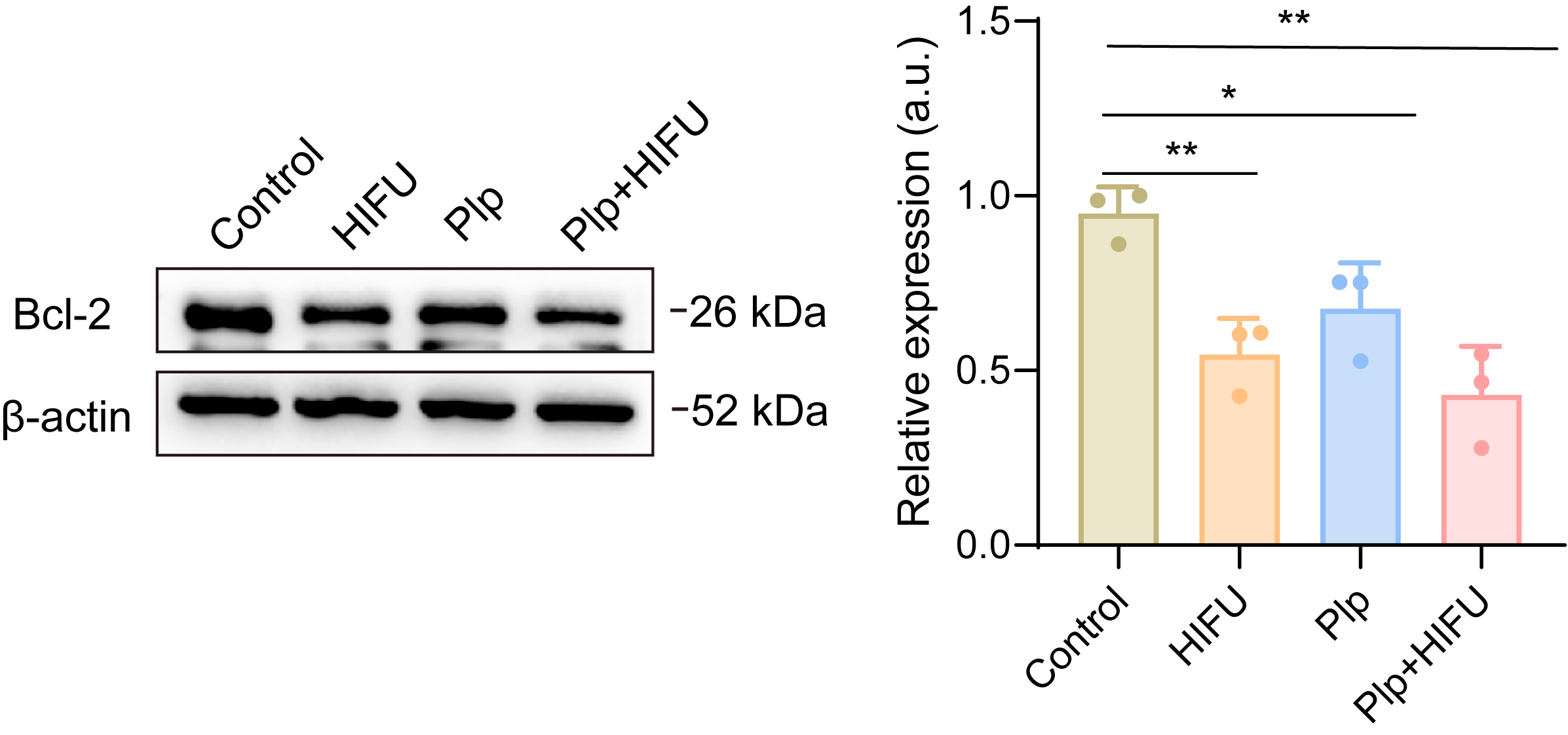


Figure S12. western blot detection of Bcl-2 expression in 4T1 cells and corresponding semi-quantitative analysis (n = 3). Data are presented as mean ± SD. Statistical significance was defined as ^*^*p* < 0.05, ^**^*p* < 0.01, ^***^*p* < 0.001.


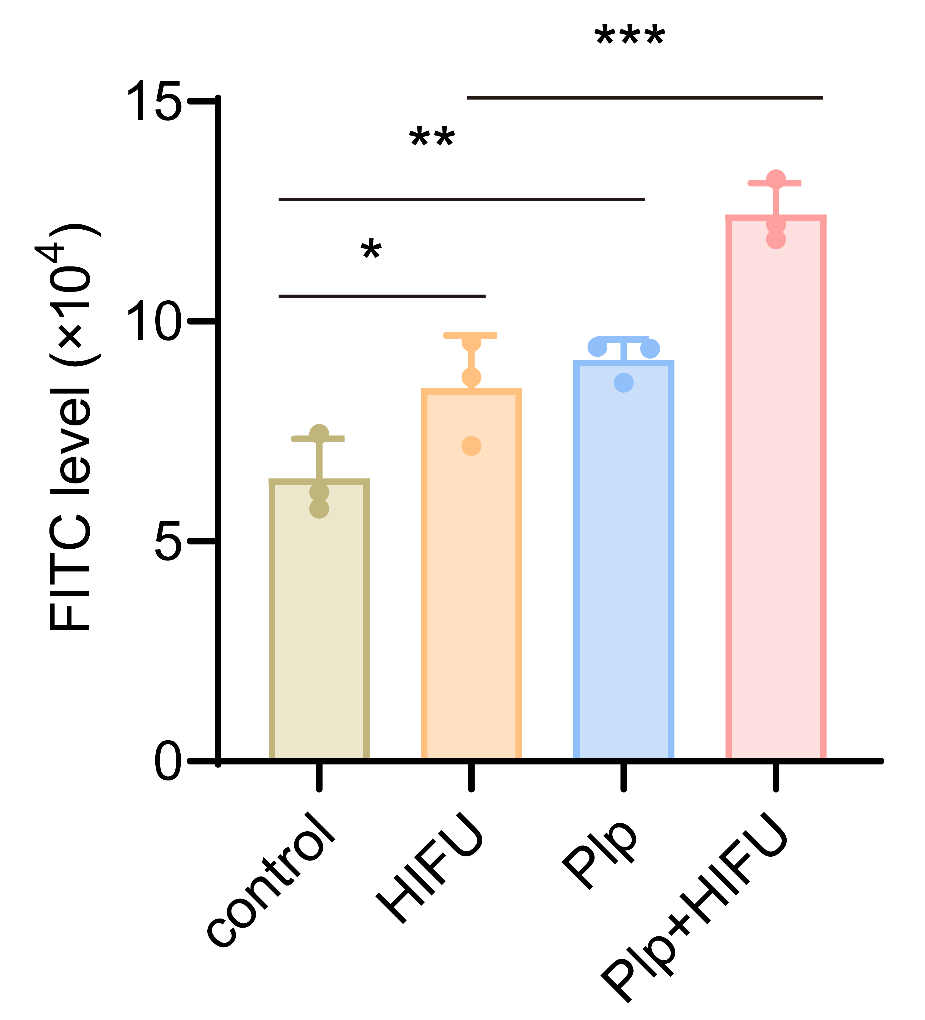


Figure S13. Fluorescence intensity of ROS analyzed by flow-through after different treatments (n = 3). Data are presented as mean ± SD. Statistical significance was defined as ^*^*p* < 0.05, ^**^*p* < 0.01, ^***^*p* < 0.001.


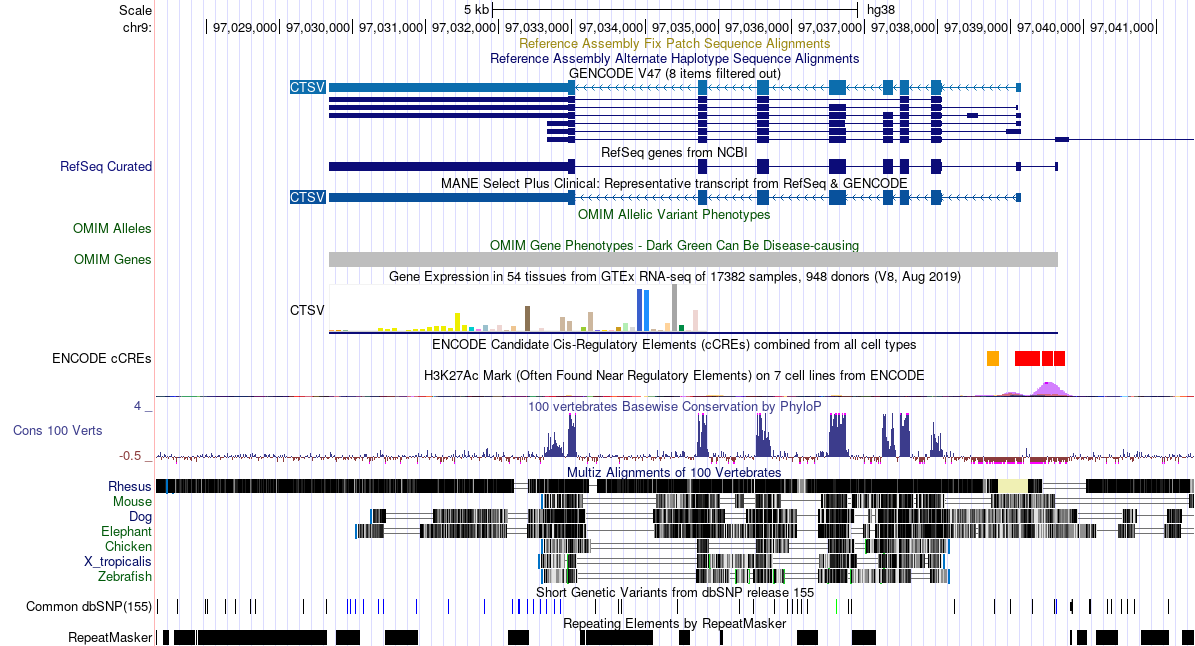


Figure S14. Predicting the acetylation site of the CTSV gene (UCSC Genome Browser Home).


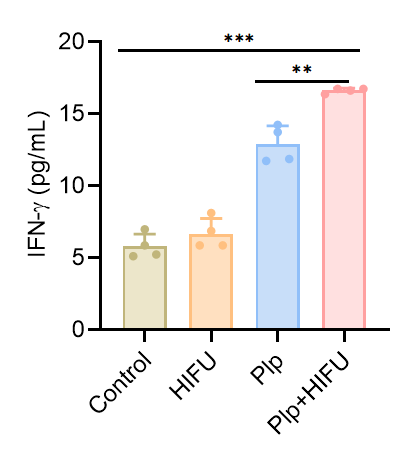


Figure S15. Secretion of IFN-γ inflammatory factor after co-culture of 4T1 cells and BMDC (n=4). Data are presented as mean ± SD. Statistical significance was defined as ^**^*p* < 0.01, ^***^*p* < 0.001.


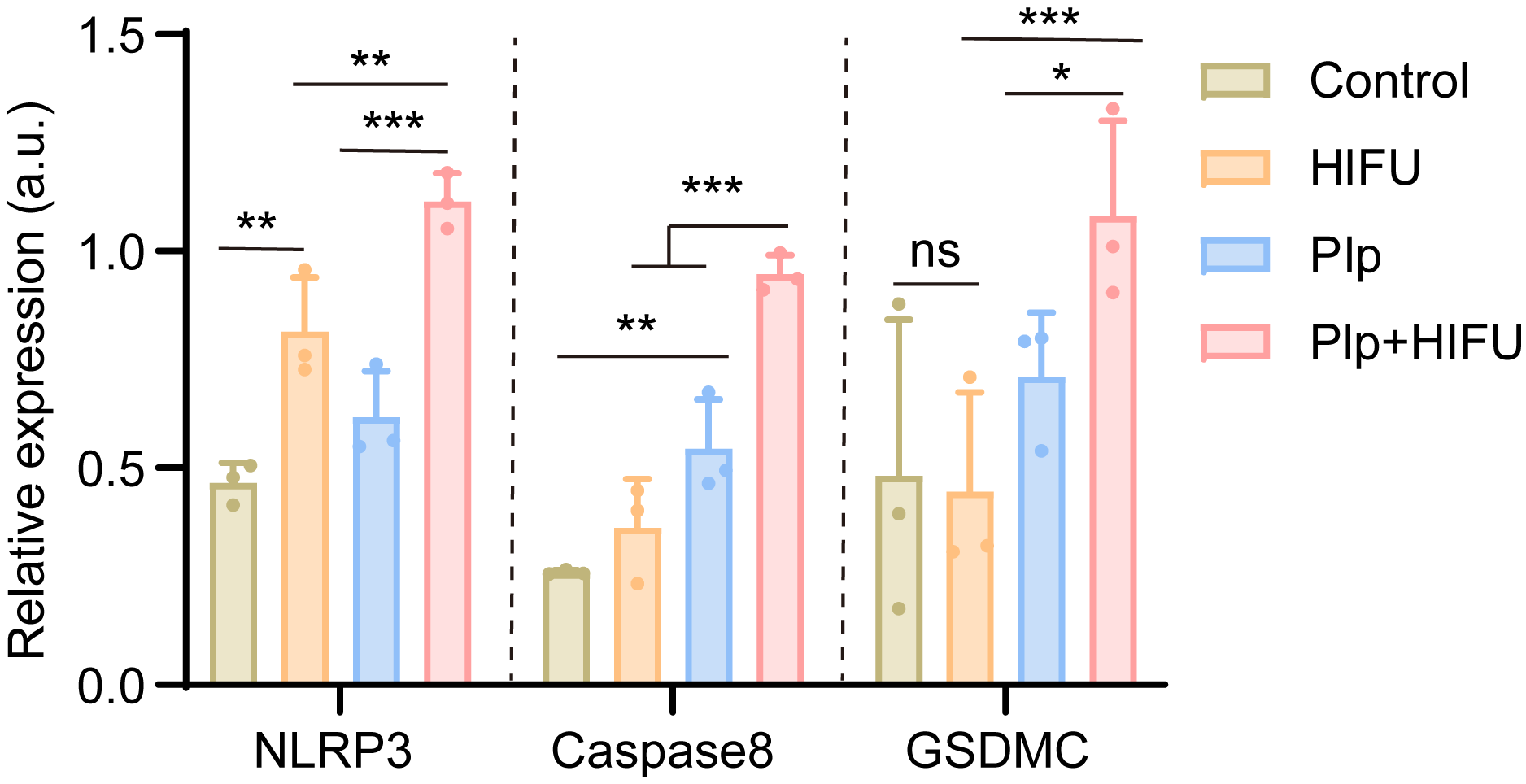


Figure S16. Semi-quantitative analysis of NLRP3, Caspase8 and GSDMC (n = 3). Data are presented as mean ± SD. Statistical significance was defined as ^*^*p* < 0.05, ^**^*p* < 0.01, ^***^*p* < 0.001; “ns” indicates not significant.


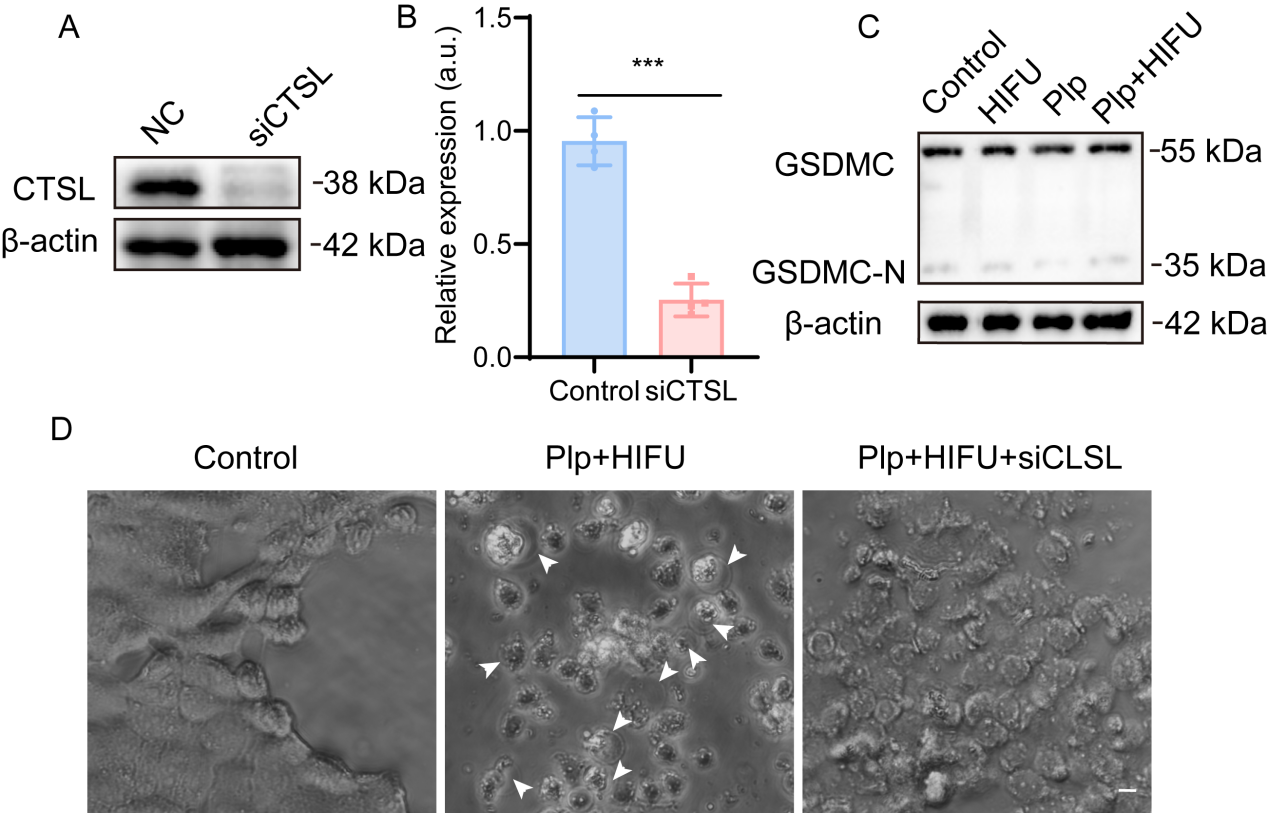


Figure S17. A) Western blot analysis of siRNA targeting CTSL gene degradation in 4T1 cells and corresponding semi-quantitative analysis (B) (n = 3). C) Western blot analysis of siRNA targeting CTSL in 4T1 cells. D) Representative microscopic images of 4T1 cells treated with HIFU and Plp in the presence or absence of siCTSL transfection. White arrows indicate large bubbles emerging from the plasma membrane (scale bar = 20 μm). Data are presented as mean ± SD. Statistical significance was defined as ^***^*p* < 0.001.


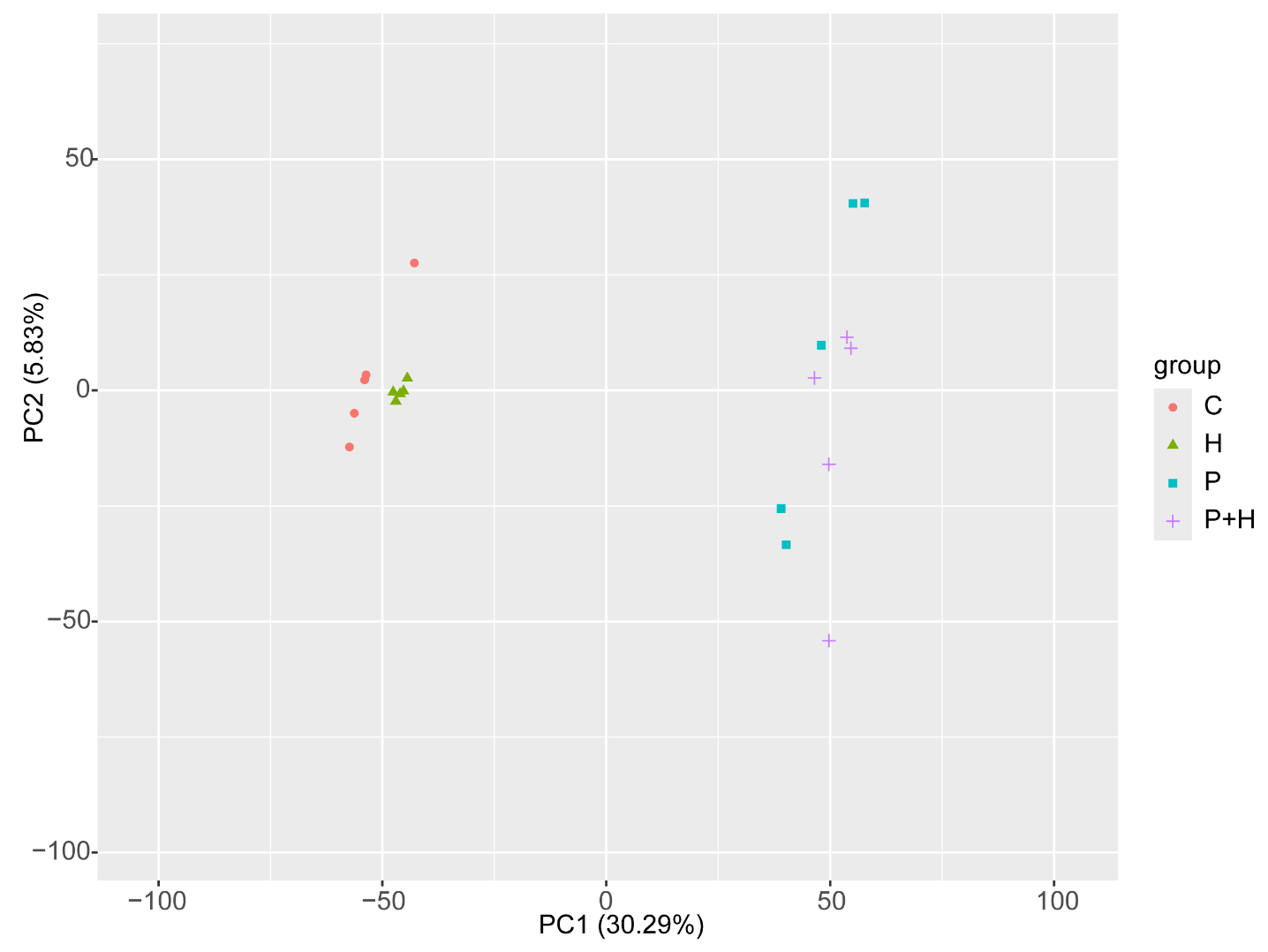


Figure S18. Transcriptomics were analyzed by principal component analysis (PCA), with each point representing a biological replicate (n = 5). (C = Control, H = HIFU, P = Plp, P+H = Plp+HIFU).


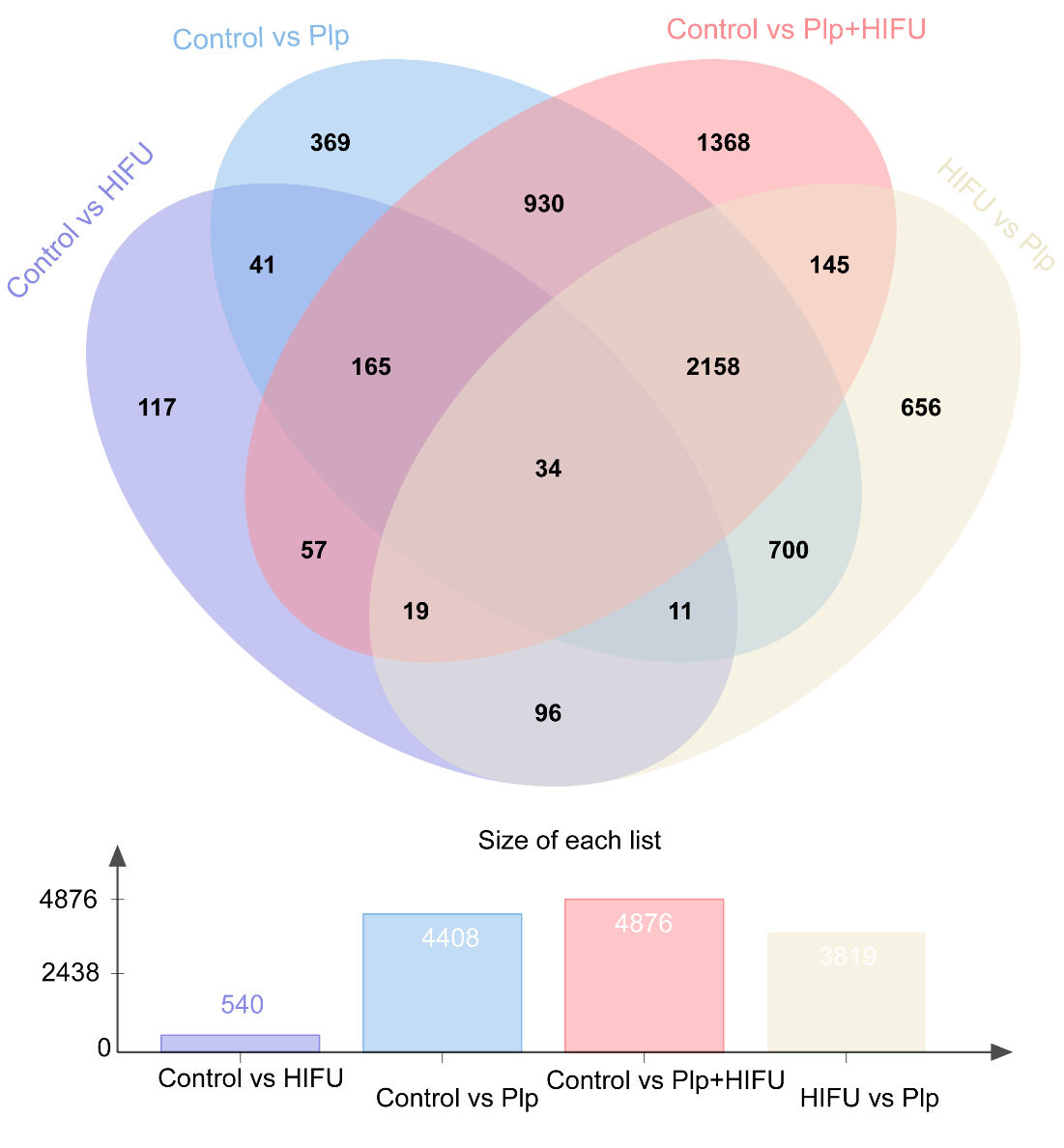


Figure S19. Venn diagram displaying discrepancies and overlapping genes between control, HIFU, Plp and Plp+HIFU groups (n = 5).


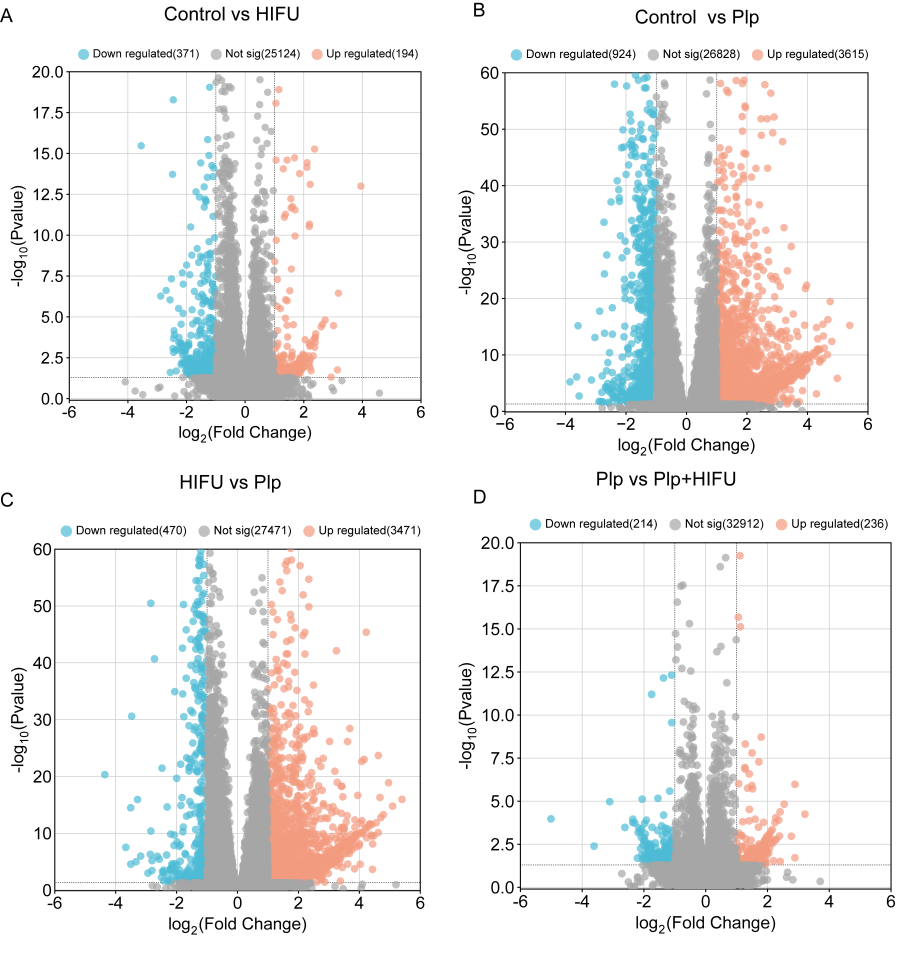


Figure S20. Volcano plot showing up- and down-regulation of genes after different treatments (n = 5). Screening criteria were p < 0.05 and |log2 fold change| => 1.


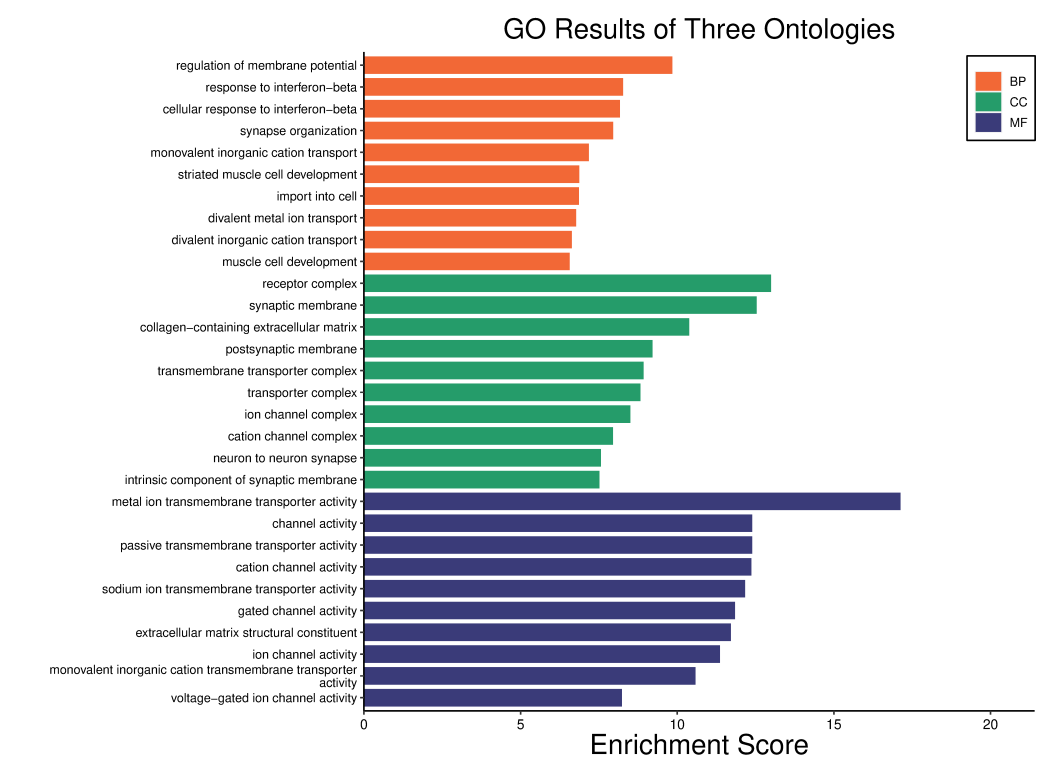


Figure S21. GO analyzed the functional enrichment of differentially expressed genes between Plp+HIFU and controls (n = 5).


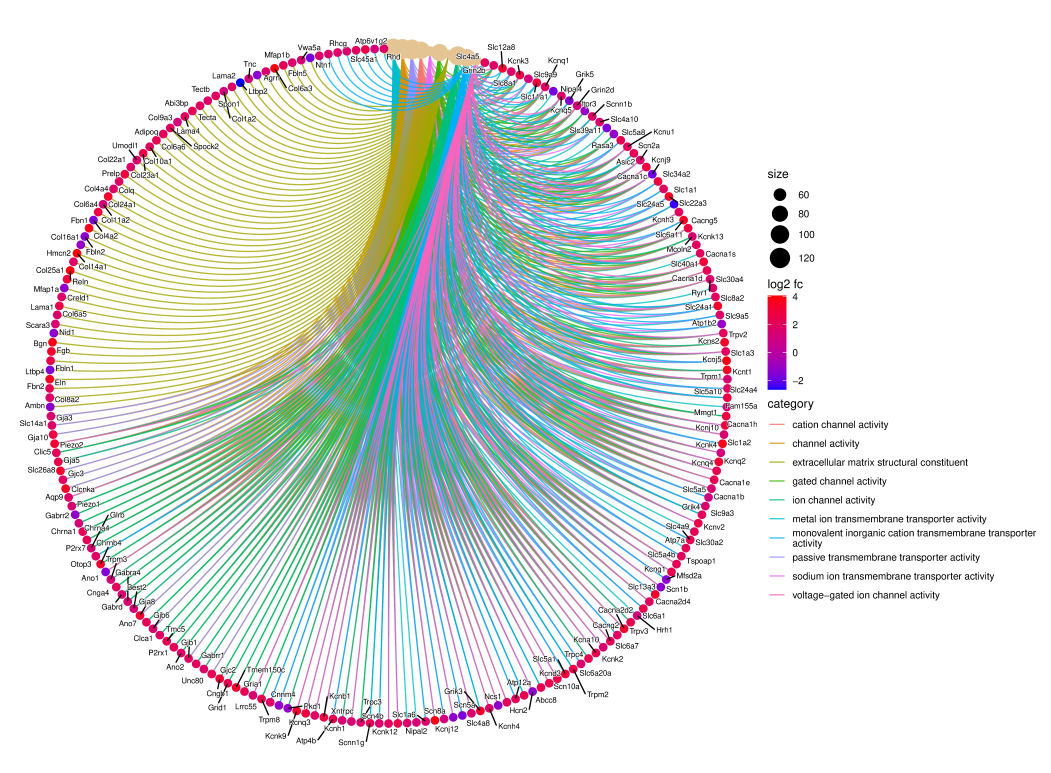


Figure S22. MF analysis reveals pathway enrichment of differentially expressed genes between Plp+HIFU and controls (n = 5).


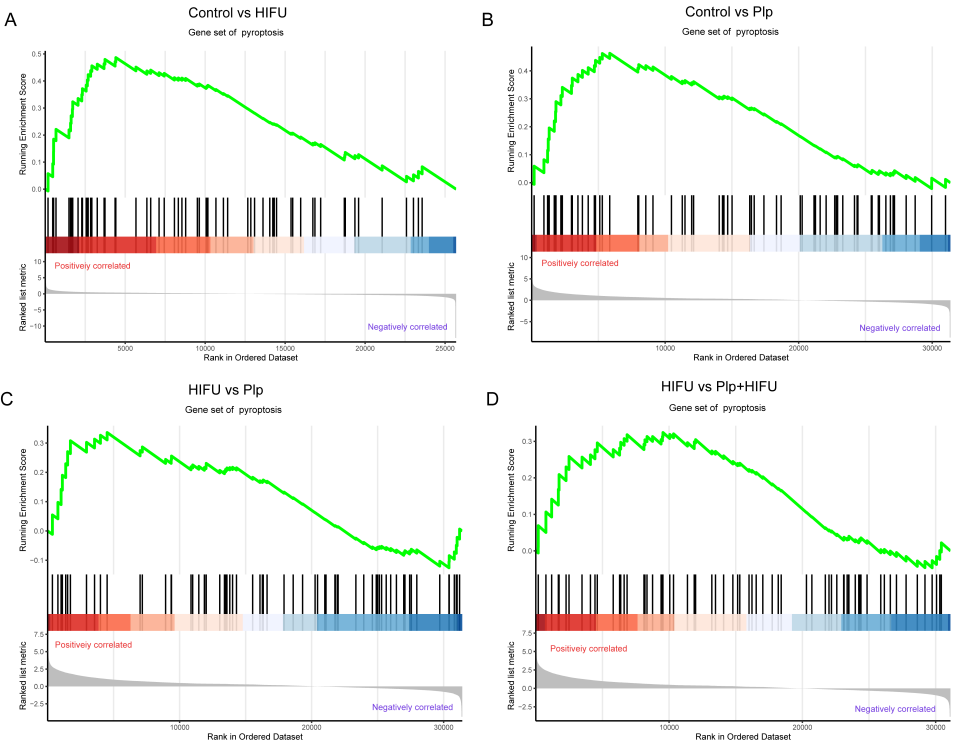


Figure S23. GSEA analysis of pyroptosis-related genes after various groups of treatment (n = 5).


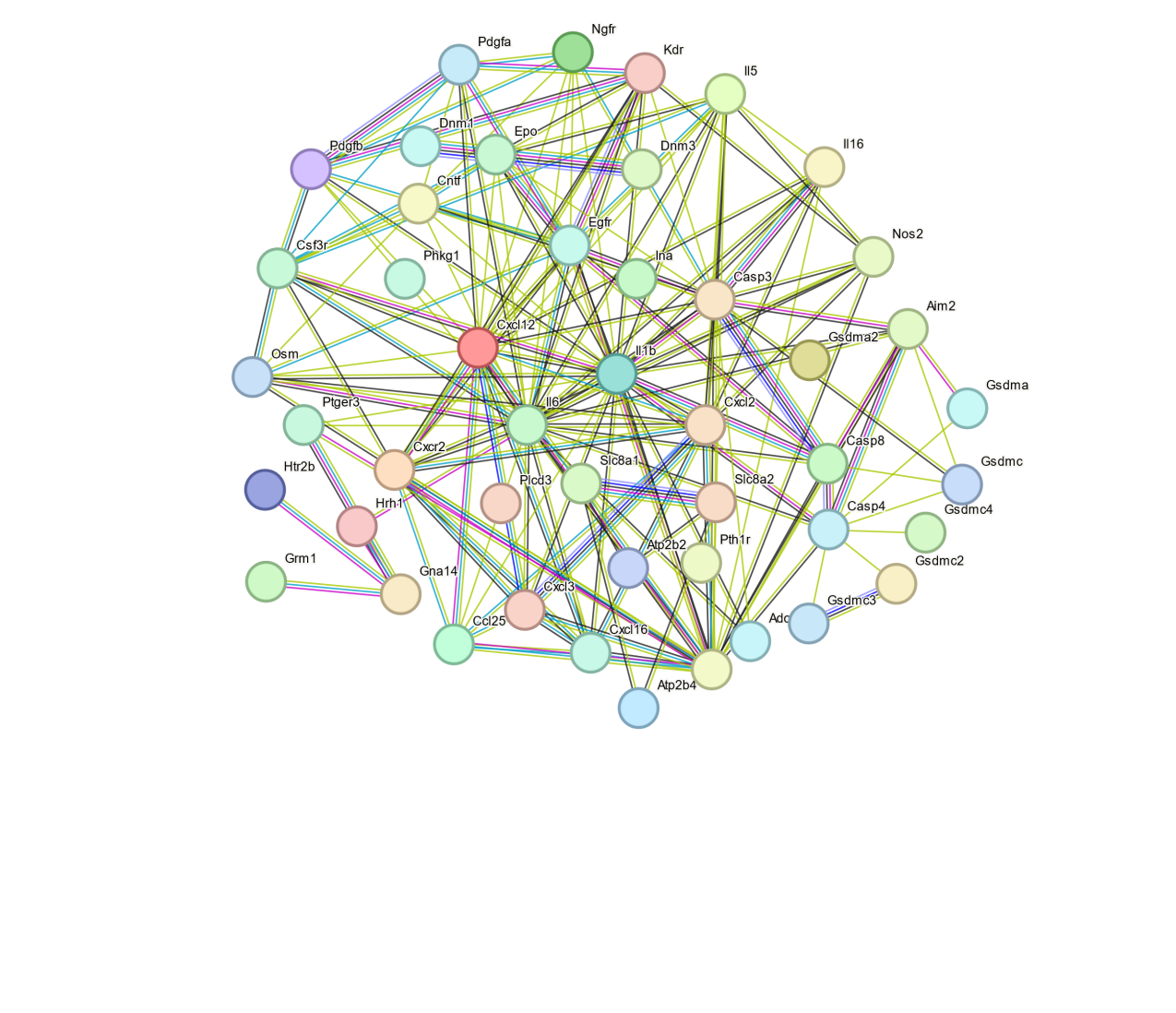


Figure S24. The Search Tool for The Retrieval of Interacting Genes/Proteins (STRING) algorithm was used to analyze gene functional interaction networks.


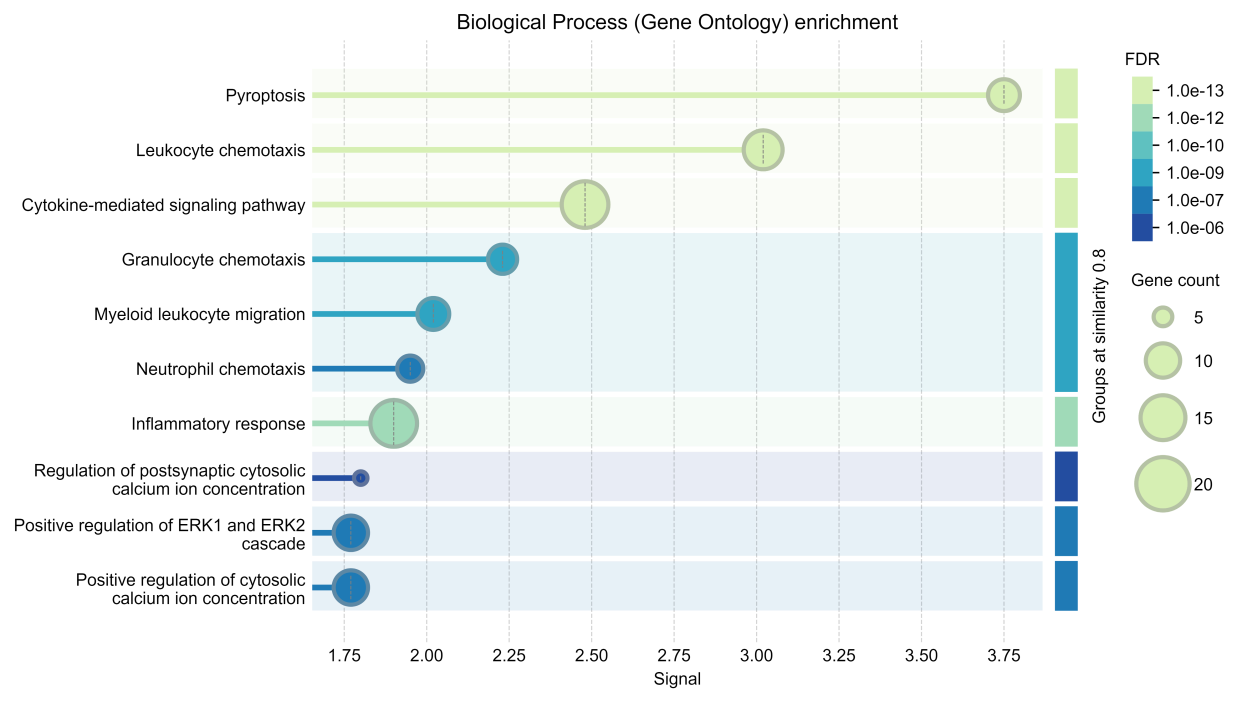


Figure S25. Biological process enrichment analysis of key signaling pathways.


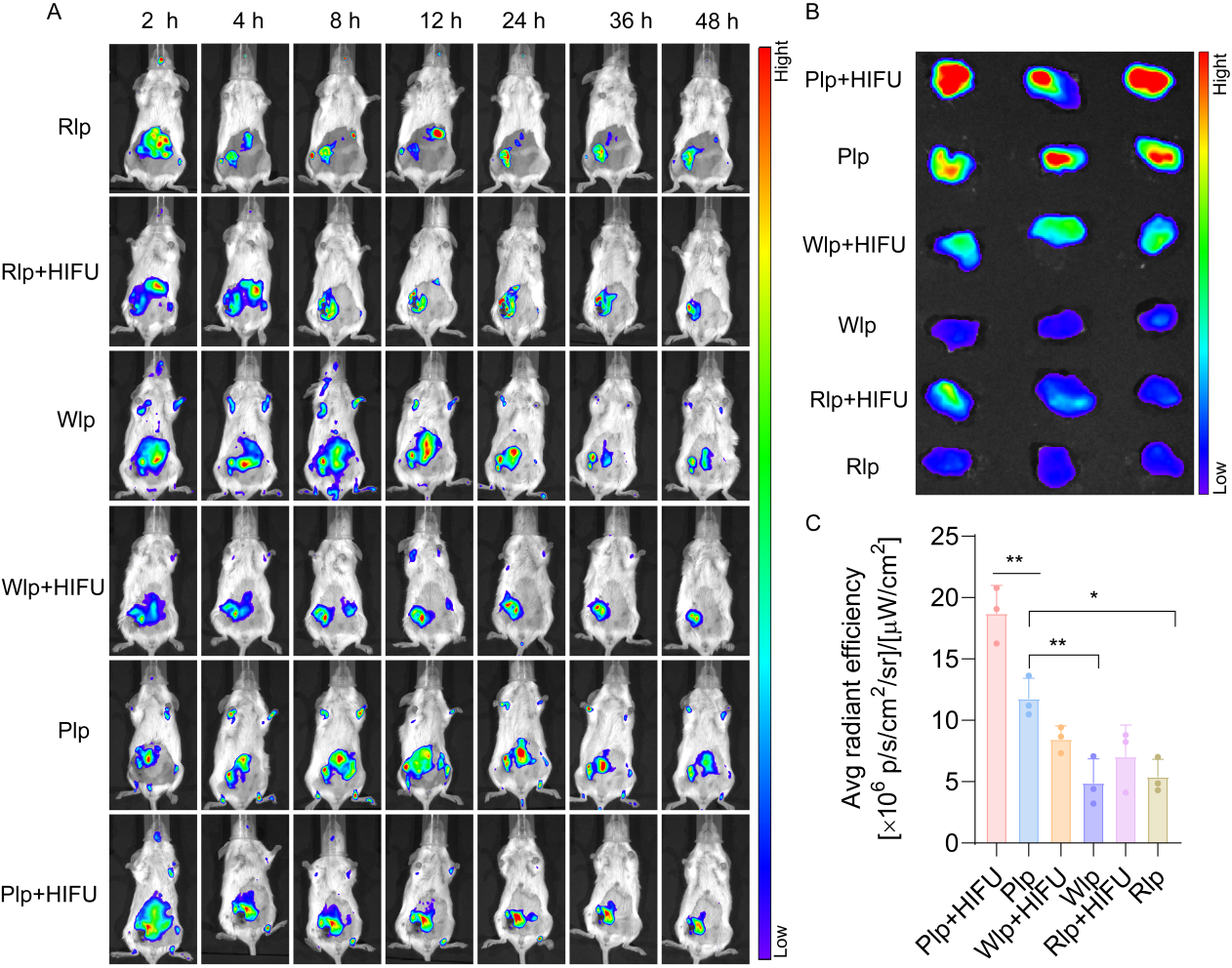


Figure S26. A) Fluorescence imaging images of erythrocyte membrane heterodimeric liposomes (Rlp), leukocyte membrane heterodimeric liposomes (Wlp), and platelet membrane heterodimeric liposomes (Plp) at various time points after combined HIFU administration (n=3). B) *Ex vivo* fluorescence imaging of excised tumors and corresponding quantification of fluorescence intensity 24 h post-injection (n = 3).


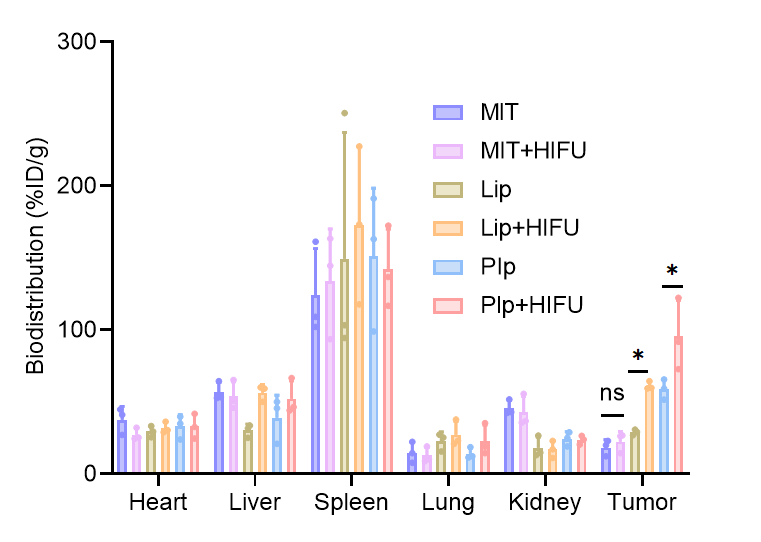


Figure S27. Quantitative biodistribution of Plp in major organs and tumors 24 h post-injection (n = 3). Data are presented as mean ± SD. Statistical significance was defined as ^*^*p* < 0.05; “ns” indicates not significant.


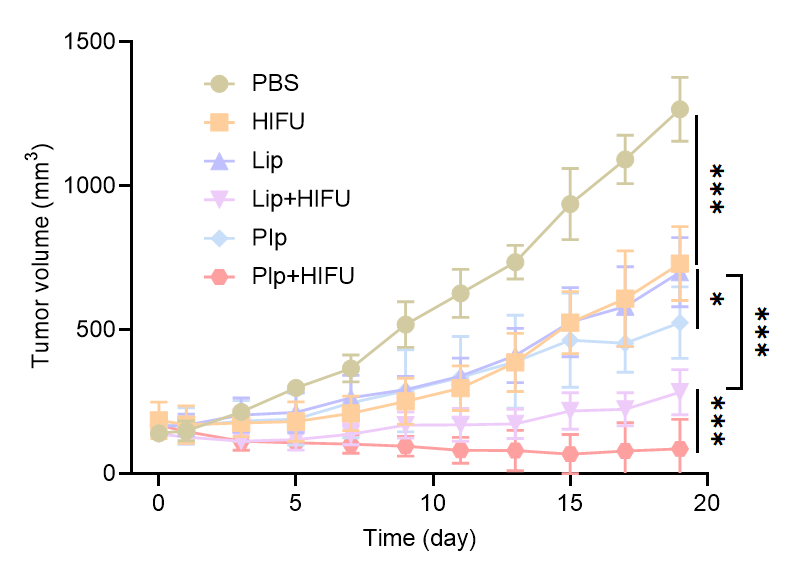


Figure S28. Tumor volume change curves after different treatments (n = 6). Data are presented as mean ± SD. Statistical significance was defined as ^*^*p* < 0.05, ^***^*p* < 0.001.


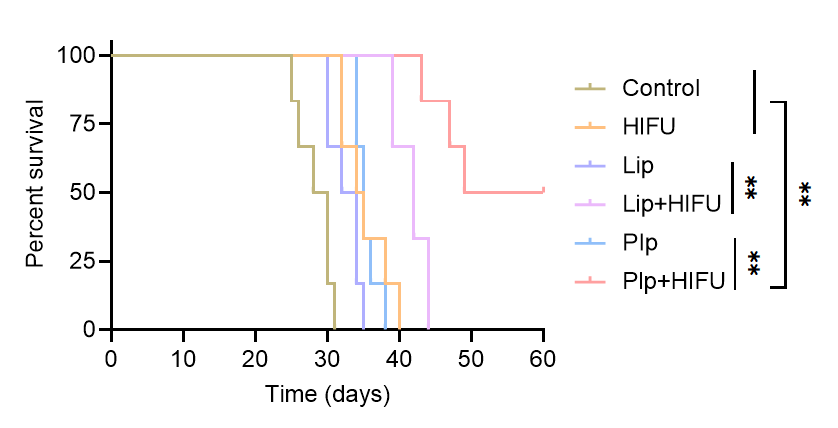


Figure S29. Survival analysis after treatment in different groups (n=6). Data are presented as mean ± SD. Statistical significance was defined as ^**^*p* < 0.01.


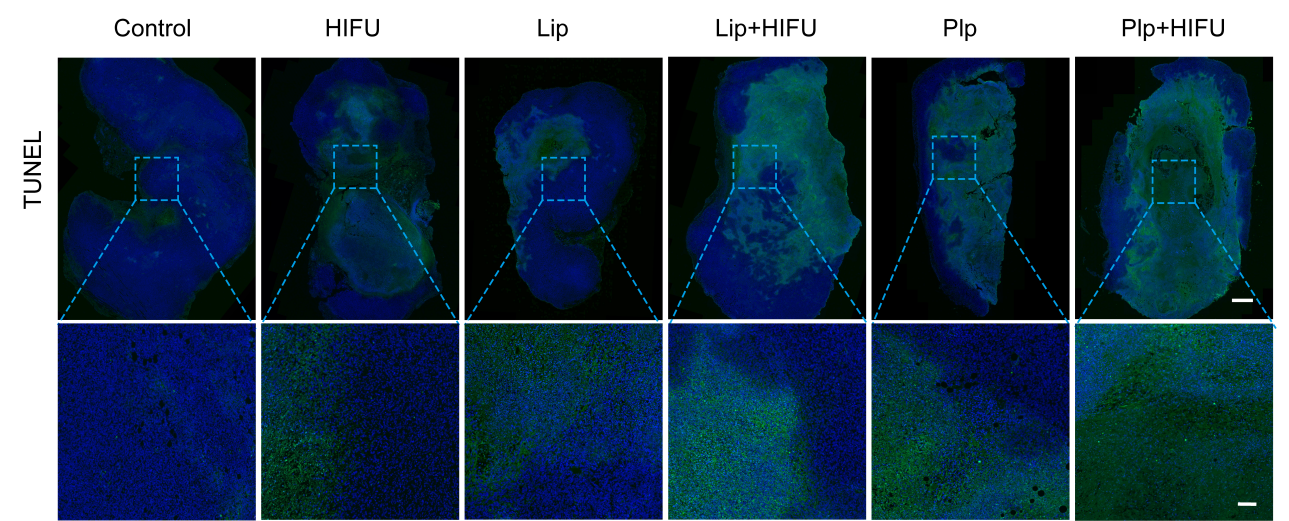


Figure S30. TUNEL staining after treatment in different groups (Scale bar for the upper panel = 1 mm, and scale bar for the lower panel = 100 μm).


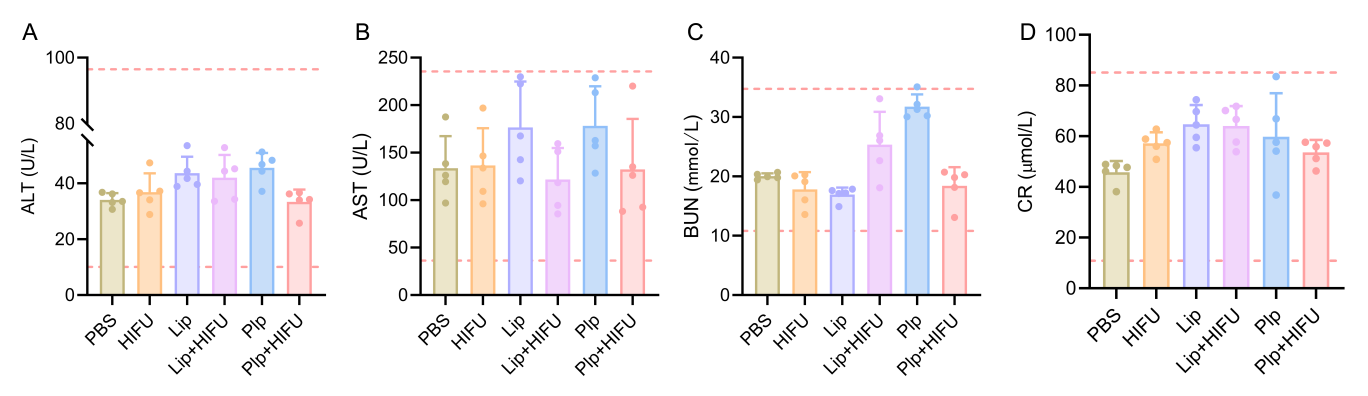


Figure S31. Aspartate aminotransferase (AST), alanine aminotransferase (ALT), blood urea nitrogen (BUN), and creatinine (CR) levels were evaluated in each treatment group to assess liver and kidney function (n = 5).


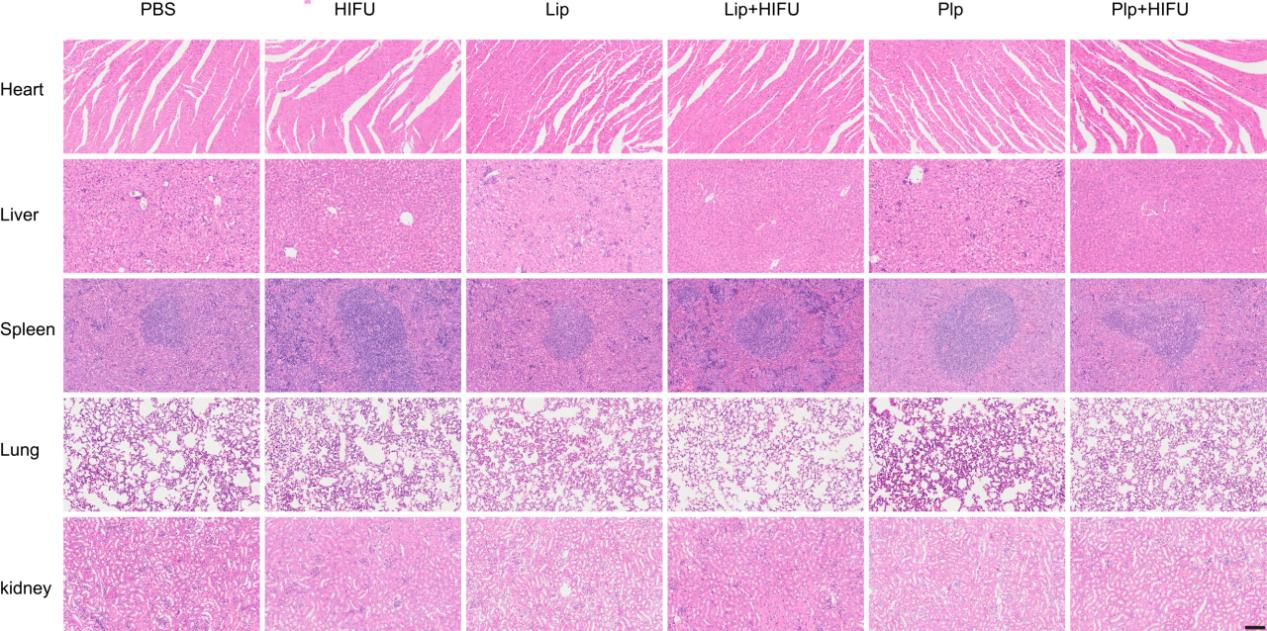


Figure S32. H&E staining of major organs after different treatments (scale bar = 100 μm).


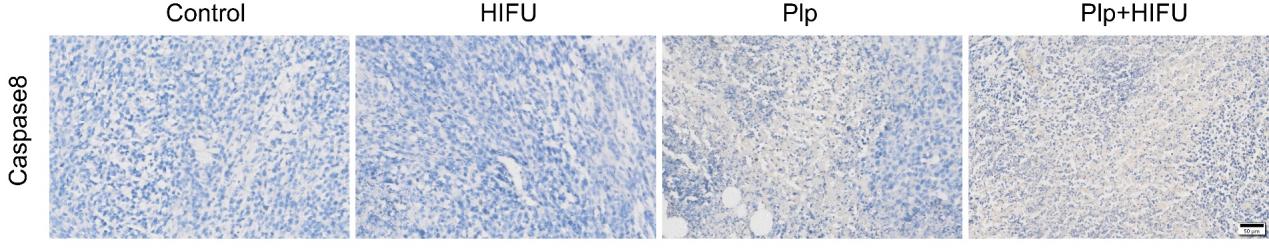
Figure S33. The immunohistochemical image of Caspase8 in 4T1 tumor-bearing mice on day 7 post-treatment (scale bar = 50 μm).


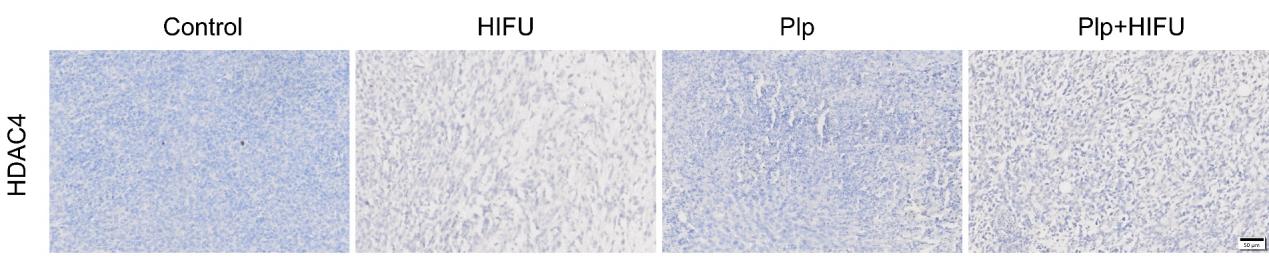
Figure S34. The immunohistochemical image of HDAC4 in 4T1 tumor-bearing mice on day 7 post-treatment (scale bar = 50 μm).


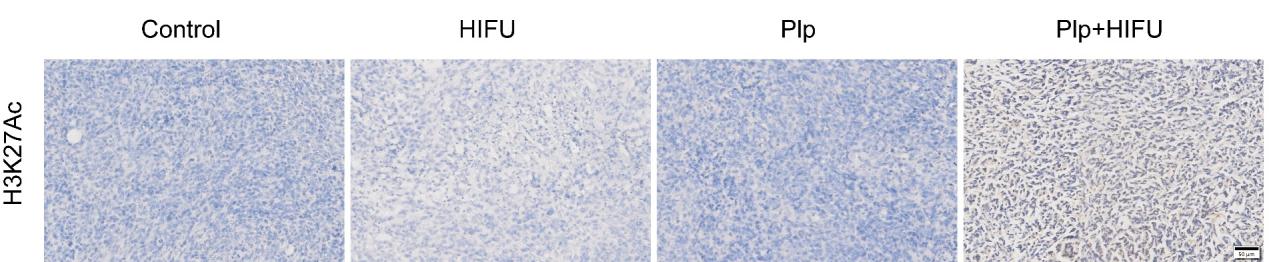
Figure S35. The immunohistochemical image of H3K27Ac in 4T1 tumor-bearing mice on day 7 post-treatment (scale bar = 50 μm).


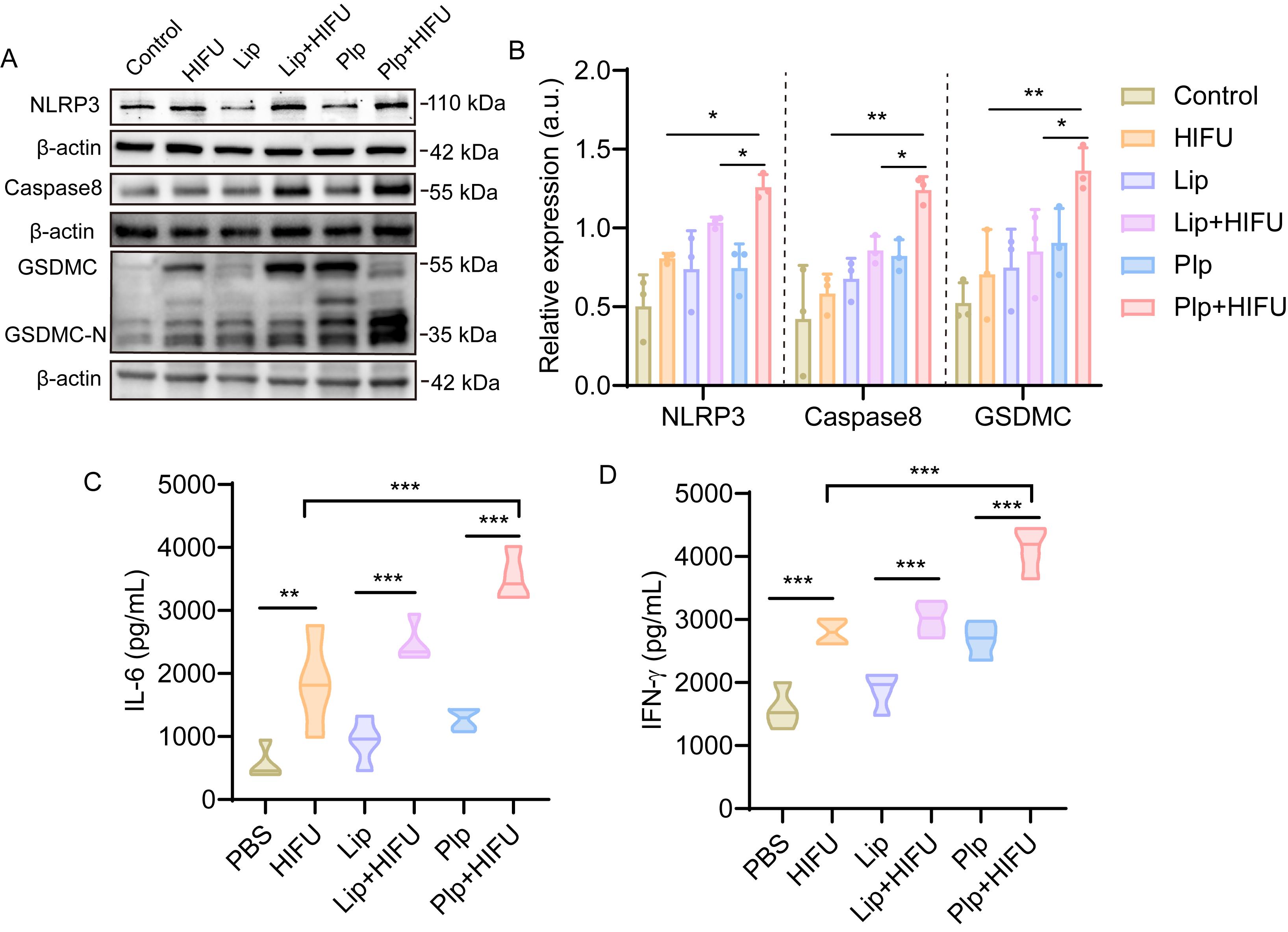


Figure S36. A) Western blot analysis demonstrated the protein expression of pyroptosis-associated markers (NLRP3, Caspase8, and GSDMC) in treated 4T1 tumors, with semi-quantitative analysis shown in (B) (n = 3). The levels of inflammatory cytokines IL-6 (C) and IFN-γ (D) in tumor tissues were assessed by ELISA (n=4). Data are presented as mean ± SD. Statistical significance was defined as ^*^*p* < 0.05, ^**^*p* < 0.01, ^***^*p* < 0.001.


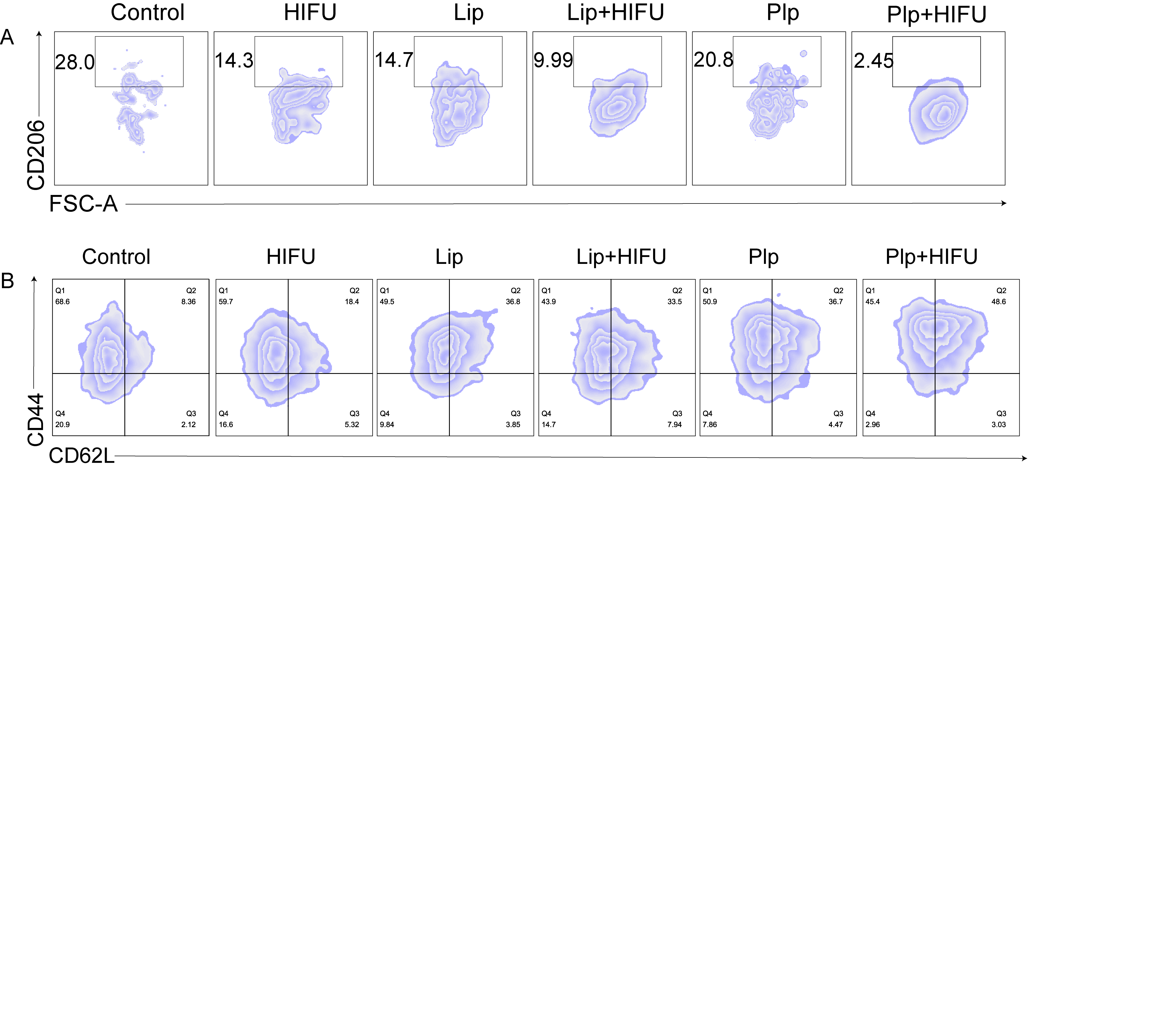


Figure S37. A) Representative flow cytometry plots of M2 macrophages in tumor tissues (n = 4). B) Representative flow cytometry plots of CD44⁺CD62L⁺ memory T cells gated on CD8⁺ T cells in the spleen (n = 4).
